# Integrated genomic analysis identifies a genetic mutation model predicting response to immune checkpoint inhibitors in melanoma

**DOI:** 10.1002/cam4.3481

**Published:** 2020-09-24

**Authors:** Junjie Jiang, Yongfeng Ding, Mengjie Wu, Yanyan Chen, Xiadong Lyu, Jun Lu, Haiyong Wang, Lisong Teng

**Affiliations:** ^1^ Department of Surgical Oncology The First Affiliated Hospital Zhejiang University School of Medicine Hangzhou China; ^2^ Department of Medical Oncology The First Affiliated Hospital Zhejiang University School of Medicine Hangzhou China

**Keywords:** biomarker, durable clinical benefit, immune checkpoint inhibitors, melanoma

## Abstract

Several biomarkers such as tumor mutation burden (TMB), neoantigen load (NAL), programmed cell‐death receptor 1 ligand (PD‐L1) expression, and lactate dehydrogenase (LDH) have been developed for predicting response to immune checkpoint inhibitors (ICIs) in melanoma. However, some limitations including the undefined cut‐off value, poor uniformity of test platform, and weak reliability of prediction have restricted the broad application in clinical practice. In order to identify a clinically actionable biomarker and explore an effective strategy for prediction, we developed a genetic mutation model named as immunotherapy score (ITS) for predicting response to ICIs therapy in melanoma, based on whole‐exome sequencing data from previous studies. We observed that patients with high ITS had better durable clinical benefit and survival outcomes than patients with low ITS in three independent cohorts, as well as in the meta‐cohort. Notably, the prediction capability of ITS was more robust than that of TMB. Remarkably, ITS was not only an independent predictor of ICIs therapy, but also combined with TMB or LDH to better predict response to ICIs than any single biomarker. Moreover, patients with high ITS harbored the immunotherapy‐sensitive characteristics including high TMB and NAL, ultraviolet light damage, impaired DNA damage repair pathway, arrested cell cycle signaling, and frequent mutations in *NF1* and *SERPINB3*/*4*. Overall, these findings deserve prospective investigation in the future and may help guide clinical decisions on ICIs therapy for patients with melanoma.

## Graphical abstract

This study provided evidences that the genetic mutation model (named as ITS) identified a melanoma population with multiple genetic patterns of sensitivity to ICIs, who might potentially benefit from ICIs therapy. Preliminary data from three independent cohorts strongly suggested better treatment outcomes from ICIs therapy in melanoma patients with high ITS. Remarkably, the combination strategy of ITS and TMB or LDH showed better prediction efficacy compared with any single biomarker.

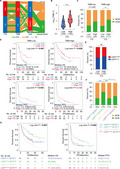

## 1 INTRODUCTION

The treatment and prognosis of metastatic melanoma has shifted dramatically since the advent of immune checkpoint inhibitors (ICIs). Immunotherapeutic antibodies directed at programmed cell‐death protein 1 (PD‑1) and cytotoxic T‑lymphocyte‐associated antigen‑4 (CTLA‑4) are standard therapies for metastatic melanoma.^1^ However, huge disparity in response rates across different populations has reduced the efficacy and accuracy of ICIs therapy, promoting the identification of predictive biomarkers as a hot spot of intense research.^2^ Tumor mutation burden (TMB) has been identified as a promising biomarker of ICIs therapy in diverse cancers.^3^, ^4^, ^5^ High TMB, along with the associated high neoantigen load (NAL), indicates the increased T cell activity and improved response to ICIs.^6^, ^7^ However, several challenges have limited the clinical application of TMB. First, a reliable cut‐off value is still undefined so that high TMB populations cannot be screened out accurately in clinical practice.^8^ Second, the best measurement for TMB is whole‐exome sequencing (WES), not yet applied in clinical practice due to huge cost, intensive time and challenging technology.^9^, ^10^ Besides, the accuracy of measurement for TMB is always influenced by several key factors, including depth of sequencing, length of sequencing reads, choice of aligners, and so on.^8^ Programmed cell‐death receptor 1 ligand (PD‑L1) expression is another primary biomarker of response to ICIs. However, several challenges including the poor reliability for prediction of response, lack of uniformed antibodies for immunohistochemistry, and different thresholds for PD‐L1 positivity have limited the broad application of PD‐L1 expression in clinical practice.^11^ In melanoma, other biomarkers such as lactate dehydrogenase (LDH) and driver mutations in *NRAS* and *NF1*, cannot become independent prognostic indicators for patients treated with ICIs,^12^ although these are associated with response to ICIs as previously reported.^13^, ^14^

In order to identify a clinically actionable biomarker and explore an effective strategy for prediction, we developed a genetic mutation model named as immunotherapy score (ITS) for predicting response to ICIs in melanoma based on three independent cohorts.^15^, ^16^, ^17^ The prediction capability of TMB was also evaluated and compared with that of ITS. In addition, we explored the feasibility and clinical significance of biomarker‐combination strategy based on ITS, TMB, and LDH for predicting response to ICIs. Moreover, we characterized the distinctive genomic patterns associated with ITS.

## 2 MATERIALS AND METHODS

### 2.1 Study design

We conducted a systematic literature search on PubMed, EMBASE, and Web of Science. As a result, three eligible studies were included in this study (Figure S1). Subsequently, we collected and analyzed WES data and clinicopathologic information of 318 melanoma patients treated with ICIs from the included studies, including the Allen cohort,^15^ Snyder cohort,^16^ and Liu cohort.^17^ Based on WES data of the Allen cohort, we performed multivariate logistic regression to construct a genetic mutation model name as immunotherapy score (ITS) for predicting durable clinical benefit (DCB) from ICIs therapy. Then we evaluated the association between ITS and overall survival (OS) or progression‐free survival (PFS) in melanoma patients treated with ICIs. The prediction capability of ITS was validated in the Snyder cohort and Liu cohort. The summary predictive effect and between‐cohort heterogeneity were further estimated by meta‐analysis. Moreover, the prediction capability of TMB was also evaluated and compared with that of ITS. Remarkably, we explored the feasibility and significance of biomarker combination based on ITS, TMB, and LDH for predicting DCB and survival outcomes in melanoma patients with ICIs therapy. Importantly, we characterized distinctive genomic patterns associated with ITS, based on somatic mutation and copy number variation (CNV) data in the Allen cohort, Snyder cohort, and Liu cohort. Besides, we analyzed the association between ITS and prognosis of melanoma patients without ICIs therapy using TCGA‐SKCM cohort and ICGC‐MELA cohort.

### 2.2 Literature search

Systematic literature search was conducted on PubMed, EMBASE, and Web of Science up to June 1, 2020. The search term was as follows: (Melanoma OR Melanomas OR “Malignant Melanoma” OR “Malignant Melanomas” OR “Melanoma, Malignant” OR “Melanomas, Malignant”) AND (PD‐1 OR PD‐L1 OR CTLA‐4 OR “immune checkpoint inhibitor” OR “immune checkpoint inhibitors” OR “ICI” OR “ICIs” OR “immune checkpoint blocker” OR “immune checkpoint blockers” OR “ICB” OR “ICBs” OR Ipilimumab OR Avelumab OR Tremelimumab OR Atezolizumab OR Nivolumab OR Durvalumab OR Pembrolizumab OR Lambrolizumab) AND (WES OR “Whole Exome*” OR “Whole‐exome” OR “Whole Exome Sequencing” OR “Whole Exome Sequencings” OR “Complete Exome Sequencing”). The inclusion criteria for eligible studies were as follows: (a) clinical trials or cohort studies associated with inhibitor of PD‐1/PD‐L1, CTLA‐4, or their combination, in patients with melanoma; (b) Clinical outcomes of patients were available, including objective response, OS, and/or PFS; (c) whole‐exome sequencing(WES) was performed in the original study and the corresponding WES data were available from the databases or the articles; (d) the number of patients accessible for evaluation was more than 50; (e) studies were published in English. Reviews, case reports, editorials, meeting comments, abstracts, and letters were excluded. As a result, the Allen cohort, Snyder cohort, and Liu cohort were included in this study. The workflow of the literature search is shown in Figure S1. Furthermore, we collected two clinical cohorts of non‐small‐cell lung cancer (NSCLC) patients with the ICIs therapy, Miao cohort, and Hellmann cohort,^18^, ^19^ for evaluating the applicability of ITS for NSCLC.

### 2.3 Data collection and preprocessing

We downloaded the clinical information, WES, and RNA‐seq data of the Allen cohort, Snyder cohort, TCGA‐SKCM cohort, and Hellmann cohort from the cBioPortal database (https://www.cbioportal.org/). Data of the Liu cohort and Miao cohort were acquired from supplemental materials of the reported articles.^17^, ^18^ The clinical information and WES data of the ICGC‐MELA cohort were downloaded from the International Cancer Genome Consortium (https://icgc.org/). A total of 955 melanoma and 132 NSCLC patients were included in this study. The Allen cohort consisted of 110 melanoma patients treated with Ipilimumab for model construction.^15^ The Snyder cohort consisted of 64 melanoma patients treated with Ipilimumab or Tremelimumab as a validation dataset.^16^ The Liu cohort consisted of 144 melanoma patients treated with Nivolumab or Pembrolizumab as a validation dataset.^17^ The clinicopathological information of the Allen cohort, Snyder cohort, and Liu cohort included age, gender, tumor primary site, M stage, LDH, received drugs, best objective response, OS, and PFS. Meta‐cohort was composed of the Allen cohort, Snyder cohort, and Liu cohort for multivariate regression and genomic analysis. TCGA‐SKCM cohort and ICGC‐MELA cohort consisted of 355 and 282 melanoma patients without ICIs therapy, respectively.^20^, ^21^ The Hellmann cohort and Miao cohort consisted of 75 and 57 NSCLC patients with ICIs therapy, respectively.^18^, ^19^ We divided the NSCLC patients into squamous and nonsquamous types after merging the Hellmann cohort and Miao cohort. The detailed clinical and genomic characteristics of the cohorts are shown in Table [Link], [Link], [Link].

As stated in the previous studies,^15^, ^16^, ^17^ whole‐exome capture libraries were constructed using the Agilent SureSelect All Exon v2 or 50‐Mb kit. The exome libraries were sequenced on the Illumina HiSeq 2000 or 2500 platform to generate paired‐end reads (2 × 76 bp) and reach 178X mean target coverage (range 32–380). The synonymous mutation was excluded in the variants of gene mutation. The nonsynonymous mutation in gene‐coding regions included missense, nonsense, deletion, insertion, and splice mutations. RNA‐seq data were the type of fragments per kilobase of exon per million fragments mapped (FPKM), then normalized to transcripts per million (TPM) and Z‐score for further analysis.

### 2.4 Definition of clinical end points

In this study, DCB, OS, and PFS were adopted as clinical endpoints to evaluate the response to ICIs for patients. DCB was defined as a composite endpoint of complete response (CR) or partial response (PR) to ICIs by RECIST criteria v.1.1^22^ or stable disease (SD)^22^ with PFS more than 6 months. No clinical benefit (NCB) was defined as progressive disease (PD) by RECIST criteria v.1.1^22^ or SD with PFS less than 6 months. Particularly, due to lack of data on PFS and best objective response in the Snyder cohort, DCB was defined by radiographic evidence of freedom from disease or evidence of a stable or decreased volume of disease for more than 6 months according to the original article.^16^ NCB was defined by tumor growth on every computed tomographic scan after the initiation of treatment or a clinical benefit lasting 6 months or less according to the original article.^16^

### 2.5 Construction and validation of genetic mutation model

**FIGURE 1:**
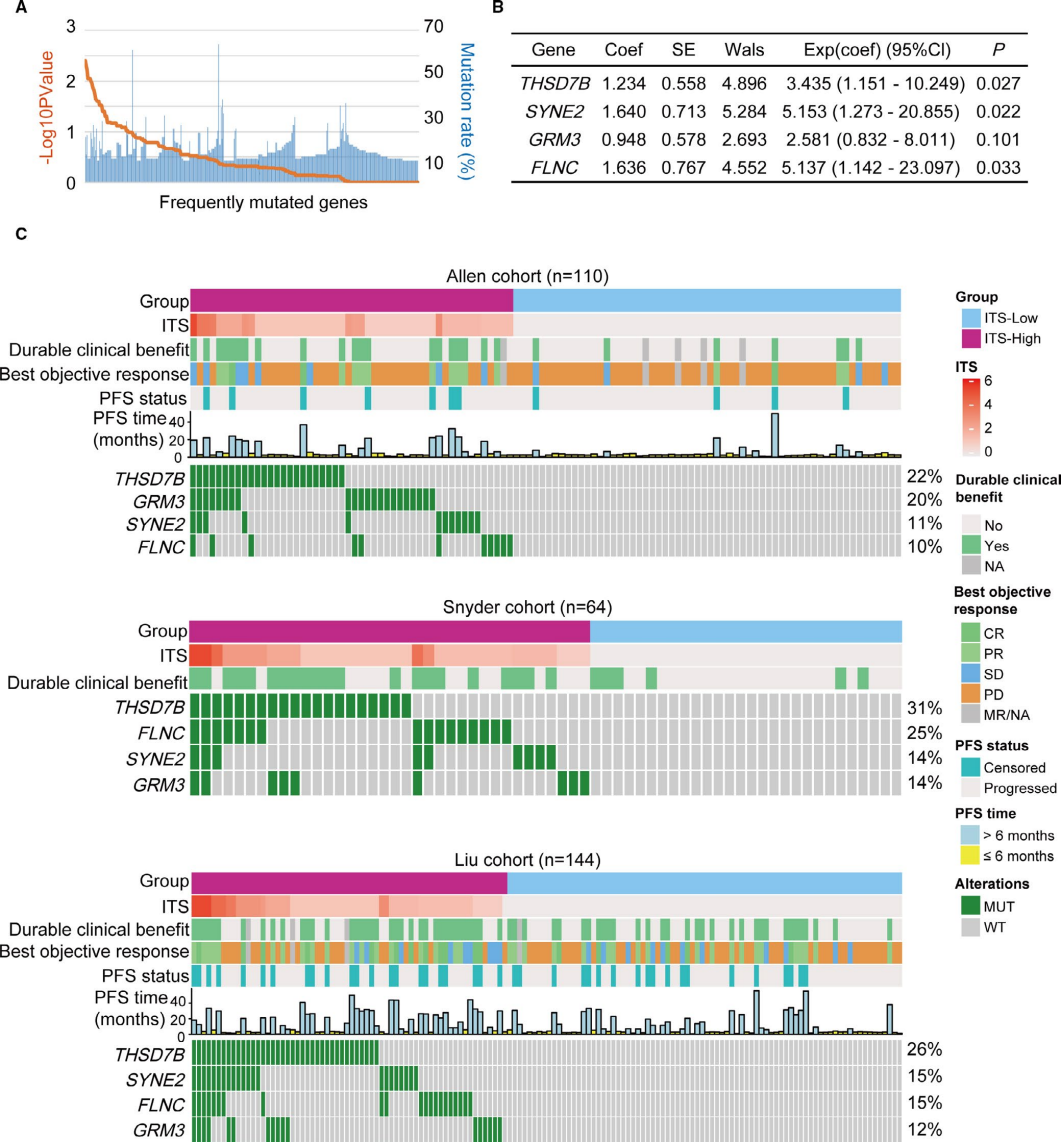
Construction of the genetic mutation model for predicting durable clinical benefit from ICIs therapy in melanoma using multivariate logistic regression analysis. (A) The frequently mutated genes (mutation rate ≥10%) ranked by *p* value. *p* value was calculated to evaluate the association between the frequently mutated genes and durable clinical benefit by a two‐sided *χ* ^2^ test. (B) Genetic mutation model was constructed by multivariate logistic regression analysis for predicting DCB from ICIs therapy. (C) Waterfall plot showing the characteristics associated with the genetic mutation model in the Allen cohort, Snyder cohort, and Liu cohort. The immunotherapy score formula was as follows: ITS = 1.234 × *THSD7B* + 1.640 × *SYNE2* + 0.948 × *GRM3* + 1.636 × *FLNC*. The mutation status was regarded as 1, whereas wild type is 0. ICIs, immune checkpoint inhibitors; ITS, immunotherapy score; DCB, durable clinical benefit; PFS, progression‐free survival; CR, complete response; PR, partial response; SD, stable disease; PD, progressive disease; MR, mixed response; NA, not available; Coef, coefficient; CI, confidence interval; SE, standard error

The frequently mutated genes (mutation rate ≥10%) were screened out from the Allen cohort based on the WES data. Subsequently, the association between the frequently mutated genes and DCB of patients with melanoma was determined by *χ* ^2^ test. The genes reaching the statistical significance of *p* < .05 were regarded as candidate variables to perform multivariate logistic regression analysis by the Backward Elimination (Wald) strategy. As a result, four mutated genes (*THSD7B*, *SYNE2*, *GRM3*, and *FLNC*) were included in the multivariate logistic regression model (Figure 1). The immunotherapy score (ITS) formula was established based on the coefficient (coef) combined with corresponding mutation status of genes as follows: ITS = ∑ (coef_i_ × status_i_), where coef was derived from the multivariate logistic model and the mutation status was equal to 1, whereas wild type was 0. Patients were divided into low (ITS = 0) and high (ITS > 0) group, where the low group showed no nonsynonymous mutation in the genes and the high group showed at least one nonsynonymous mutation in the genes. Moreover, ITS was calculated using the same formula in the Snyder cohort, Liu cohort, Hellmann cohort, and Miao cohort for validation. Receiver operating characteristic (ROC) analysis was performed to assess the predictive accuracy using “pROC” package^23^ with the R software (version 3.6.1). The value of area under the ROC curve (AUC) was used to evaluate the predictive accuracy for DCB from ICIs therapy.

### 2.6 Survival analysis

We explored the impact of TMB and ITS on survival outcomes (OS and PFS) of melanoma patients treated with ICIs in the Allen cohort, Snyder cohort and Liu cohort. In addition, we performed Kaplan–Meier survival analysis for NSCLC patients treated with ICIs. The association between ITS and overall survival of melanoma patients without ICIs therapy was also evaluated in TCGA‐SCKM cohort and ICGC‐MELA cohort. We calculated the median overall survival time and the 95% confidence interval and performed log‐rank test. Log‐rank *p* < .05 was considered as statistically significant.

### 2.7 Logistic and Cox regression analysis

Patients were divided into high ITS and low ITS group according to whether the value of ITS is more than 0. Zero represented no mutation in the four genes, whereas 1 represented at least one mutation. We classified the patients into high and low TMB using the median value and upper quartile as thresholds, respectively. We performed univariate logistic regression analysis to evaluate the association between DCB and ITS or TMB by calculating Odds ratios (OR) and 95% confidence interval (CI). Multivariate logistic regression analysis was applied to investigate the independently predictive effect of ITS and TMB on DCB. In addition, we performed univariate Cox regression analysis to assess the effect of ITS and TMB on OS and PFS by calculating hazards ratio (HR) and 95% CI. Multivariate Cox regression analysis was applied to evaluate the independent effect of ITS and TMB on OS and PFS. ITS and TMB were adjusted by gender, M stage, LDH, TMB, mutational signatures, and mutations in *BRAF*, *NRAS*, and *NF1* in multivariate logistic and Cox regression models. The logistic and Cox regression model were visualized using “forestplot” R package. The summary predictive effect and between‐cohort heterogeneity were estimated using “meta” R package.^24^ The value of *I* ^2^ was used to evaluate the degree of heterogeneity between the cohorts and the criteria were as follows: low (25%‐50%), moderate (50%‐75%), and high (75%‐100%).^25^

### 2.8 Subgroup and combination analyses

We explored the feasibility and significance of biomarker combination based on ITS, TMB, and LDH for predicting response to ICIs. Considering the correlation between ITS and TMB, we first divided the meta‐cohort into subgroups with high and low TMB according to the median value of TMB. Then we evaluated the association of ITS with DCB, OS, and PFS in high and low TMB subgroups, respectively. In addition, considering the negligible association between ITS and LDH, we combined ITS and LDH as a composite biomarker for predicting DCB, OS, and PFS in melanoma patients treated with ICIs. The overall distribution of ITS, TMB, LDH, and clinical benefit in the meta‐cohort was visualized by “ggalluvial” R package.

### 2.9 Genomic analysis associated with response to ICIs

In this study, we defined TMB as (nonsynonymous mutation counts)/(the whole length of exons). The correlation analysis of ITS with TMB and NAL was performed and visualized using “ggplot2” R package. The correlation degrees were defined as follows: negligible (0.00‐0.10), weak (0.10‐0.39), 0.40‐0.69 (moderate), 0.70‐0.89 (strong), and very strong (0.90‐1.00).^26^ We used “deconstructSigs” R package to extract mutational signatures from the WES data. The deconstructSigs approach determined the linear combination of predefined signatures which accurately reconstruct the mutational profile by establishing a multiple linear regression model.^27^ In this study, Alexandrov signatures were taken as predefined signatures.^28^ Melanoma‐related signatures mainly included signature 1 (age), 7 (ultraviolet exposure) and 11 (pretreated alkylating agent).^28^ The value of signature 1, 7, and 11 was converted into percentage in a single tumor sample (Table S4). The lower third was selected as the cutoff of signatures for multivariate logistic and Cox regression analysis. We integrated somatic mutation and CNV data to characterize genomic alterations in several key signaling pathways associated with response to ICIs in the meta‐cohort. The waterfall plot was visualized using “ComplexHeatmap” R package.^29^

### 2.10 Gene‐set enrichment analysis

To further investigate the distinctive signaling pathways associated with ITS, we integrated RNA‐seq data of the Allen cohort, Snyder cohort, and Liu cohort, then performed gene set enrichment analysis (GSEA) based on the java GSEA 3.0 Desktop Application (http://software.broadinstitute.org/gsea) and hallmark genesets downloaded from the Molecular Signatures Database.^30^ The normalized enrichment score (NES) and false discovery rate (FDR) were the primary statistics for examining gene set enrichment results. FDR < 0.05 was considered as statistically significant.

### 2.11 Statistical analysis

Kaplan–Meier analysis, correlation analysis, logistic, and Cox regression analyses were conducted using SPSS software (version 21.0, IBM Corp). The log‐rank test was used to compare Kaplan–Meier curves. The correlation between ranked variables was determined by Spearman rank correlation coefficient. The OR and its 95% CI were calculated by logistic regression analysis. The HR and its 95% CI were calculated by Cox regression analysis. The comparison between continuous variables was dealt with Student's *t* test, whereas the comparison between ranked variables was dealt with the Mann–Whitney test using GraphPad Prism (version 6.01, GraphPad Software). The categorical variables were compared by chi‐squared (*χ* ^2^) test or Fisher's exact test in the appropriate situation. All reported *P* values were two‐tailed and *p* < .05 was considered as statistically significant.

## 3 RESULTS

### 3.1 Clinical characteristics in cohorts

We collected clinical characteristics of total 318 patients diagnosed with melanoma and treated with anti‐CTLA‐4 or anti‐PD1 therapy from previous studies. The Allen cohort,^15^ Snyder cohort,^16^ and Liu cohort^17^ consisted of 110, 64, and 144 patients, respectively. In the Allen cohort, 52.7% (58/110) was at the normal level of LDH, whereas 43.6% (48/110) was elevated. Overall the median TMB was 6.9 mutations per Mb. All of them were treated with ipilimumab. Best objective response to anti‐CTLA‐4 using RECIST v.1.1 criteria included 2.7% (3/110) with CR, 12.7% (14/110) with PR, 10.9% (12/110) with SD, and 69.1% (76/110) with PD. The median OS was 9.1 months and the median PFS was 2.8 months. Overall, 24.5% (27/110) was with DCB, whereas 70.9% (78/110) was with NCB. In the Snyder cohort, 51.6% (33/64) was at normal level of LDH, whereas 20.3% (13/64) was elevated. Overall, the median TMB was 11.9 mutations per Mb. 93.8% (60/64) was treated with ipilimumab and 6.3% (4/64) was with tremelimumab. The median OS was 25.3 months. Data of best objective response and PFS were not available from the Snyder cohort. 42.2% (27/64) was with DCB, whereas 57.8% (37/64) was with NCB. In the Liu cohort, 48.6% (70/144) was at normal level of LDH, whereas 49.3% (71/144) was elevated. The median TMB was 6.5 mutations per Mb. 41.0% (59/144) was treated with nivolumab and 59.0% (85/144) was with pembrolizumab. Best objective response included 11.8% (17/144) with CR, 26.4% (38/144) with PR, 13.9% (20/144) with SD, 45.1% (65/144) with PD, and 2.8% (4/144) with mixed response (MR). The median OS was 19.4 months and the median PFS was 5.5 months. Overall, 50.0% (72/144) was with DCB and 47.2% (68/144) was with NCB. Detailed clinical characteristics are shown in Table 1.

**TABLE 1:** Clinical characteristics of patients treated with ICIs in three cohorts

Characteristic	Allen cohort	Snyder cohort	Liu cohort
	(N = 110)	(N = 64)	(N = 144)
Age—y			
Median	61.5	62.5	NA
Range	18‐86	18‐90	NA
Gender—no. (%)			
Female	32 (29.1)	25 (39.1)	60 (41.7)
Male	78 (70.9)	39 (60.9)	84 (58.3)
Primary melanoma—no. (%)			
Cutaneous	92 (83.6)	44 (68.8)	105 (72.9)
Mucosal	4 (3.6)	0 (0.0)	10 (6.9)
Acral	0 (0.0)	5 (7.8)	10 (6.9)
Occult	14 (12.7)	7 (10.9)	19 (13.2)
NA	0 (0.0)	8 (12.5)	0 (0.0)
Stage—no. (%)			
M0	10 (9.1)	3 (4.7)	10 (6.9)
M1	100 (90.9)	61 (95.3)	134 (93.1)
M1a	6 (5.5)	NA	8 (5.6)
M1b	16 (14.5)	NA	18 (12.5)
M1b	78 (70.9)	NA	108 (75.0)
LDH—no. (%)			
Normal	58 (52.7)	33 (51.6)	70 (48.6)
Elevated	48 (43.6)	13 (20.3)	71 (49.3)
NA	4 (3.6)	18 (28.1)	3 (2.1)
TMB—muts/Mb			
Median	6.9	11.9	6.5
Range	0.4‐188.7	0.11‐97.8	0.3‐255.5
Drug received—no. (%)			
Ipilimumab	110 (100.0)	60 (93.8)	0 (0.0)
Tremelimumab	0 (0.0)	4 (6.3)	0 (0.0)
Nivolumab	0 (0.0)	0 (0.0)	59 (41.0)
Pembrolizumab	0 (0.0)	0 (0.0)	85 (59.0)
Best response—no. (%)			
Complete response	3 (2.7)	0 (0.0)	17 (11.8)
Partial response	14 (12.7)	0 (0.0)	38 (26.4)
Stable disease	12 (10.9)	0 (0.0)	20 (13.9)
Progressive disease	76 (69.1)	0 (0.0)	65 (45.1)
Mixed response	0 (0.0)	0 (0.0)	4 (2.8)
Not available	5 (4.5)	64 (100.0)	0 (0.0)
Overall survival—mos			
Median	9.1	25.3	19.4
Range	1.1‐54.4	2.5‐94.6	1.3‐56.4
Progression‐free survival—mos			
Median	2.8	NA	5.5
Range	0.5‐49.6	NA	0.4‐56.0
Durable Clinical Benefit			
Yes	27(24.5)	27(42.2)	72(50.0)
No	78(70.9)	37(57.8)	68(47.2)
Not available	5(4.5)	0(0.0)	4(2.8)

Abbreviations: ICIs, immune checkpoint inhibitors; LDH, Lactate dehydrogenase; Mb, megabase; mos, months; muts, mutations; yrs, years.

### 3.2 Construction of genetic mutation model for predicting durable clinical benefit from ICIs therapy in melanoma.

Firstly, based on the WES data of the Allen cohort, we screened out the 425 frequently mutated genes (mutation rate ≥10%). Subsequently, we evaluated the association between the frequently mutated genes and DCB of melanoma patients receiving ICIs therapy by conducting *χ* ^2^ test (Figure 1A). As shown in Table S5, 25 frequently mutated genes were significantly associated with DCB, then identified as candidate genes for multivariate logistic regression analysis. Considering the low applicability in clinical practice for a complex model with 25 variables, we adopted the Backward Elimination (Wald) strategy to control the number of the variable in the multivariate logistic regression model. As a result, four frequently mutated genes (*THSD7B*, *SYNE2*, *GRM3*, and *FLNC*) were included in the model after running 24 steps (Table S6). Then, we obtained the coefficient of each gene from the model and calculated the immunotherapy score (ITS) formula as follows: ITS = 1.234 × *THSD7B* + 1.640 × *SYNE2* + 0.948 × *GRM3* + 1.636 × *FLNC* (Figure 1B, Table S6). In the Allen cohort, 22% (24/110) had *THSD7B* mutations, 20% (22/110) had *GRM3* mutations, 11% (12/110) had *SYNE2* mutations, and 10% (11/110) had *FLNC* mutations (Figure 1C). Overall, 54.5% (60/110) was with low ITS, whereas 45.5% (50/110) was with high ITS. In the Snyder cohort, 31% (20/64) had *THSD7B* mutations, 25% (16/64) had *FLNC* mutations, 14% (9/64) had *SYNE2* mutations, and 14% (9/64) had *GRM3* mutations (Figure 1C). Overall, 43.8% (28/64) was with low ITS, whereas 56.2% (36/64) was with high ITS. In the Liu cohort, 26% (38/144) had *THSD7B* mutations, 15% (22/144) had *SYNE2* mutations, 15% (21/144) had *FLNC* mutations, and 12% (17/144) had *GRM3* mutations (Figure 1C). Overall, 56.3% (81/144) was with low ITS group, whereas 43.7% (63/144) was with high ITS.

### 3.3 Prediction of durable clinical benefit from ICIs therapy by ITS and TMB

**FIGURE 2:**
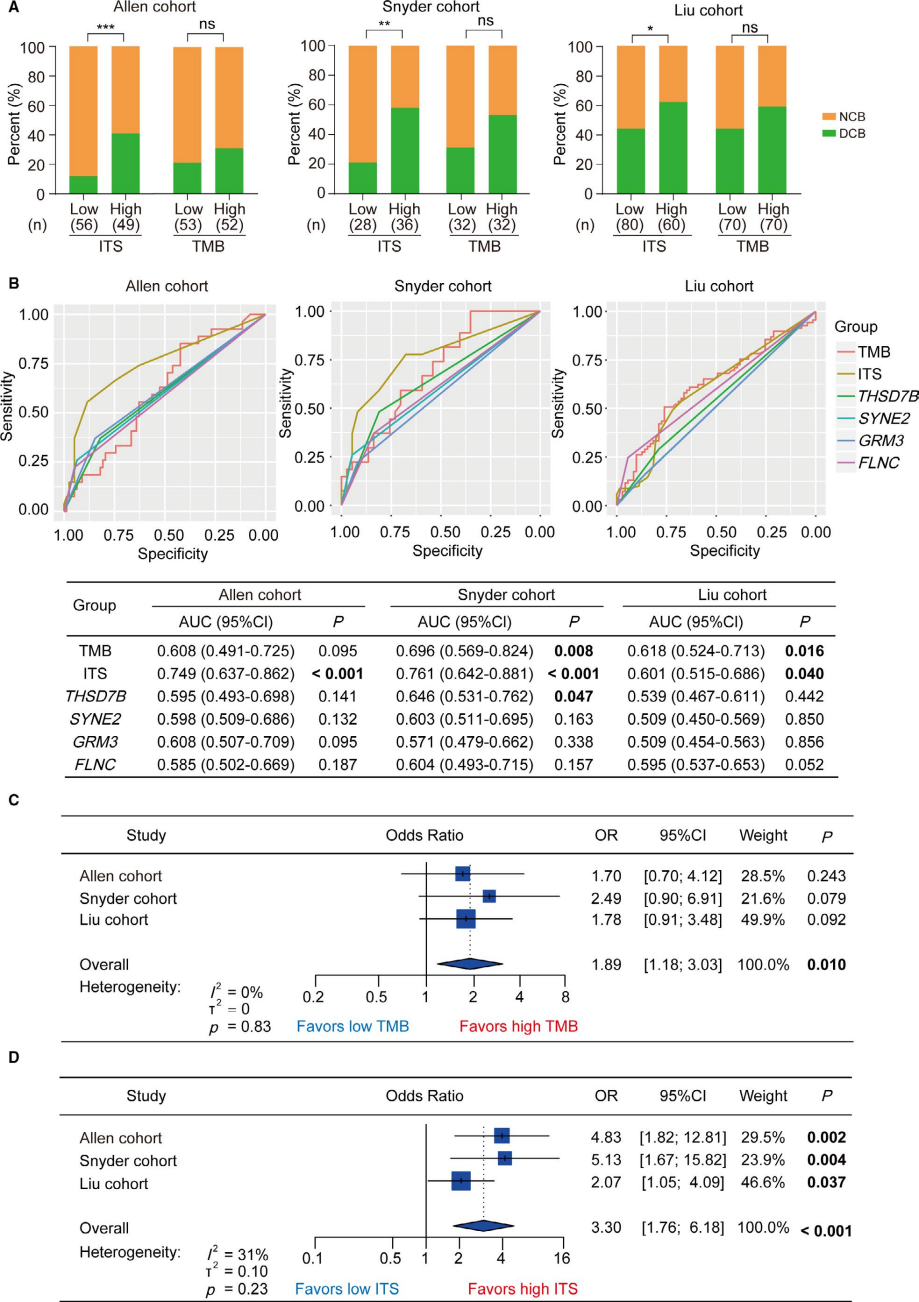
The prediction of durable clinical benefit from ICIs therapy by ITS and TMB. (A) Clinical benefit from ICIs therapy stratified by ITS and TMB in the Allen cohort, Snyder cohort and Liu cohort. (B) ROC curve analysis for prediction of durable clinical benefit from ICIs therapy by ITS, TMB, *THSD7B*, *SYNE2*, *GRM3*, and *FLNC* in the Allen cohort, Snyder cohort, and Liu cohort, respectively. *P* value was calculated by the comparison between tested AUC and reference AUC (equal to 0.5). (C) Forest plot showing univariate logistic regression and meta‐analysis for durable clinical benefit, taking TMB as the input variable in the Allen cohort, Snyder cohort, and Liu cohort. (D) Forest plot showing univariate logistic regression and meta‐analysis for durable clinical benefit, taking ITS as the input variable in the Allen cohort, Snyder cohort, and Liu cohort. ICIs, immune checkpoint inhibitors; ITS, immunotherapy score; TMB, tumor mutation burden; DCB, durable clinical benefit; NCB, no clinical benefit; ROC, receiver operator characteristic; AUC, area under curve; CI, confidence interval; OR, odds ratio; ***p < .001, **p < .01, *p < .05; ns, no significance

ITS was established based on the 4‐gene mutations as mentioned previously. Interestingly, TMB, a promising biomarker of ICIs therapy by clinical evidences,^3^, ^4^, ^5^ was also derived from tumor somatic mutation. Thus, we further analyzed the association between TMB and DCB from ICIs therapy and compared the prediction accuracy between TMB and ITS. First, we evaluated the impact of ITS and TMB on DCB by *χ* ^2^ test, respectively. As a result, the proportions of DCB were all significantly higher in high ITS than low ITS group (Allen cohort, *p* < .001; Snyder cohort, *p* < .01; Liu cohort, *p* < .05; Figure 2A). However, the difference in DCB between high and low TMB was not significant (Allen cohort, *p* = .271; Snyder cohort, *p = *.128; Liu cohort, *p* = .128; Figure 2A). Then we performed ROC analysis and calculated AUC to evaluated the prediction accuracy of DCB from ICIs therapy by single gene, ITS, and TMB. As shown in Figure 2B, according to the corresponding AUCs in three cohorts, TMB was a better biomarker than each single gene for prediction. However, the AUC of ITS was higher than that of TMB in the Allen cohort and Snyder cohort (0.749 vs. 0.608; 0.761 vs. 0.696; Figure 2B). In the Liu cohort, the AUCs of ITS were slightly less than the AUC of TMB (0.601 vs. 0.618; Figure 2B). Furthermore, we performed univariate logistic regression analysis for DCB to calculate the ORs stratified by TMB and ITS, respectively. Subsequently, we evaluated the summary predictive effect and between‐cohort heterogeneity by meta‐analysis. It was found that patients with high TMB tended to take large ratio of DCB, but the difference was not significant in the individual cohort (Allen cohort: OR = 1.70, 95%CI = [0.70,4.12], *p* = 0.243; Snyder cohort: OR = 2.49, 95%CI = [0.90,6.91], *p* = .079; Liu cohort: OR = 1.78, 95%CI = [0.91,3.48], *p* = .092; Figure 2C). In the meta‐cohort, TMB was a significant predictor of DCB (OR = 1.89, 95%CI = [1.18,3.03], *p* = .010; heterogeneity: *I* ^2^ = 0, *p* = .83; Figure 2C). Remarkably, ITS was capable of predicting DCB significantly in the independent cohorts (Allen cohort: OR = 4.83, 95%CI = [1.82,12.81], *p* = .002; Snyder cohort: OR = 5.13, 95%CI = [1.67,15.82], *p* = .004; Liu cohort: OR = 2.07, 95%CI = [1.05,4.09], *p* = .037; Figure 2D). Meta‐analysis further illustrated that the prediction of ITS for DCB was robust. (OR = 3.30, 95%CI = [1.76,6.18], *p* < .001; heterogeneity: *I* ^2^ = 31%, *p* = .23; Figure 2D).

### 3.4 Prognostic impact of TMB and ITS on overall survival in melanoma patients treated with ICIs

**FIGURE 3:**
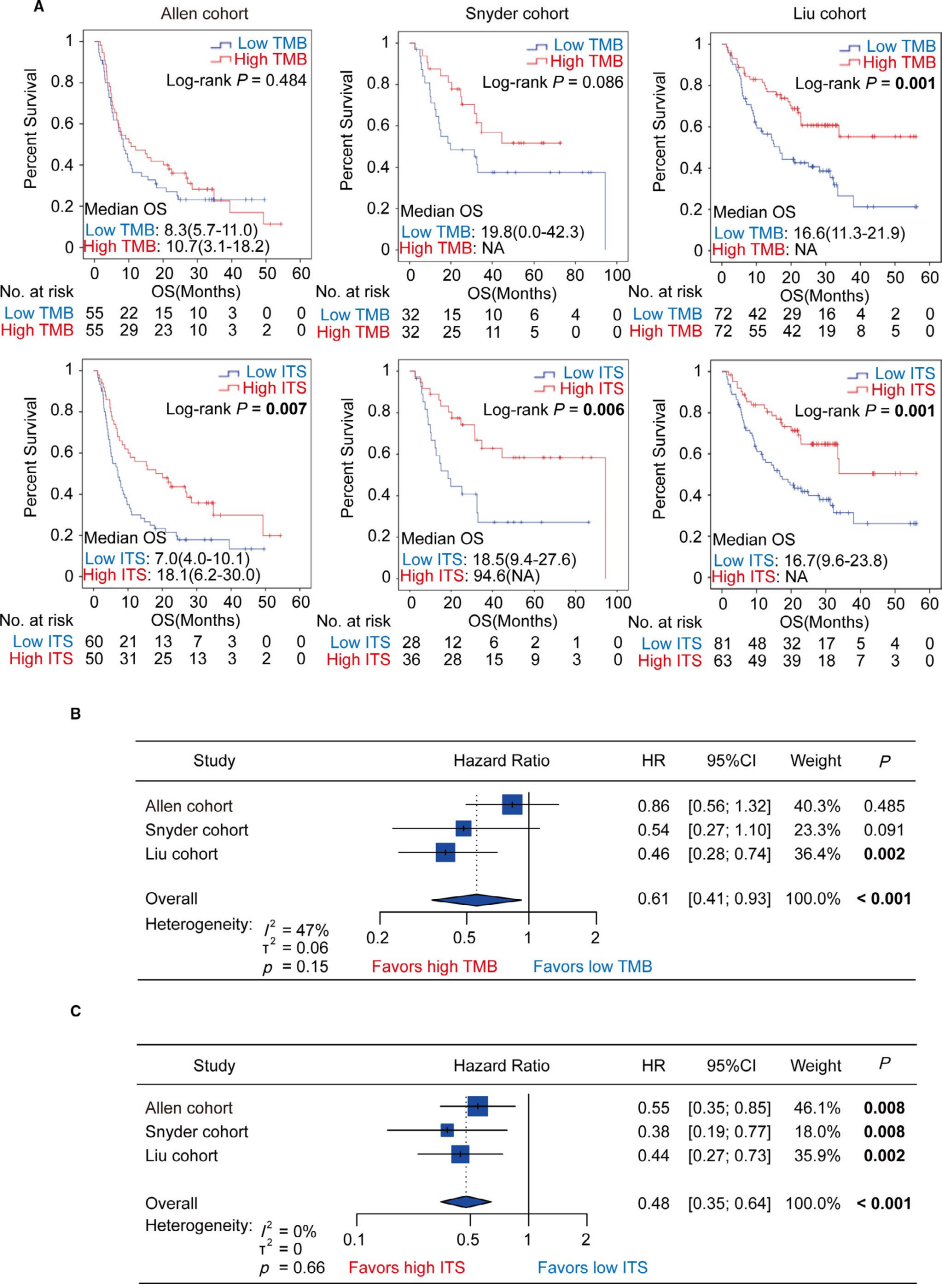
Kaplan–Meier analysis for overall survival stratified by TMB and ITS in the Allen cohort, Snyder cohort, and Liu cohort. (A) The Kaplan–Meier curves for overall survival stratified by TMB and ITS in the Allen cohort, Snyder cohort, and Liu cohort, respectively. (B) Forest plot showing univariate Cox regression and meta‐analysis for overall survival taking TMB as the input variable in the Allen cohort, Snyder cohort, and Liu cohort. (C) Forest plot showing univariate Cox regression and meta‐analysis for overall survival taking ITS as the input variable in the Allen cohort, Snyder cohort, and Liu cohort. TMB, tumor mutation burden; ITS, immunotherapy score; OS, overall survival; HR, hazard ratio; CI, confidence interval

We performed Kaplan–Meier analysis to evaluate the impact of TMB and ITS on overall survival (OS). We found that the impact of TMB on OS was inconsistent when stratified by high and low TMB in three independent cohorts. High TMB group was significantly associated with better OS in the Liu cohort (log‐rank *p* = .001; Figure 3A) but the similar impact was not observed in the Allen cohort and Snyder cohort (Allen cohort: log‐rank *p* = .484; Snyder cohort: log‐rank *p* = .086; Figure 3A). However, compared with low ITS group, high ITS group dramatically improved OS, which was consistently significant in the cohorts (Allen cohort: log‐rank *p* = .007; Snyder cohort: log‐rank *p* = .006; Liu cohort: log‐rank *p* = .001; Figure 3A). Univariate Cox regression analysis revealed that patients with high TMB presented a tendency toward better OS in the cohorts, which was inconsistent in the cohorts (Allen cohort: HR = 0.86, 95%CI = [0.56,1.32], *p* = .485; Snyder cohort: HR = 0.54, 95%CI = [0.27,1.10], *p* = .091; Liu cohort: HR = 0.46, 95%CI = [0.28,0.74], *p* = .002; Figure 3B). Meta‐analysis showed that TMB was significantly associated with overall survival in meta‐cohort, but the heterogeneity was close to moderate degree (HR = 0.61, 95%CI = [0.41,0.93], *p* < .001; heterogeneity: *I* ^2^ = 47%, *p* = .15; Figure 3C). Compared with TMB, ITS was a more robust predictor of OS in melanoma patients treated with ICIs (Allen cohort: HR = 0.55, 95%CI = [0.35,0.85], *p* = .008; Snyder cohort: HR = 0.38, 95%CI = [0.19,0.77], *p* = .008; Liu cohort: HR = 0.44, 95%CI = [0.27,0.73], *p* = .002; Figure 3B). Meta‐analysis also showed that ITS was significantly associated with overall survival (HR = 0.48, 95%CI = [0.35,0.64], *p* < .001; heterogeneity: *I* ^2^ = 0%, *p* = .66; Figure 3C).

### 3.5 Prognostic impact of TMB and ITS on progression‐free survival in melanoma patients treated with ICIs

**FIGURE 4:**
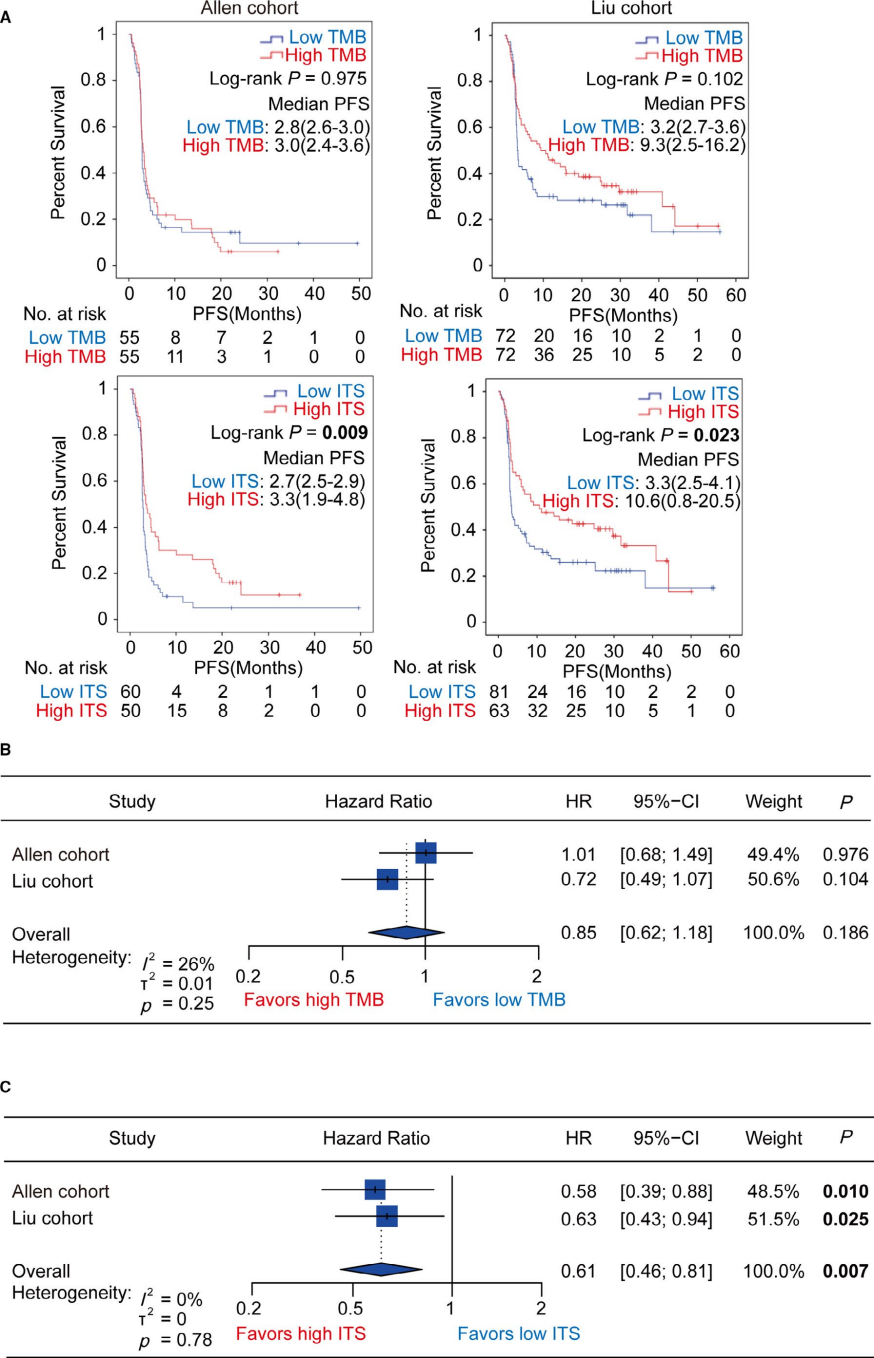
Kaplan–Meier analysis for progression‐free survival stratified by TMB and ITS in the Allen cohort and Liu cohort. (A) The Kaplan–Meier curves for progression‐free survival stratified by TMB and ITS in the Allen cohort and Liu cohort, respectively. (B) Forest plot showing univariate Cox regression and meta‐analysis for progression‐free survival taking TMB as the input variable in the Allen cohort and Liu cohort. (C) Forest plot showing univariate Cox regression and meta‐analysis for progression‐free survival taking ITS as the input variable in the Allen cohort and Liu cohort. TMB, tumor mutation burden; ITS, immunotherapy score; PFS, progression‐free survival; HR, hazard ratio; CI, confidence interval

We further examined the impact of TMB and ITS on PFS in melanoma patients treated with ICIs. Due to lack of data on PFS in the Snyder cohort, we performed the Kaplan–Meier analysis in the Allen cohort and Liu cohort. We observed that the differences in PFS between high and low TMB group were not significant in the cohorts (Allen cohort: log‐rank *p* = .975; Liu cohort: log‐rank *p* = .102; Figure 4A). However, high ITS group showed improved PFS significantly (Allen cohort: log‐rank *p* = .009; Liu cohort: log‐rank *p* = .023; Figure 4A). Univariate Cox regression analysis and meta‐analysis also indicated that ITS was an effective predictor of PFS in the cohorts (Allen cohort: HR = 0.58, 95%CI = [0.39,0.88], *p* = .010; Liu cohort: HR = 0.63, 95%CI = [0.43,0.94], *p* = .025; meta‐analysis: HR = 0.61, 95%CI = [0.46,0.81], *p* = .007; heterogeneity: *I* ^2^ = 0%, *p* = .78; Figure 4C), whereas TMB was not associated with PFS (Allen cohort: HR = 1.01, 95%CI = [0.68,1.49], *p* = .976; Liu cohort: HR = 0.72, 95%CI = [0.49,1.07], *p* = .104; meta‐analysis: HR = 0.85, 95%CI = [0.62,1.18], *p* = .186; heterogeneity: *I* ^2^ = 26%, *p* = .25; Figure 4B).

### 3.6 Univariate and multivariate logistic and Cox regression for DCB, OS, and PFS

**FIGURE 5:**
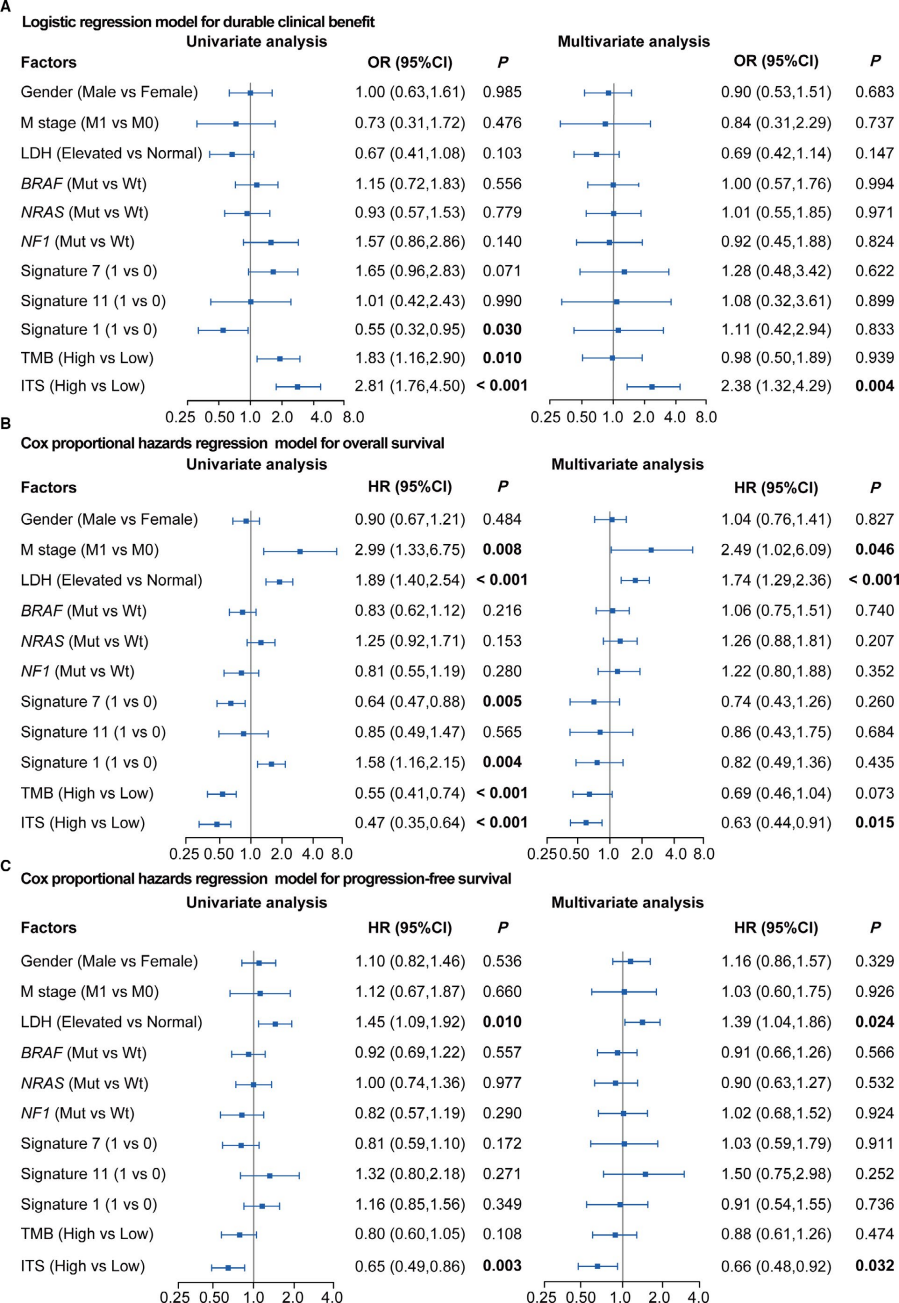
Univariate and multivariate logistic and Cox regression analysis to evaluate the independent effect of ITS on durable clinical benefit and survival outcomes (overall survival and progression‐free survival) in meta‐cohort. (A) Univariate and multivariate logistic regression analysis for durable clinical benefit. (B) Univariate and multivariate Cox proportional hazards regression analysis for overall survival. (C) Univariate and multivariate Cox proportional hazards regression model for progression‐free survival. The value of 1 represented positive, whereas 0 represented negative in Signature. The median value was taken as the cutoff of TMB. Meta‐cohort was composed of the Allen cohort, Snyder cohort, and Liu cohort. Data were adjusted by gender, M stage, LDH, TMB, mutational signatures and mutations in *BRAF*, *NRAS*, and *NF1*. LDH, lactate dehydrogenase; TMB, tumor mutation burden; ITS, immunotherapy score; OR, odds ratio; HR, hazard ratio; CI, confidence interval; Mut, mutation; Wt, wild type

In order to corroborate whether ITS was an independent predictor of DCB, OS, and PFS, we performed the univariate and multivariate logistic regression for DCB and the univariate and multivariate Cox regression for OS and PFS. Data were adjusted by gender, M stage, LDH, TMB, mutations in *BRAF*, *NRAS*, and *NF1*, and mutational signatures. In the univariate logistic regression model, signature 1 was identified as a significantly negative factor (OR = 0.55, *p* = .030; Figure 5A), whereas ITS (OR = 2.81, *p* < .001; Figure 5A) and TMB (OR = 1.83, *p* = .010; Figure 5A) were positive. The multivariate logistic regression analysis revealed that ITS was an independently positive predictor of DCB (OR = 2.38, *p* = .004; Figure 5A), but the prediction of TMB was not independent (OR = 0.98, *p* = .939; Figure 5A). Univariate Cox regression for OS showed that M stage (HR = 2.99, *p* = .008; Figure 5B), LDH (HR = 1.89, *p* < .001; Figure 5B) and signature 1 (HR = 1.58, *p* = .004; Figure 5B) were significant risk factors, whereas signature 7 (HR = 0.64, *p* = .005; Figure 5B), TMB (HR = 0.55, *p* < .001; Figure 5B) and ITS (HR = 0.47, *p* < .001; Figure 5B) were significantly protective factors. The multivariate Cox regression analysis for OS revealed that ITS (HR =0.63, *p* = .015; Figure 5B) was one of the independent indicators for OS, others including M stage (HR = 2.49, *p* = .046; Figure 5B) and LDH (HR = 1.74, *p* < .001; Figure 5B). Univariate Cox regression for PFS showed that LDH (HR = 1.45, *p* = .010; Figure 5C) was identified as significant risk factor, whereas ITS (HR = 0.65, *p* = .003; Figure 5C) was protective for PFS. The multivariate Cox regression analysis revealed that LDH (HR = 1.39, *p* = .024; Figure 5C) and ITS (HR = 0.66, *p* = .032; Figure 5C) were the independent indicators of PFS. TMB was not significantly associated with PFS (HR = 0.80, *p* = .108; Figure 5C) in the univariate Cox regression model. These findings suggested that ITS was a relatively independent biomarker of DCB, OS, and PFS in melanoma patients treated with ICIs.

### 3.7 Biomarker combination based on ITS, TMB, and LDH for predicting response to ICIs

**FIGURE 6:**
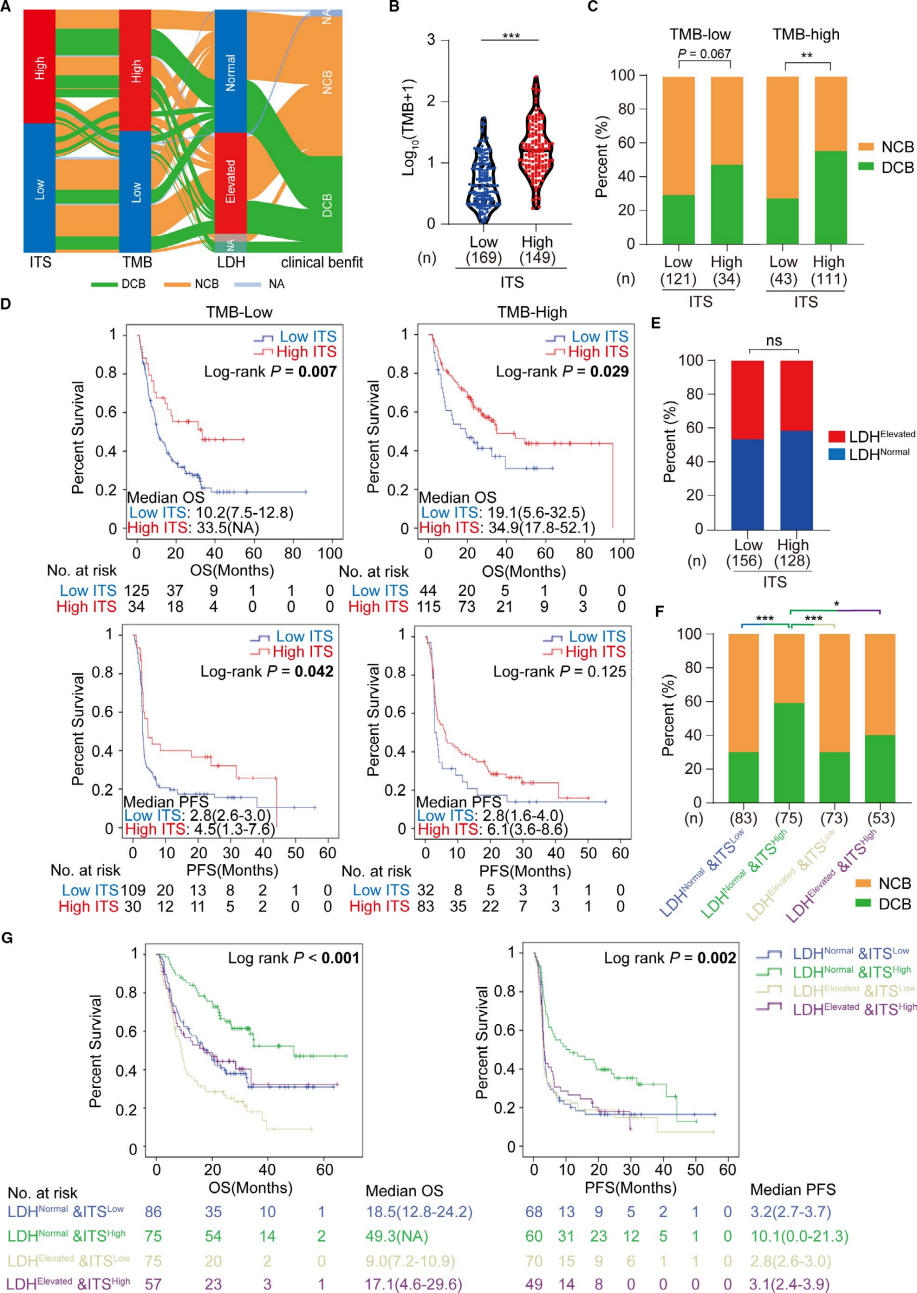
Association between ITS and response to ICIs by subgroup and combination analyses in the meta‐cohort. (A) Sankey diagram showing the overall distribution of ITS, TMB, LDH, and clinical benefit. (B) Comparison of TMB between high and low ITS groups. (C) Comparison of durable clinical benefit between high and low ITS groups stratified by TMB. (D) Kaplan–Meier survival curves of OS and PFS comparing the high and low ITS groups stratified by TMB. (E) Comparison of LDH between high and low ITS groups. (F) Comparison of durable clinical benefit in different subgroups stratified by the combination of ITS and LDH. (G) Kaplan–Meier curves of overall survival and progression‐free survival according to different subgroups of ITS and LDH. Meta‐cohort was composed of the Allen cohort, Snyder cohort, and Liu cohort. ITS, immunotherapy score; TMB, tumor mutation burden; LDH, lactate dehydrogenase; DCB, durable clinical benefit; NCB, no clinical benefit; OS, overall survival; PFS, progression‐free survival; ***p < .001, **p < .01, *p < .05; ns, no significance

We investigated the compatibility of ITS with other biomarkers (TMB and LDH) and the potential of joint prediction for response to ICIs. We observed that a part of patients with low TMB or elevated LDH level harbored DCB, some of whom showed high ITS (Figure 6A). Besides, some patients with high TMB and normal LDH showed NCB, who were in low ITS group (Figure 6A). In order to understand the association of ITS with TMB, LDH, and clinical benefit, we further evaluated the relevance of ITS to TMB and LDH as a predictive biomarker of response to ICIs in melanoma. We observed that the high ITS group harbored significantly higher TMB (*p* < .001; Figure 6B), whereas the association between ITS and LDH was insignificant (*p* = .402; Figure 6E). Subgroup analysis showed that both the median OS and PFS of patients with high ITS were significantly superior to those of patients with low ITS in the TMB‐low subgroup (OS, *p* = .007; PFS, *p* = .042; Figure 6D). High ITS also improved DCB in the TMB‐low subgroup, although a significant P value was not reached (*p* = .067; Figure 6C). In the TMB‐high subgroup, both the DCB and median OS were significantly improved in patients with high ITS (DCB, *p* = .0022; OS, *p* = .029; Figure 6C‐D), whereas the difference in PFS was insignificant (*p* = .125; Figure 6D). Furthermore, lacking of association between ITS and LDH (Figure 6B), plus independent prognostic merits (Figure 5B‐C), indicated that the combination of ITS and LDH might become a better biomarker than ITS or LDH alone. Patients in the meta‐cohort were stratified into four groups by ITS and LDH. Encouragingly, patients with high ITS and normal LDH showed the best DCB (Figure 6F) and the longest median OS and PFS (OS, *p* < .001; PFS, *p* = .002; Figure 6G) in the four subgroups. These findings suggested that the biomarker‐combination strategy of ITS, TMB, and LDH will be laying the foundation for further research in predicting response to ICIs in melanoma.

### 3.8 Distinctive genomic patterns associated with ITS

**FIGURE 7:**
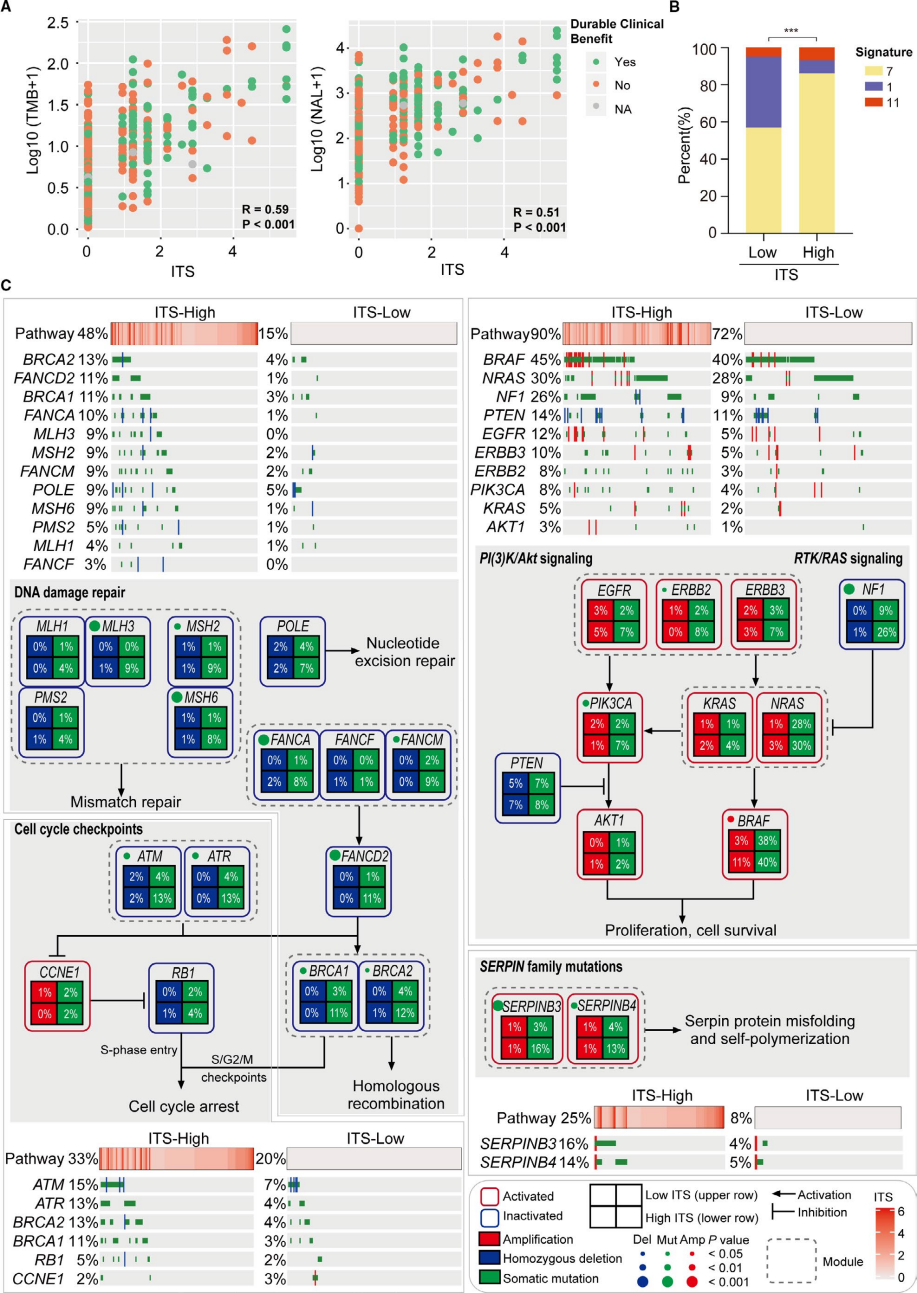
Distinctive genomic patterns associated with ITS. (A) Scatter plots of TMB vs ITS and NAL versus ITS. (B) Proportions of mutational signatures in subgroups with high and low ITS. Signature 7 was mainly associated with ultraviolet exposure. Signature 1 exhibited strong positive correlations with age. Signature 11 was found in melanoma patients treated with the alkylating agent. (C) Genomic alterations in the pathways associated with response to immunotherapy. ITS, immunotherapy score; TMB, tumor mutation burden; NAL, neoantigen load; R, Spearman correlation coefficient. Amp, amplification; Mut, somatic mutation; Del, homozygous deletion; ***p < .001

We characterized the distinctive genomic patterns associated with ITS, based on integrating somatic mutation and CNV data. First, we performed correlation analysis to examine the correlation between TMB and ITS. NAL was also included in the correlation analysis. It was shown that the correlations of ITS with TMB and NAL were at moderate degrees (ITS with TMB: Spearman's R = 0.59, *p* < .001; ITS with NAL: Spearman's R = 0.51, *p* < .001; Figure 7A). Compared with low ITS group, high ITS group had significantly higher TMB (*p* < .001; Figure 6B) and NAL (*p* < .001; Figure S2). The extracted mutational signatures in melanoma including signature 1 (Age), signature 7 (ultraviolet exposure) and signature 11 (pretreated alkylating agent) were significantly distinct between high and low ITS groups (signature 1, 38.2% vs. 7.6%; signature 7, 56.5% vs. 84.8%; signature 11, 5.4% vs. 7.6%; *p* < .001; Figure 7B). Then we characterized several key pathways associated with response to ICIs as previously reported.^31^, ^32^, ^33^, ^34^ As was shown in Figure 7C, high ITS group showed more frequently alterations than low ITS group in DNA damage repair (48% vs. 15%, *p* < .001), cell cycle (33% vs. 20%, *p* < .001), *PI(3)K*/*Akt* and *RTK*/*RAS* pathways (90% vs. 72%, *p* < .001), and *SERPIN* family (25% vs. 8%, *p* < .001). In the DNA damage repair pathway, multiple genes involved in homologous recombination were more frequent mutated in high ITS than low ITS group, including *FANCA* (8% vs. 1%, *p* < .001), *FANCM* (9% vs. 2%, *p* < .01), *FANCD2* (11% vs. 1%, *p* < .001), *BRCA1* (11% vs. 3%, *p* < .01), and *BRCA2* (12% vs. 4%, *p* < .05). The core genes in the mismatch repair pathway were also more frequently altered in high ITS group, including *MLH3* (9% vs. 0%, *p* < .001), *MSH6* (8% vs. 1%, *p* < .001), and *MSH2* (9% vs. 1%, *p* < .01). *ATM* (13% vs. 4%, *p* < .01) and *ATR* (13% vs. 4%, *p* < .01) were significant altered in high ITS group, which were master controllers of cell cycle checkpoint pathways responding to DNA damage. Besides, we observed frequent aberrations in *RTK*/*RAS* signaling and *PI(3)K*/*Akt* signaling, including mutations in *NF1* (26% vs. 9%, *p* < .001), *ERBB2* (8% vs. 2%, *p* < .05), *PI3KCA* (7% vs. 2%, *p* < .01), and amplification in *BRAF* (11% vs. 3%, *p* < .01). Notably, ITS was not associated with deletion of *PTEN* (7% vs. 5%, *p* = .475), a key factor leading to immunotherapy resistance as previously reported.^33^ Moreover, mutations in *SERPINB3* (16% vs. 3%, *p* < .001) and *SERPINB4* (13% vs. 4%, *p* < .01) were more frequent in high ITS than low ITS group, which might promote serpin protein misfolding to increase tumor immunogenicity.^34^

In addition, we performed gene set enrichment analysis (GSEA) on hallmark gene sets based on the RNA‐seq data of the meta‐cohort. Genes involved in inflammatory response pathways were significantly enriched in high ITS group, including TNF‐α Signaling Via NF‐ΚB, Interferon‐γ Response, Allograft Rejection, Interferon‐αResponse, IL‐2/STAT5 Signaling, and IL‐6/JAK/STAT3 Signaling (Figure S3A‐F). Besides, cell cycle pathways including G2M Checkpoint, E2F Targets, Apoptosis, and UV Response Up pathway were also associated with high ITS (Figure S3G‐J). No hallmark pathway was significantly enriched in low ITS group.

## 4 DISCUSSION

Despite a number of studies have shown the powerfully predictive capability of TMB on response to ICIs,^3^, ^4^, ^5^ however, the limitations of TMB may restrict the clinical application. For example, the best TMB threshold is still a mystery so that different studies adopt diverse cutoff values.^5^, ^35^, ^36^ Besides, WES takes intensive time and huge cost.^9^ In order to avoid these limitations, we developed a novel genetic mutation model named as immunotherapy score (ITS) for predicting response to ICIs in melanoma. Besides, we compared the prediction capabilities of TMB and ITS for response to ICIs in melanoma.

Most studies supported TMB as a promising predictor of clinical benefit and survival in immunotherapy.^3^, ^5^, ^37^ However, Morrison et al reported that TMB had no statistically significant impact on OS in melanoma patients.^38^ The debatable association with OS and PFS might induce doubts on the forecasting value of TMB identified as an independent biomarker in clinical practice.^38^ Our findings demonstrated that when taking median or upper quartile (Figure S4) as the cut‐off value, TMB both failed to consistently predict DCB, OS and PFS in different cohorts. However, ITS was identified as a more robust biomarker of response to ICIs in melanoma. To our knowledge, in the field of melanoma, it is firstly reported that the 4‐gene mutation model is associated with clinical benefit and survival outcomes from immunotherapy. Notably, we did not observe the significant association between ITS and survival outcomes of patients without ICIs therapy in TCGA‐SKCM and ICGC‐MELA cohort (Figure S5), suggesting that ITS was a predictor of response to ICIs instead of a prognostic biomarker. Encouragingly, ITS is only dependent on the mutational status of *THSD7B*, *SYNE2*, *GRM3*, and *FLNC*, making it more convenient to identify the cut‐off value than TMB. Moreover, a customized targeted sequencing panel containing *THSD7B*, *SYNE2*, *GRM3*, and *FLNC* can be designed, and the targeted next‐generation sequencing (NGS) can be conducted to determine ITS accurately, which is less‐cost and more convenient than WES.^9^ Similarly, a previous study constructed a 24‐gene mutation model for predicting cancer immunotherapy response and recommended the NGS gene panel as the testing method in clinical practice.^39^ Although TMB could also be estimated by NGS, hundreds of genes should be employed in the panel,^40^ which may take higher cost and longer time than the four‐gene panel in clinical tests. Importantly, biomarker selection for ICIs in clinical practice is diverse and personalized. Multibiomarker predictive system may be able to better capture the likelihood of response to ICIs than any single biomarker.^12^ Liu et al reported that the combination of TMB and CNV stratified predictive response to ICIs across metastatis cancer.^41^ Cristescu et al assessed the potential for a T‐cell–inflamed gene expression profile and TMB to jointly predict response to ICIs in solid tumors.^42^ Cona et al found that the combination of baseline LDH serum level, performance status, and age provided better prediction of response to ICIs in solid tumors compared with LDH alone.^43^ In this study, we also evaluated the potential for combining ITS with TMB or LDH to jointly predict response to ICIs in melanoma. Interestingly, our findings indicated that a specific population with high ITS might benefit from ICIs therapy even if TMB was low or LDH was elevated. Our study suggested that the combination of ITS with other biomarkers may produce synergic and complementary effects on ICIs efficacy in clinical practice. Moreover, considering the frequent alterations of *THSD7B*, *SYNE2*, *GRM3*, and *FLNC* in lung squamous cell carcinoma and lung adenocarcinoma (Figure S6), we investigated the applicability of ITS to NSCLC patients treated with ICIs. Encouragingly, both DCB and PFS in high ITS group were significantly improved in nonsquamous type of NSCLC patients. However, the difference was insignificant in squamous type of NSCLC patients (Figure S7). Whatever, the applicability of ITS for predicting response to ICIs in NSCLC needs to be further validated in some independent cohorts.

One of the reasons that melanoma is considered to be immunologically active is the high TMB associated with ultraviolet light damage.^12^ A leading explanation is that high TMB increases the formation and presentation of immunological neoantigen to induce an effective anti‐tumor immune response.^44^ Interestingly, we observed that patients with high ITS harbored abundant signature 7 (ultraviolet light damage) (Figure 7B) and presented significantly increased TMB and NAL (Figure S2). It was suggested that melanoma with high ITS showed genomic instability, which might influence genomic signaling pathways associated with immunotherapy sensitivity and resistance. Numerous genomic biomarkers correlated with response to ICIs have been reported over the past years. Several lines of evidence suggested that DNA damage repair represented important biomarkers of ICIs therapy, including comutations in homologous recombination repair and mismatch repair,^31^ mutations in *BRCA2* ^45^ and *POLE*.^46^ In addition, *RAS*/*MAPK* pathway was associated with immunotherapy in melanoma. Patients with *NRAS* or *NF1* mutations had high response rates.^9^, ^14^ Another mutational event associated with response to ICIs in melanoma were mutations in *SERPINB3* and *SERPINB4*, which might promote the formation of immunologically significant neoepitopes.^34^ Besides, Peng et al observed that loss of *PTEN* promoted resistance to T‐cell‐mediated immunotherapy by increasing activation of the *PI3K*‐*AKT* pathway.^33^ In this study, we found that patients with high ITS had a wide range of mutations in DNA damage repair and cell cycle pathway (Figure 7C), including homologous recombination repair (*BRCA1*/*2*, *FANCD*, *FANCA*, and *FANCM*), mismatch repair (*MSH6*, *MLH3*, and *MSH2*), and cell cycle checkpoints (*ATM* and *ATR*). More frequent mutations in *NF1*, *SERPINB3*, and *SERPINB4* were also observed in the high ITS group compared to low ITS group. However, we did not find the association between ITS and loss of *PTEN* or mutations in *NRAS*. Moreover, GSEA analysis revealed that tumors with high ITS showed active inflammatory response and impaired cell cycle, which were favorable for ICIs therapy. These findings could partly explain why ITS could serve as an independently genetic biomarker of ICIs therapy in melanoma. However, the biological mechanisms how the ITS‐related genes (*THSD7B*, *SYNE2*, *GRM3*, and *FLNC*) impact ICIs response are still unclear at present. In order to preliminarily establish a biologic hypothesis, we performed a literature review and investigated the biological functions of these genes from the previous studies (Table S7). Lüke et al reported that *SYNE2* giant maintained nuclear envelope architecture and composition in skin.^47^ Warren et al reported that *SYNE2*‐dependent pathway regulated the DNA damage response in vascular smooth muscle cell aging.^48^ Thus, we assumed that nonsynonymous mutation in *SYNE2* may impair the DNA damage response pathway and increase TMB. Interestingly, Krauthammer et al identified *GRM3* as one of the genes with a high mutation burden in sun‐exposed melanomas.^49^ It may be one of the reasons that signature 7 (ultraviolet exposure) is enriched in the high ITS group in our study. Besides, Qian et al found that blood‐based mutation of *GRM3* was associated with response to immunotherapy in NSCLC.^50^ Combining these previous findings with our results, we considered that mutations in *GRM3* may also play a key promotor in high TMB to increase the potential of response to ICIs in melanoma. However, the mechanism of how *GRM3* takes impact on TMB is still unclear at present. As for *THSD7B* and *FLNC*, it has been reported that they are associated with tumor progression and/or prognosis of multiple tumors, such as NSCLC,^51^ gastric cancer,^52^ and hepatocellular carcinoma,^53^ where the mechanism is still a mystery. It is worth exploring other potential mechanisms of how these genes impact immunotherapy. Whatever, we consider that the biological mechanism of how *THSD7B*, *SYNE2*, *GRM3*, and *FLNC* take impact on the immunotherapy requires more evidences in vitro/in vivo. Understanding the role of these genes in immunotherapy will be an important area of cancer research in the future.

The identification of predictive biomarkers for ICIs therapy has become a hot spot of intense research. For example, a previous study identified the Tumor Immune Dysfunction and Exclusion (TIDE) as a reliable ICIs biomarker and developed a web application for calculating the TIDE of transcriptional samples.^54^ In this study, we compared the difference in predictive power between ITS and TIDE. As a result, the predictive power for clinical benefit of the TIDE was weaker than that of ITS in the cohorts (AUC: 0.693 vs. 0.749 in the Allen cohort; 0.558 vs. 0.761 in the Snyder cohort; 0.554 vs. 0.601 in the Liu cohort; Figure S8A, Figure 2). Survival analysis revealed that patients with high TIDE harbored poor survival outcomes in the Allen cohort (OS: HR = 2.80, *p* = .006; PFS: HR = 4.14, *p* < .001; Figure S8B‐C). However, the association of the TIDE with survival outcomes was not significant in the Snyder cohort (OS: HR = 1.02, *p* = .006; PFS: not available; Figure S8B) and the Liu cohort (OS: HR = 1.06, *p* = .885; PFS: HR = 1.00, *p* = .999; Figure S8B‐C). It was indicated that the selection bias of the validating cohorts may impact the prediction power of the TIDE in this study. Besides, according to the cut‐off value of TIDE in the original article,^54^ patients in the Liu cohort were divided into two groups with hugely different numbers (105 cases in low group vs. 16 cases in high group), which may bring the bias in this study (Figure S8B‐C). However, in the same cohorts as mentioned before, melanoma patients with high ITS consistently harbored better treatment outcomes (DCB, OS, and PFS) from ICIs therapy. These findings suggested that the predictive power of ITS was more effective and robust than that of TIDE in this study, which required more validations in other clinical cohorts.

It was worth noting that our study had several limitations. First, WES data from the different cohorts were based on the different platforms lacking of uniformed criteria, which may limit the definition of the cut‐off value of TMB, therefore we adopted the median and upper quartile value rather than a specific value as the threshold to decrease the bias on TMB from the different cohorts. Second, some data such as PFS were not available in the specific cohort obtained from recent publications, making the analysis incomplete in part of this study. Third, we did not differentiate whether the genes (*THSD7B*, *SYNE2*, *GRM3*, and *FLNC*) mutations were functional. In other words, we did not explore the mechanism of how mutations in these genes made impact on TMB, NAL, key pathways as mentioned, and other factors associated with ICIs therapy. In addition, this retrospective study may be affected by potential confounding factors, such as the selection bias of the cohorts. It would be ideal if other independent cohorts support our investigation. Whatever the genetic mutation model requires validation in prospective clinical cohorts.

In conclusion, this study provided evidences that the genetic mutation model identified a melanoma population with multiple genetic patterns of sensitivity to ICIs, who might potentially benefit from ICIs therapy. Preliminary data from three independent cohorts strongly suggested better treatment outcomes from ICIs therapy in melanoma patients with high ITS. Remarkably, the combination strategy of ITS and TMB or LDH showed better prediction efficacy compared with any single biomarker. These findings deserve prospective investigation in the future and may help guide clinical decisions on ICIs therapy for patients with melanoma.

## CONFLICTS OF INTEREST

The authors declare that they have no conflict of interests.

## AUTHORS’ CONTRIBUTIONS

Lisong Teng conceived and designed the study. Junjie Jiang and Yongfeng Ding collected data, performed data analysis, and wrote manuscript. Junjie Jiang, Mengjie Wu, and Yanyan Chen drew figures. Xiadong Lyu performed literature search. Jun Lu and Haiyong Wang were involved in data interpretation and critically reviewing the manuscript. All authors read and approved the final manuscript.

## ETHICAL STATEMENT

The authors declare human ethics approval was not needed for this study.

## Supporting information

Fig S1

Fig S2

Fig S3

Fig S4

Fig S5

Fig S6

Fig S7

Fig S8

Table S1

Table S2

Table S3

Table S4

Table S5

Table S6

Table S7

Supplementary Material

## Data Availability

The clinical information, WES, and RNA‐seq data of clinical cohorts (the Allen cohort, Snyder cohort, TCGA‐SKCM cohort, and Hellmann cohort) were downloaded from the cBioPortal database (https://www.cbioportal.org/). Data of the Liu cohort and Miao cohort were acquired from supplemental materials of the reported articles (https://doi.org/10.1038/s41588‐018‐0200‐2; https://doi.org/10.1038/s41588‐018‐0200‐2). The clinical information and WES data of the ICGC‐MELA cohort were downloaded from the International Cancer Genome Consortium (https://icgc.org/).
