# Supplementary figures and images for: Integrated genomic analysis identifies a genetic mutation model predicting response to immune checkpoint inhibitors in melanoma
Source: Cancer Med. 2020 Sep 24;9(22):8498–518. doi: 10.1002/cam4.3481 (PMC7666739; doi:10.1002/cam4.3481)

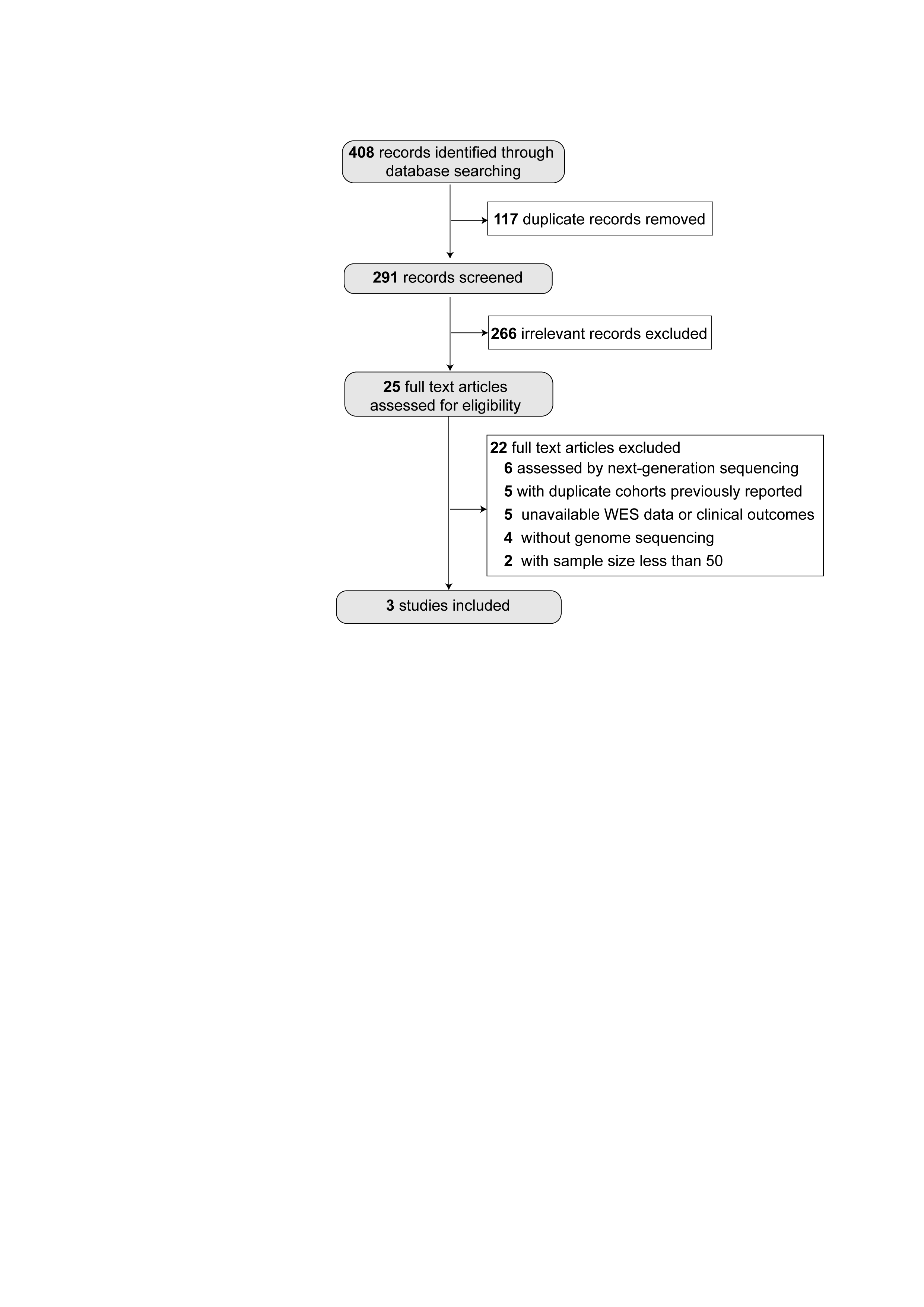

Supplement: Supplementary file 1 — Fig S1 [file CAM4-9-8498-s001.tif]

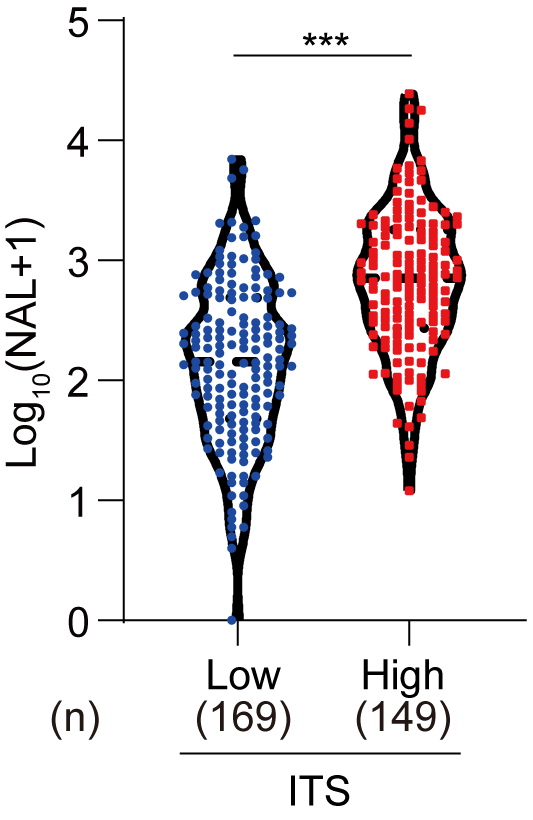

Supplement: Supplementary file 2 — Fig S2 [file CAM4-9-8498-s002.tif]

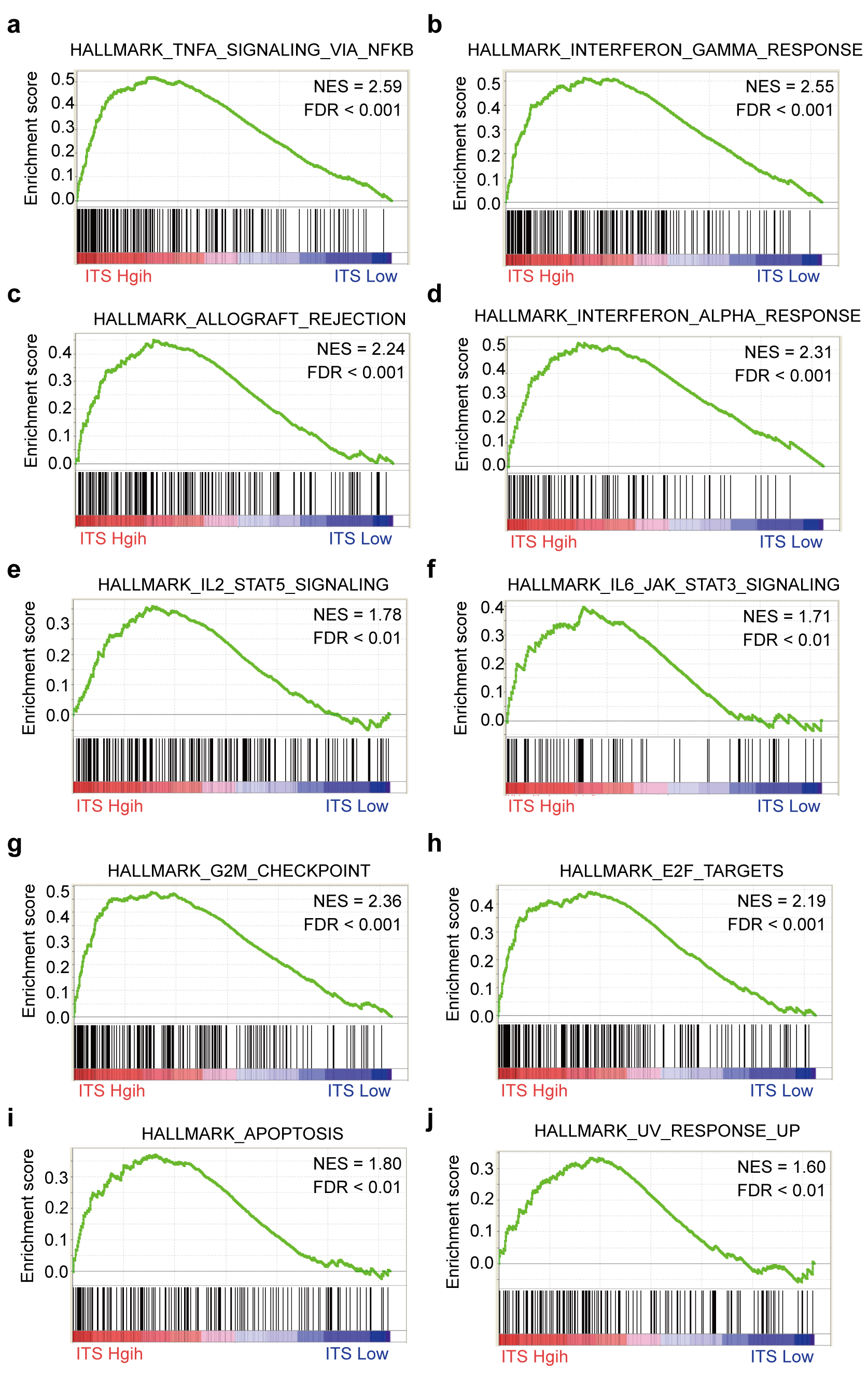

Supplement: Supplementary file 3 — Fig S3 [file CAM4-9-8498-s003.tif]

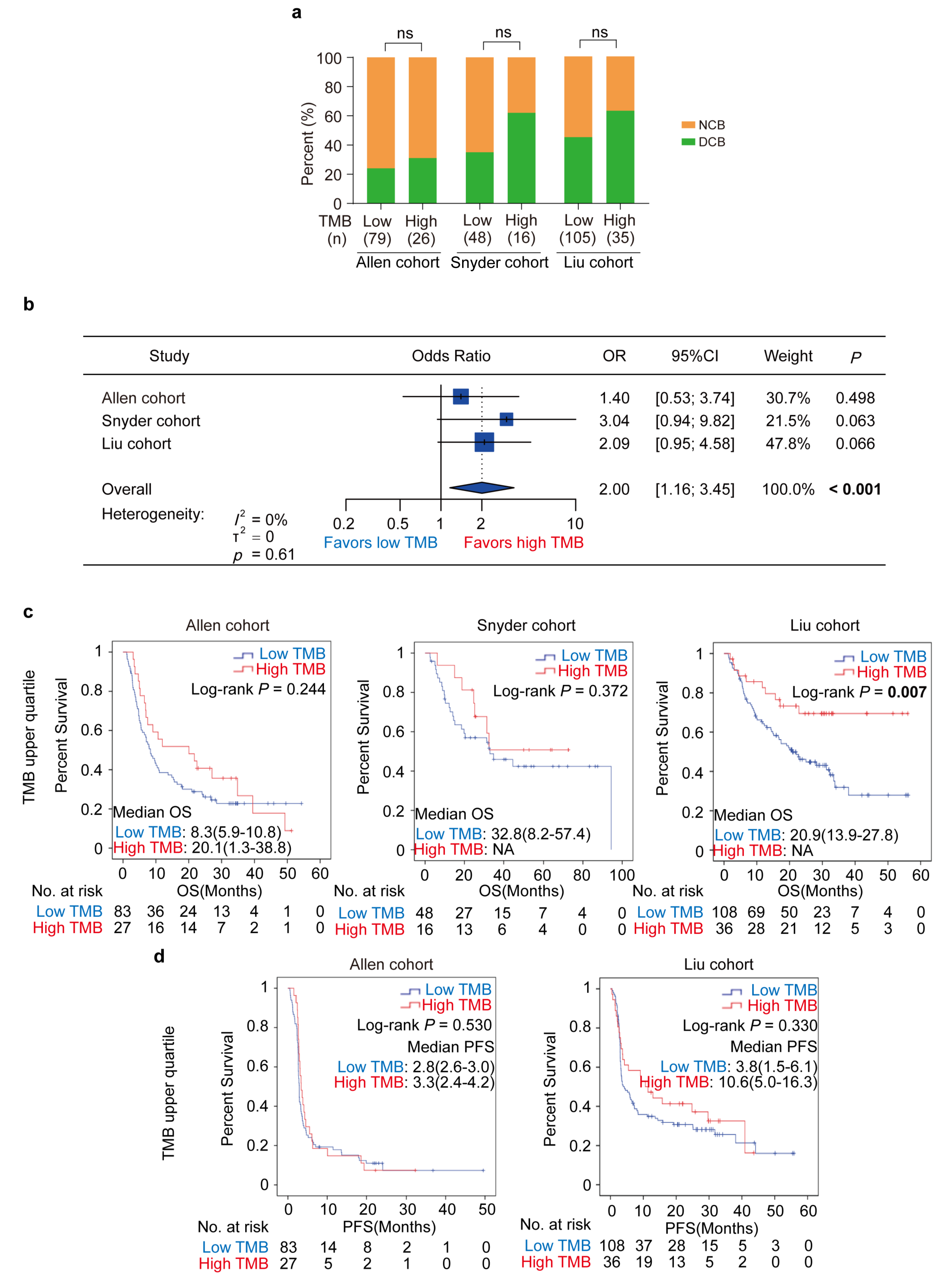

Supplement: Supplementary file 4 — Fig S4 [file CAM4-9-8498-s004.tif]

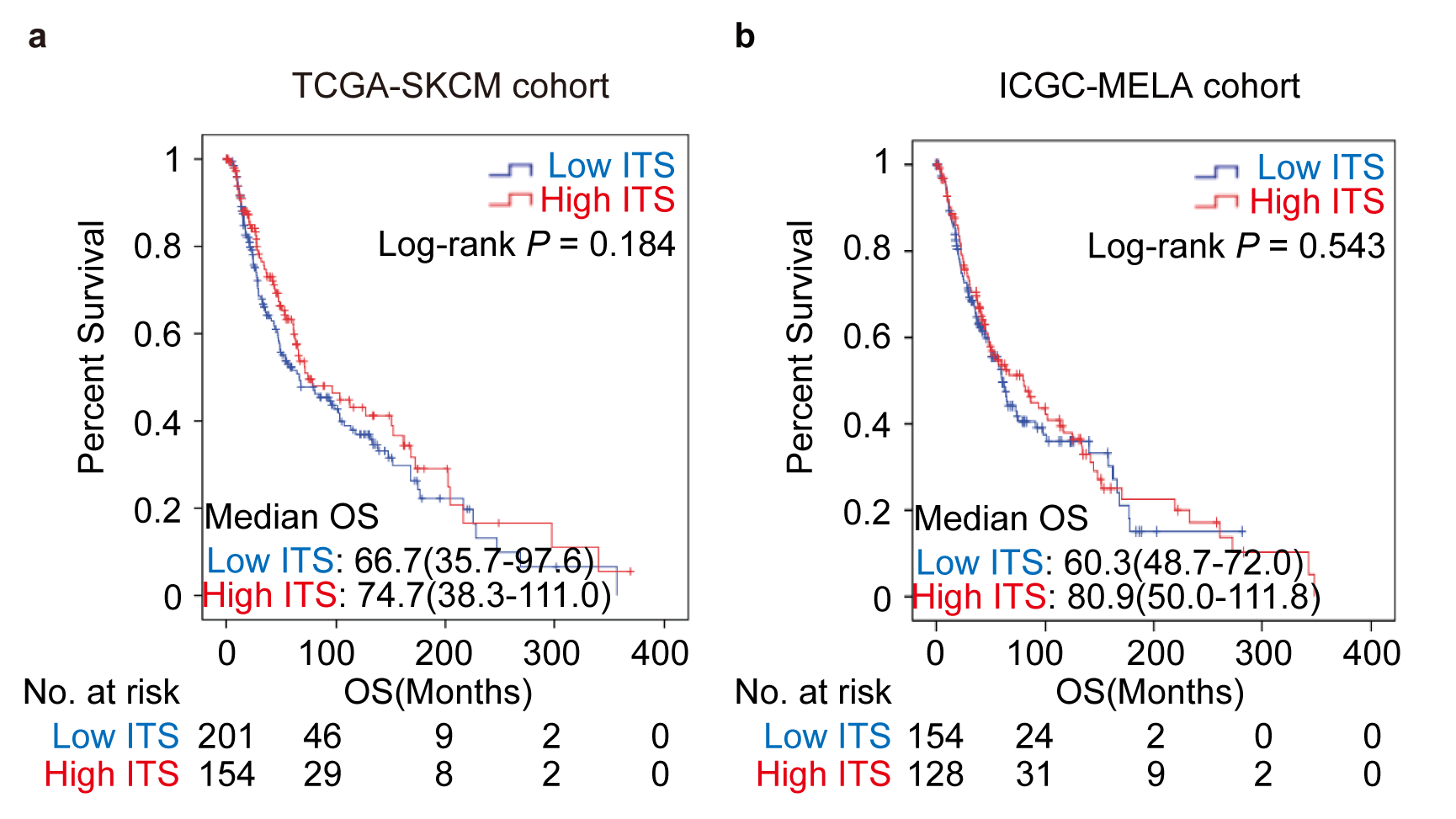

Supplement: Supplementary file 5 — Fig S5 [file CAM4-9-8498-s005.tif]

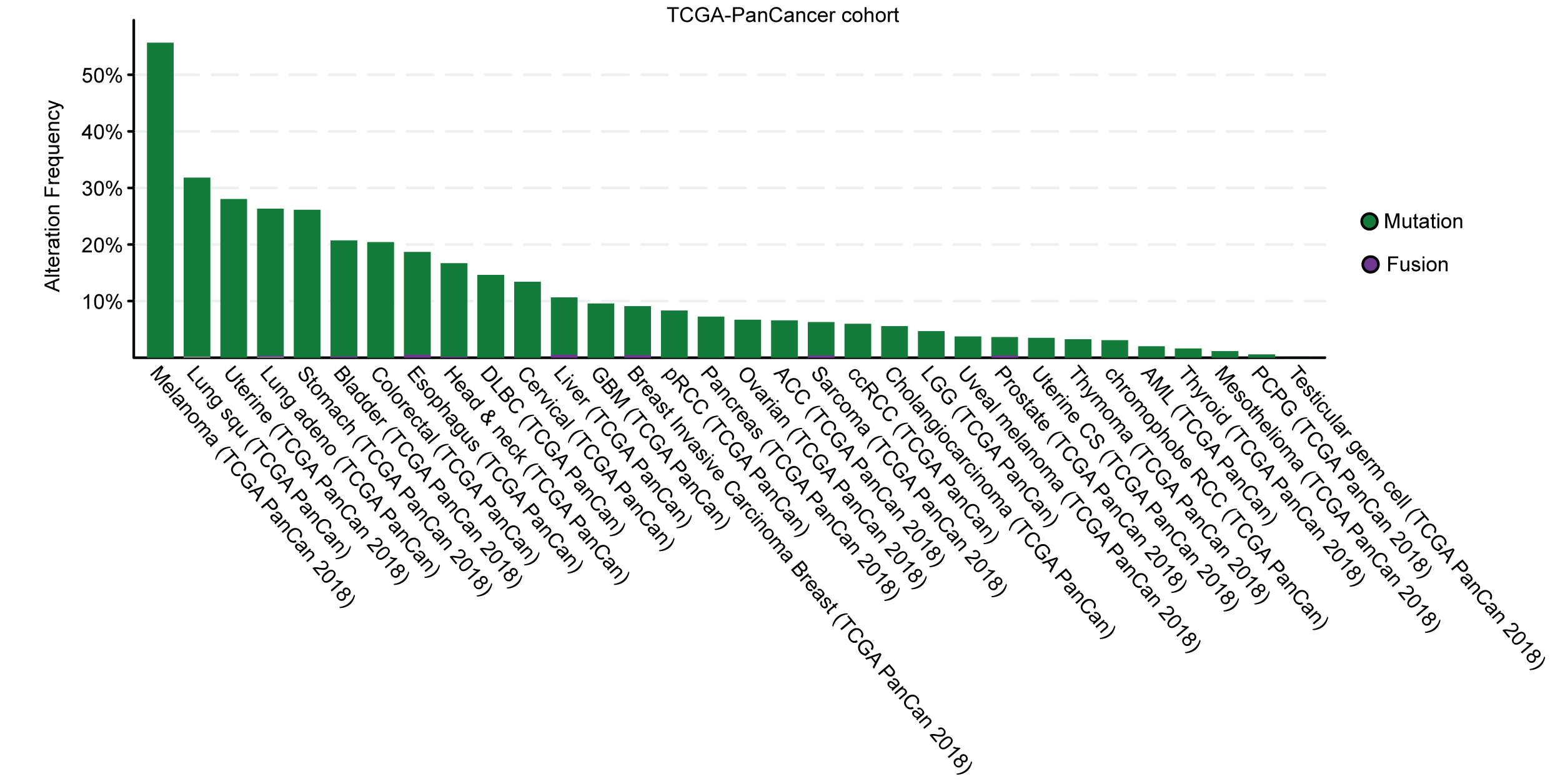

Supplement: Supplementary file 6 — Fig S6 [file CAM4-9-8498-s006.tif]

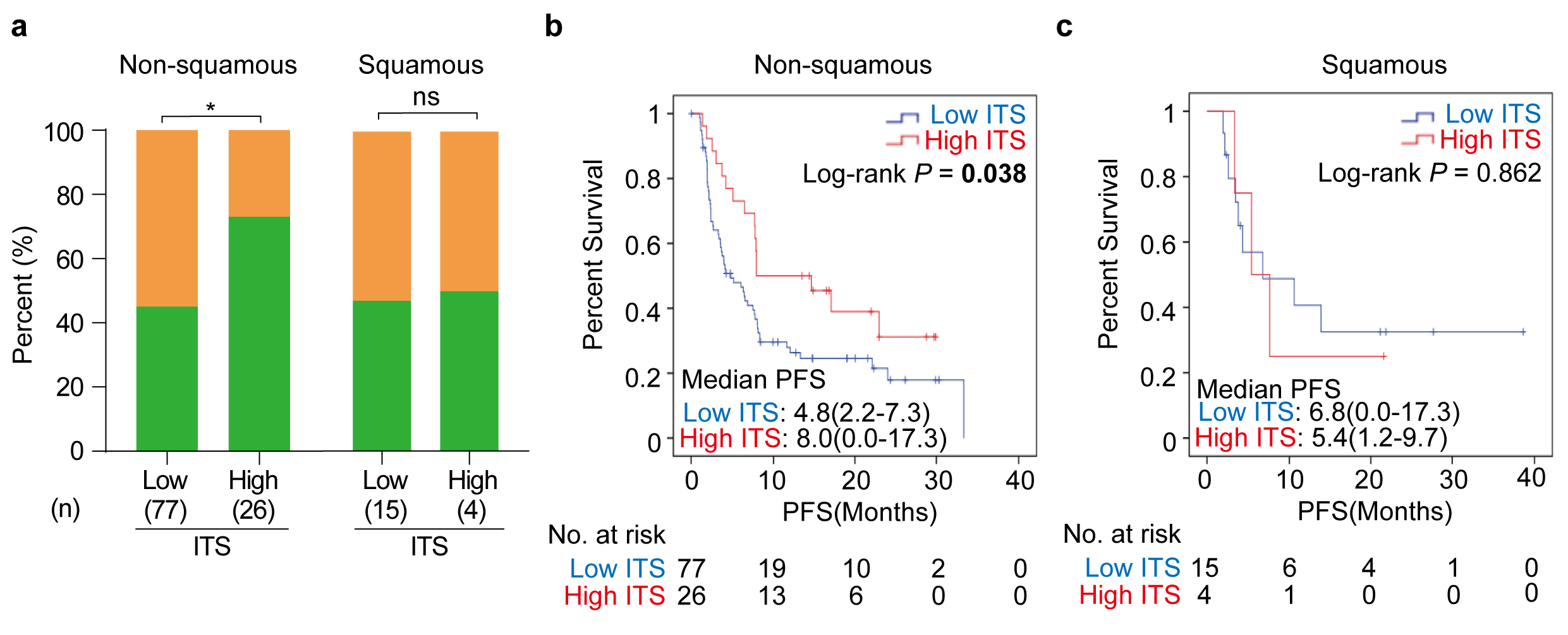

Supplement: Supplementary file 7 — Fig S7 [file CAM4-9-8498-s007.tif]

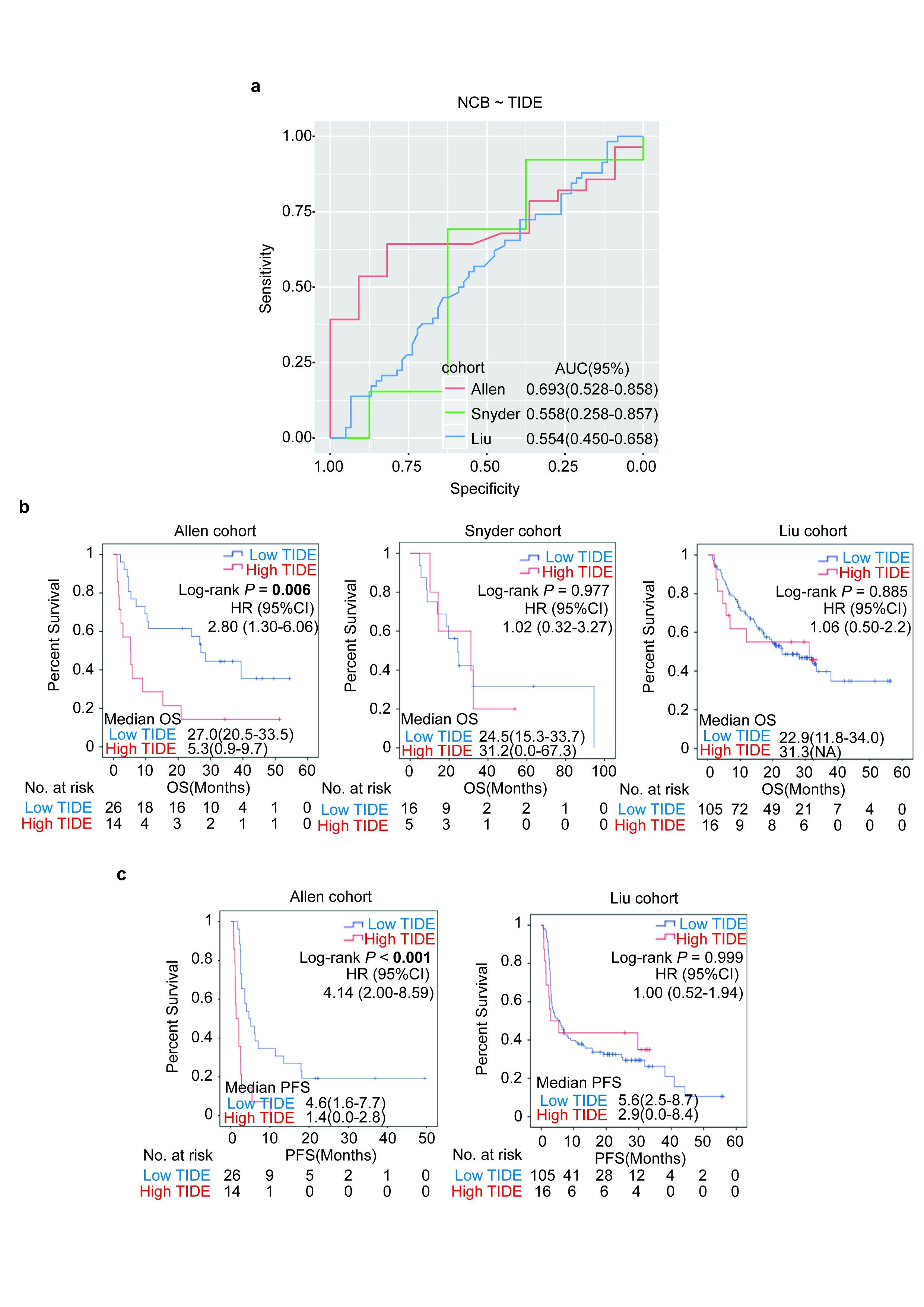

Supplement: Supplementary file 8 — Fig S8 [file CAM4-9-8498-s008.tif]
