# Supplementary material for: Integrated genomic analysis identifies a genetic mutation model predicting response to immune checkpoint inhibitors in melanoma
Source: Cancer Med. 2020 Sep 24;9(22):8498–518. doi: 10.1002/cam4.3481 (PMC7666739; doi:10.1002/cam4.3481)
Supplement: Supplementary file 9 — Table S1 [file CAM4-9-8498-s009.docx]

| **Table S1. The detailed clinical and genomic characteristics of melanoma patients with ICIs therapy in the Allen cohort, Snyder cohort and Liu cohort** | | | | | | | | | | | | | | | | | | | | | |
| --- | --- | --- | --- | --- | --- | --- | --- | --- | --- | --- | --- | --- | --- | --- | --- | --- | --- | --- | --- | --- | --- |
| ID | Cohort | Age | Gender | M stage | Primary site | LDH | Drug received | Nonsyn muts | TMB | NAL | Best objective response | OS time (months) | OS status | PFS time (months) | PFS status | Clinical benefit | ITS | *THSD7B* | *SYNE2* | *GRM3* | *FLNC* |
| Pat29 | Allen | 82 | Male | M1c | Cutaneous | Normal | Ipilimumab | 37 | 1.11 | 50 | NA | 44.2 | Living | 11.4 | Progressed | NA | 0 | 0 | 0 | 0 | 0 |
| Pat157 | Allen | 69 | Female | M1c | Cutaneous | Normal | Ipilimumab | 132 | 3.95 | 77 | NA | 2.9 | Deceased | 1.8 | Progressed | NA | 0 | 0 | 0 | 0 | 0 |
| Pat166 | Allen | 31 | Male | M1c | Occult | Elevated | Ipilimumab | 57 | 1.71 | 44 | NA | 2.6 | Deceased | 2.5 | Progressed | NA | 0 | 0 | 0 | 0 | 0 |
| Pat168 | Allen | 68 | Male | M1c | Cutaneous | Elevated | Ipilimumab | 245 | 7.34 | 306 | NA | 2.2 | Deceased | 2.2 | Progressed | NA | 1.636 | 0 | 0 | 0 | 1 |
| Pat175 | Allen | 67 | Female | M1a | Cutaneous | Normal | Ipilimumab | 89 | 2.66 | 69 | NA | 3.0 | Deceased | 3.0 | Progressed | NA | 0 | 0 | 0 | 0 | 0 |
| Pat03 | Allen | 61 | Female | M1c | Cutaneous | Elevated | Ipilimumab | 372 | 11.14 | 131 | PD | 3.3 | Deceased | 2.5 | Progressed | NCB | 0 | 0 | 0 | 0 | 0 |
| Pat06 | Allen | 33 | Male | M1c | Cutaneous | Normal | Ipilimumab | 174 | 5.21 | 167 | PD | 5.4 | Deceased | 2.6 | Progressed | NCB | 0 | 0 | 0 | 0 | 0 |
| Pat08 | Allen | 73 | Male | M1c | Mucosal | Normal | Ipilimumab | 265 | 7.93 | 311 | PD | 4.7 | Deceased | 2.4 | Progressed | NCB | 1.64 | 0 | 1 | 0 | 0 |
| Pat100 | Allen | 70 | Male | M1c | Cutaneous | Normal | Ipilimumab | 519 | 15.54 | 280 | PD | 12.0 | Deceased | 3.7 | Progressed | NCB | 1.636 | 0 | 0 | 0 | 1 |
| Pat101 | Allen | 75 | Male | M1c | Cutaneous | Elevated | Ipilimumab | 50 | 1.50 | 26 | PD | 9.6 | Deceased | 2.6 | Progressed | NCB | 0 | 0 | 0 | 0 | 0 |
| Pat106 | Allen | 43 | Female | M1b | Cutaneous | Normal | Ipilimumab | 30 | 0.90 | 28 | PD | 8.3 | Deceased | 4.6 | Progressed | NCB | 0.948 | 0 | 0 | 1 | 0 |
| Pat109 | Allen | 69 | Male | M1b | Cutaneous | Normal | Ipilimumab | 286 | 8.56 | 227 | PD | 2.8 | Deceased | 0.6 | Progressed | NCB | 0 | 0 | 0 | 0 | 0 |
| Pat11 | Allen | 67 | Male | M1c | Occult | Normal | Ipilimumab | 1976 | 59.16 | 1188 | PD | 26.3 | Living | 4.2 | Progressed | NCB | 2.87 | 1 | 0 | 0 | 1 |
| Pat110 | Allen | 76 | Male | M1b | Cutaneous | Normal | Ipilimumab | 6301 | 188.65 | 3644 | PD | 10.7 | Deceased | 2.4 | Progressed | NCB | 3.818 | 1 | 0 | 1 | 1 |
| Pat115 | Allen | 45 | Male | M1c | Cutaneous | Elevated | Ipilimumab | 76 | 2.28 | 69 | PD | 4.8 | Deceased | 2.5 | Progressed | NCB | 0 | 0 | 0 | 0 | 0 |
| Pat118 | Allen | 43 | Female | M1c | Cutaneous | Elevated | Ipilimumab | 113 | 3.38 | 60 | PD | 10.4 | Deceased | 4.0 | Progressed | NCB | 0 | 0 | 0 | 0 | 0 |
| Pat119 | Allen | 61 | Male | M0 | Cutaneous | Elevated | Ipilimumab | 301 | 9.01 | 201 | PD | 26.9 | Living | 3.6 | Progressed | NCB | 0 | 0 | 0 | 0 | 0 |
| Pat121 | Allen | 46 | Male | M1c | Occult | Normal | Ipilimumab | 63 | 1.89 | 24 | PD | 4.1 | Deceased | 2.8 | Progressed | NCB | 0 | 0 | 0 | 0 | 0 |
| Pat124 | Allen | 78 | Male | M1b | Cutaneous | Normal | Ipilimumab | 441 | 13.20 | 311 | PD | 4.9 | Deceased | 2.2 | Progressed | NCB | 1.234 | 1 | 0 | 0 | 0 |
| Pat127 | Allen | 25 | Female | M1c | Cutaneous | Normal | Ipilimumab | 109 | 3.26 | 62 | PD | 11.1 | Deceased | 2.8 | Progressed | NCB | 0 | 0 | 0 | 0 | 0 |
| Pat128 | Allen | 29 | Male | M1c | Cutaneous | Normal | Ipilimumab | 76 | 2.28 | 25 | PD | 3.8 | Deceased | 2.7 | Progressed | NCB | 0 | 0 | 0 | 0 | 0 |
| Pat129 | Allen | 18 | Male | M1b | Cutaneous | Normal | Ipilimumab | 107 | 3.20 | 85 | PD | 18.0 | Living | 2.3 | Progressed | NCB | 0 | 0 | 0 | 0 | 0 |
| Pat13 | Allen | 78 | Male | M0 | Cutaneous | Normal | Ipilimumab | 110 | 3.29 | 60 | PD | 24.4 | Deceased | 3.5 | Progressed | NCB | 0 | 0 | 0 | 0 | 0 |
| Pat130 | Allen | 76 | Male | M1c | Cutaneous | Elevated | Ipilimumab | 197 | 5.90 | 140 | PD | 1.5 | Deceased | 0.6 | Progressed | NCB | 0 | 0 | 0 | 0 | 0 |
| Pat133 | Allen | 76 | Male | M1b | Mucosal | Normal | Ipilimumab | 228 | 6.83 | 114 | PD | 18.1 | Deceased | 4.5 | Progressed | NCB | 0.948 | 0 | 0 | 1 | 0 |
| Pat135 | Allen | 29 | Female | M1c | Cutaneous | Normal | Ipilimumab | 238 | 7.13 | 227 | PD | 2.7 | Deceased | 2.4 | Progressed | NCB | 0 | 0 | 0 | 0 | 0 |
| Pat139 | Allen | 58 | Male | M1c | Cutaneous | Elevated | Ipilimumab | 1429 | 42.78 | 1918 | PD | 3.3 | Deceased | 1.5 | Progressed | NCB | 0.948 | 0 | 0 | 1 | 0 |
| Pat14 | Allen | 48 | Female | M1c | Cutaneous | Normal | Ipilimumab | 46 | 1.38 | 23 | PD | 5.4 | Deceased | 2.8 | Progressed | NCB | 0 | 0 | 0 | 0 | 0 |
| Pat140 | Allen | 62 | Male | M1c | Cutaneous | Normal | Ipilimumab | 326 | 9.76 | 257 | PD | 16.4 | Deceased | 3.8 | Progressed | NCB | 0 | 0 | 0 | 0 | 0 |
| Pat143 | Allen | 71 | Male | M1c | Cutaneous | Elevated | Ipilimumab | 878 | 26.29 | 1063 | PD | 5.1 | Deceased | 3.0 | Progressed | NCB | 1.234 | 1 | 0 | 0 | 0 |
| Pat147 | Allen | 63 | Male | M1a | Occult | Normal | Ipilimumab | 542 | 16.23 | 236 | PD | 7.5 | Deceased | 4.0 | Progressed | NCB | 0 | 0 | 0 | 0 | 0 |
| Pat148 | Allen | 35 | Female | M1c | Cutaneous | Normal | Ipilimumab | 227 | 6.80 | 131 | PD | 2.8 | Deceased | 1.6 | Progressed | NCB | 0 | 0 | 0 | 0 | 0 |
| Pat15 | Allen | 32 | Male | M1c | Cutaneous | Elevated | Ipilimumab | 242 | 7.25 | 271 | PD | 1.7 | Deceased | 1.0 | Progressed | NCB | 0.948 | 0 | 0 | 1 | 0 |
| Pat151 | Allen | 68 | Male | M1c | Cutaneous | Normal | Ipilimumab | 1754 | 52.51 | 1633 | PD | 6.8 | Deceased | 3.5 | Progressed | NCB | 2.588 | 0 | 1 | 1 | 0 |
| Pat159 | Allen | 51 | Male | M1b | Cutaneous | Normal | Ipilimumab | 471 | 14.10 | 139 | PD | 28.1 | Living | 3.3 | Progressed | NCB | 0.948 | 0 | 0 | 1 | 0 |
| Pat16 | Allen | 68 | Female | M1b | Cutaneous | Normal | Ipilimumab | 1911 | 57.22 | 1606 | PD | 27.0 | Deceased | 2.8 | Progressed | NCB | 1.234 | 1 | 0 | 0 | 0 |
| Pat160 | Allen | 79 | Male | M1c | Cutaneous | Elevated | Ipilimumab | 54 | 1.62 | 43 | PD | 5.1 | Deceased | 2.7 | Progressed | NCB | 0 | 0 | 0 | 0 | 0 |
| Pat162 | Allen | 55 | Female | M1c | Mucosal | Normal | Ipilimumab | 54 | 1.62 | 29 | PD | 7.0 | Deceased | 2.6 | Progressed | NCB | 0 | 0 | 0 | 0 | 0 |
| Pat163 | Allen | 54 | Male | M1c | Occult | Normal | Ipilimumab | 94 | 2.81 | 93 | PD | 25.0 | Living | 2.5 | Progressed | NCB | 0 | 0 | 0 | 0 | 0 |
| Pat165 | Allen | 50 | Female | M1c | Cutaneous | Normal | Ipilimumab | 69 | 2.07 | 76 | PD | 3.8 | Deceased | 3.2 | Progressed | NCB | 0 | 0 | 0 | 0 | 0 |
| Pat167 | Allen | 50 | Male | M1c | Cutaneous | Elevated | Ipilimumab | 33 | 0.99 | 27 | PD | 13.6 | Deceased | 2.8 | Progressed | NCB | 0 | 0 | 0 | 0 | 0 |
| Pat17 | Allen | 44 | Male | M1c | Cutaneous | Elevated | Ipilimumab | 219 | 6.56 | 59 | PD | 6.8 | Deceased | 2.8 | Progressed | NCB | 0 | 0 | 0 | 0 | 0 |
| Pat170 | Allen | 48 | Male | M1c | Cutaneous | Normal | Ipilimumab | 234 | 7.01 | 181 | PD | 3.6 | Deceased | 3.2 | Progressed | NCB | 0 | 0 | 0 | 0 | 0 |
| Pat171 | Allen | 66 | Male | M1a | Cutaneous | NA | Ipilimumab | 99 | 2.96 | 40 | PD | 15.5 | Deceased | 2.8 | Progressed | NCB | 1.234 | 1 | 0 | 0 | 0 |
| Pat18 | Allen | 55 | Female | M0 | Cutaneous | Normal | Ipilimumab | 32 | 0.96 | 5 | PD | 26.6 | Living | 3.7 | Progressed | NCB | 0 | 0 | 0 | 0 | 0 |
| Pat19 | Allen | 59 | Male | M1c | Cutaneous | Normal | Ipilimumab | 470 | 14.07 | 416 | PD | 5.8 | Deceased | 0.7 | Progressed | NCB | 1.64 | 0 | 1 | 0 | 0 |
| Pat25 | Allen | 69 | Male | M1c | Cutaneous | Elevated | Ipilimumab | 51 | 1.53 | 13 | PD | 10.8 | Deceased | 2.2 | Progressed | NCB | 0 | 0 | 0 | 0 | 0 |
| Pat27 | Allen | 61 | Male | M1c | Cutaneous | Normal | Ipilimumab | 33 | 0.99 | 15 | PD | 46.0 | Living | 4.6 | Progressed | NCB | 0 | 0 | 0 | 0 | 0 |
| Pat28 | Allen | 77 | Male | M1c | Cutaneous | Elevated | Ipilimumab | 750 | 22.46 | 508 | PD | 39.5 | Deceased | 2.6 | Progressed | NCB | 0 | 0 | 0 | 0 | 0 |
| Pat32 | Allen | 72 | Male | M1c | Occult | Elevated | Ipilimumab | 714 | 21.38 | 591 | PD | 4.9 | Deceased | 2.3 | Progressed | NCB | 0 | 0 | 0 | 0 | 0 |
| Pat33 | Allen | 65 | Male | M1c | Cutaneous | Elevated | Ipilimumab | 15 | 0.45 | 8 | PD | 7.0 | Deceased | 7.0 | Progressed | NCB | 0 | 0 | 0 | 0 | 0 |
| Pat36 | Allen | 52 | Female | M1c | Cutaneous | Elevated | Ipilimumab | 12 | 0.36 | 6 | PD | 1.8 | Deceased | 1.4 | Progressed | NCB | 0 | 0 | 0 | 0 | 0 |
| Pat37 | Allen | 47 | Female | M1c | Cutaneous | Elevated | Ipilimumab | 108 | 3.23 | 30 | PD | 2.3 | Deceased | 0.8 | Progressed | NCB | 0 | 0 | 0 | 0 | 0 |
| Pat40 | Allen | 74 | Male | M1c | Cutaneous | Elevated | Ipilimumab | 108 | 3.23 | 89 | PD | 1.1 | Deceased | 1.1 | Progressed | NCB | 0 | 0 | 0 | 0 | 0 |
| Pat41 | Allen | 64 | Male | M1c | Cutaneous | Elevated | Ipilimumab | 345 | 10.33 | 261 | PD | 4.3 | Deceased | 1.8 | Progressed | NCB | 0 | 0 | 0 | 0 | 0 |
| Pat43 | Allen | 75 | Female | M1b | Cutaneous | Elevated | Ipilimumab | 168 | 5.03 | 60 | PD | 1.2 | Deceased | 1.2 | Progressed | NCB | 0.948 | 0 | 0 | 1 | 0 |
| Pat44 | Allen | 57 | Female | M1c | Cutaneous | Elevated | Ipilimumab | 32 | 0.96 | 15 | PD | 9.0 | Deceased | 2.1 | Progressed | NCB | 0 | 0 | 0 | 0 | 0 |
| Pat45 | Allen | 68 | Male | M1c | Cutaneous | Elevated | Ipilimumab | 937 | 28.05 | 713 | PD | 3.0 | Deceased | 2.0 | Progressed | NCB | 1.234 | 1 | 0 | 0 | 0 |
| Pat46 | Allen | 36 | Female | M1b | Occult | Normal | Ipilimumab | 244 | 7.31 | 80 | PD | 5.3 | Deceased | 1.2 | Progressed | NCB | 1.234 | 1 | 0 | 0 | 0 |
| Pat50 | Allen | 77 | Male | M1c | Cutaneous | Normal | Ipilimumab | 444 | 13.29 | 523 | PD | 2.2 | Deceased | 2.2 | Progressed | NCB | 0 | 0 | 0 | 0 | 0 |
| Pat54 | Allen | 73 | Male | M1c | Cutaneous | Elevated | Ipilimumab | 866 | 25.93 | 679 | PD | 6.9 | Deceased | 2.7 | Progressed | NCB | 0.948 | 0 | 0 | 1 | 0 |
| Pat55 | Allen | 71 | Female | M1c | Cutaneous | Elevated | Ipilimumab | 484 | 14.49 | 483 | PD | 6.4 | Deceased | 2.6 | Progressed | NCB | 0 | 0 | 0 | 0 | 0 |
| Pat56 | Allen | 68 | Male | M1c | Cutaneous | Elevated | Ipilimumab | 44 | 1.32 | 38 | PD | 7.8 | Deceased | 3.2 | Progressed | NCB | 0 | 0 | 0 | 0 | 0 |
| Pat57 | Allen | 69 | Male | M0 | Cutaneous | Elevated | Ipilimumab | 56 | 1.68 | 32 | PD | 8.3 | Deceased | 2.3 | Progressed | NCB | 0 | 0 | 0 | 0 | 0 |
| Pat58 | Allen | 59 | Female | M0 | Cutaneous | Elevated | Ipilimumab | 1921 | 57.51 | 1971 | PD | 21.7 | Deceased | 3.3 | Progressed | NCB | 1.64 | 0 | 1 | 0 | 0 |
| Pat59 | Allen | 36 | Male | M1b | Cutaneous | Elevated | Ipilimumab | 273 | 8.17 | 309 | PD | 7.5 | Deceased | 2.5 | Progressed | NCB | 1.636 | 0 | 0 | 0 | 1 |
| Pat60 | Allen | 86 | Male | M1c | Cutaneous | Elevated | Ipilimumab | 738 | 22.10 | 472 | PD | 9.1 | Deceased | 3.0 | Progressed | NCB | 1.234 | 1 | 0 | 0 | 0 |
| Pat62 | Allen | 76 | Male | M1c | Cutaneous | Elevated | Ipilimumab | 1379 | 41.29 | 1340 | PD | 20.1 | Deceased | 2.6 | Progressed | NCB | 1.234 | 1 | 0 | 0 | 0 |
| Pat64 | Allen | 83 | Male | M1c | Cutaneous | Elevated | Ipilimumab | 659 | 19.73 | 559 | PD | 3.5 | Deceased | 2.3 | Progressed | NCB | 1.234 | 1 | 0 | 0 | 0 |
| Pat67 | Allen | 39 | Male | M1c | Cutaneous | Elevated | Ipilimumab | 27 | 0.81 | 11 | PD | 2.6 | Deceased | 2.6 | Progressed | NCB | 1.234 | 1 | 0 | 0 | 0 |
| Pat70 | Allen | 38 | Male | M1c | Cutaneous | Elevated | Ipilimumab | 77 | 2.31 | 38 | PD | 17.7 | Deceased | 0.5 | Progressed | NCB | 0 | 0 | 0 | 0 | 0 |
| Pat71 | Allen | 63 | Male | M1c | Occult | Normal | Ipilimumab | 722 | 21.62 | 726 | PD | 4.6 | Deceased | 3.3 | Progressed | NCB | 0 | 0 | 0 | 0 | 0 |
| Pat74 | Allen | 73 | Male | M1c | Cutaneous | Normal | Ipilimumab | 509 | 15.24 | 611 | PD | 6.4 | Deceased | 2.7 | Progressed | NCB | 0.948 | 0 | 0 | 1 | 0 |
| Pat76 | Allen | 74 | Male | M1c | Occult | Elevated | Ipilimumab | 198 | 5.93 | 82 | PD | 4.6 | Deceased | 2.3 | Progressed | NCB | 0.948 | 0 | 0 | 1 | 0 |
| Pat78 | Allen | 71 | Female | M1c | Cutaneous | Elevated | Ipilimumab | 154 | 4.61 | 76 | PD | 1.3 | Deceased | 1.3 | Progressed | NCB | 0 | 0 | 0 | 0 | 0 |
| Pat81 | Allen | 54 | Female | M1a | Mucosal | Normal | Ipilimumab | 111 | 3.32 | 96 | PD | 20.9 | Deceased | 2.5 | Progressed | NCB | 0 | 0 | 0 | 0 | 0 |
| Pat82 | Allen | 54 | Female | M1c | Cutaneous | NA | Ipilimumab | 408 | 12.22 | 179 | PD | 3.5 | Deceased | 2.5 | Progressed | NCB | 0 | 0 | 0 | 0 | 0 |
| Pat83 | Allen | 22 | Male | M1c | Cutaneous | Normal | Ipilimumab | 37 | 1.11 | 15 | PD | 34.2 | Living | 3.6 | Progressed | NCB | 0 | 0 | 0 | 0 | 0 |
| Pat85 | Allen | 83 | Male | M1c | Cutaneous | Normal | Ipilimumab | 408 | 12.22 | 239 | PD | 15.3 | Deceased | 2.8 | Progressed | NCB | 3.822 | 1 | 1 | 1 | 0 |
| Pat92 | Allen | 49 | Male | M1c | Cutaneous | Elevated | Ipilimumab | 39 | 1.17 | 13 | PD | 4.1 | Deceased | 2.8 | Progressed | NCB | 0 | 0 | 0 | 0 | 0 |
| Pat98 | Allen | 57 | Female | M1c | Cutaneous | Normal | Ipilimumab | 33 | 0.99 | 16 | PD | 4.7 | Deceased | 2.6 | Progressed | NCB | 0 | 0 | 0 | 0 | 0 |
| Pat49 | Allen | 36 | Male | M1c | Cutaneous | Normal | Ipilimumab | 669 | 20.03 | 323 | SD | 34.5 | Living | 5.4 | Progressed | NCB | 1.234 | 1 | 0 | 0 | 0 |
| Pat86 | Allen | 55 | Male | M1a | Cutaneous | Normal | Ipilimumab | 62 | 1.86 | 38 | SD | 9.8 | Deceased | 5.1 | Progressed | NCB | 0 | 0 | 0 | 0 | 0 |
| Pat39 | Allen | 67 | Male | M1b | Cutaneous | Normal | Ipilimumab | 75 | 2.25 | 62 | CR | 49.6 | Living | 49.6 | Living | DCB | 0 | 0 | 0 | 0 | 0 |
| Pat47 | Allen | 78 | Male | M1c | Cutaneous | Normal | Ipilimumab | 137 | 4.10 | 112 | CR | 36.9 | Living | 36.9 | Living | DCB | 1.234 | 1 | 0 | 0 | 0 |
| Pat63 | Allen | 65 | Male | M0 | Cutaneous | NA | Ipilimumab | 209 | 6.26 | 48 | CR | 34.6 | Living | 23.9 | Living | DCB | 2.182 | 1 | 0 | 1 | 0 |
| Pat04 | Allen | 71 | Male | M1b | Cutaneous | Normal | Ipilimumab | 336 | 10.06 | 271 | PR | 32.9 | Living | 21.5 | Living | DCB | 0.948 | 0 | 0 | 1 | 0 |
| Pat103 | Allen | 70 | Male | M1c | Occult | Normal | Ipilimumab | 630 | 18.86 | 348 | PR | 34.9 | Deceased | 32.3 | Living | DCB | 1.64 | 0 | 1 | 0 | 0 |
| Pat105 | Allen | 41 | Male | M0 | Cutaneous | Normal | Ipilimumab | 125 | 3.74 | 66 | PR | 34.9 | Living | 23.0 | Living | DCB | 1.64 | 0 | 1 | 0 | 0 |
| Pat113 | Allen | 68 | Male | M1c | Occult | Elevated | Ipilimumab | 317 | 9.49 | 139 | PR | 10.0 | Deceased | 1.7 | Progressed | DCB | 2.584 | 0 | 0 | 1 | 1 |
| Pat117 | Allen | 73 | Male | M1c | Cutaneous | Elevated | Ipilimumab | 689 | 20.63 | 549 | PR | 30.5 | Living | 6.1 | Progressed | DCB | 2.182 | 1 | 0 | 1 | 0 |
| Pat126 | Allen | 77 | Male | M1b | Cutaneous | Elevated | Ipilimumab | 397 | 11.89 | 238 | PR | 21.4 | Living | 6.2 | Progressed | DCB | 1.636 | 0 | 0 | 0 | 1 |
| Pat132 | Allen | 81 | Male | M1c | Cutaneous | Normal | Ipilimumab | 1862 | 55.75 | 910 | PR | 22.5 | Living | 6.2 | Progressed | DCB | 1.64 | 0 | 1 | 0 | 0 |
| Pat174 | Allen | 57 | Female | M1a | Cutaneous | Normal | Ipilimumab | 668 | 20.00 | 355 | PR | 22.9 | Living | 4.5 | Progressed | DCB | 2.182 | 1 | 0 | 1 | 0 |
| Pat24 | Allen | 74 | Female | M1b | Cutaneous | Normal | Ipilimumab | 33 | 0.99 | 22 | PR | 32.4 | Living | 21.9 | Living | DCB | 0 | 0 | 0 | 0 | 0 |
| Pat38 | Allen | 45 | Male | M1c | Cutaneous | Normal | Ipilimumab | 2398 | 71.80 | 2503 | PR | 51.3 | Living | 10.1 | Progressed | DCB | 2.584 | 0 | 0 | 1 | 1 |
| Pat73 | Allen | 71 | Male | M1c | Cutaneous | Elevated | Ipilimumab | 243 | 7.28 | 281 | PR | 14.7 | Deceased | 13.6 | Progressed | DCB | 0 | 0 | 0 | 0 | 0 |
| Pat77 | Allen | 21 | Male | M0 | Occult | Normal | Ipilimumab | 375 | 11.23 | 251 | PR | 34.3 | Living | 8.0 | Living | DCB | 0 | 0 | 0 | 0 | 0 |
| Pat79 | Allen | 69 | Male | M1b | Cutaneous | Normal | Ipilimumab | 321 | 9.61 | 143 | PR | 26.7 | Deceased | 13.5 | Progressed | DCB | 1.234 | 1 | 0 | 0 | 0 |
| Pat90 | Allen | 59 | Male | M1c | Cutaneous | Normal | Ipilimumab | 207 | 6.20 | 115 | PR | 33.5 | Living | 22.3 | Living | DCB | 0.948 | 0 | 0 | 1 | 0 |
| Pat02 | Allen | 42 | Female | M1c | Occult | Elevated | Ipilimumab | 233 | 6.98 | 113 | SD | 54.4 | Living | 17.9 | Progressed | DCB | 1.636 | 0 | 0 | 0 | 1 |
| Pat07 | Allen | 36 | Male | M0 | Cutaneous | Normal | Ipilimumab | 148 | 4.43 | 99 | SD | 35.0 | Living | 24.0 | Progressed | DCB | 3.276 | 0 | 1 | 0 | 1 |
| Pat123 | Allen | 50 | Female | M1c | Occult | Elevated | Ipilimumab | 482 | 14.43 | 255 | SD | 28.4 | Deceased | 18.1 | Progressed | DCB | 1.234 | 1 | 0 | 0 | 0 |
| Pat138 | Allen | 39 | Female | M1c | Cutaneous | Normal | Ipilimumab | 5120 | 153.29 | 3166 | SD | 49.3 | Deceased | 19.4 | Progressed | DCB | 5.458 | 1 | 1 | 1 | 1 |
| Pat21 | Allen | 81 | Male | M1c | Cutaneous | Normal | Ipilimumab | 1375 | 41.17 | 679 | SD | 22.5 | Deceased | 18.6 | Progressed | DCB | 2.874 | 1 | 1 | 0 | 0 |
| Pat66 | Allen | 44 | Female | M0 | Cutaneous | Elevated | Ipilimumab | 327 | 9.79 | 242 | SD | 21.8 | Living | 19.9 | Progressed | DCB | 2.182 | 1 | 0 | 1 | 0 |
| Pat80 | Allen | 48 | Male | M1c | Cutaneous | Elevated | Ipilimumab | 193 | 5.78 | 154 | SD | 24.1 | Deceased | 6.1 | Progressed | DCB | 0 | 0 | 0 | 0 | 0 |
| Pat88 | Allen | 60 | Female | M1c | Cutaneous | NA | Ipilimumab | 1597 | 47.81 | 716 | SD | 33.0 | Living | 22.2 | Living | DCB | 3.822 | 1 | 1 | 1 | 0 |
| Pat104 | Allen | 45 | Female | M1c | Cutaneous | Normal | Ipilimumab | 35 | 1.05 | 32 | SD | 7.9 | Deceased | 7.9 | Living | DCB | 0 | 0 | 0 | 0 | 0 |
| Pat131 | Allen | 32 | Male | M1c | Cutaneous | Elevated | Ipilimumab | 102 | 3.05 | 74 | SD | 8.6 | Deceased | 6.5 | Progressed | DCB | 0 | 0 | 0 | 0 | 0 |
| CR0095 | Snyder | 74 | Male | M1b | Cutaneous | Normal | Ipilimumab | 282 | 8.44 | 186 | NA | 67.9 | Living | NA | NA | DCB | 1.636 | 0 | 0 | 0 | 1 |
| CR04885 | Snyder | 49 | Female | M0 | Cutaneous | NA | Tremelimumab | 2102 | 62.93 | 1993 | NA | 25.6 | Living | NA | NA | DCB | 5.458 | 1 | 1 | 1 | 1 |
| CR06670 | Snyder | 79 | Female | M0 | Cutaneous | NA | Tremelimumab | 590 | 17.66 | 358 | NA | 44.4 | Deceased | NA | NA | DCB | 2.87 | 1 | 0 | 0 | 1 |
| CR1509 | Snyder | 54 | Female | M1c | Cutaneous | Normal | Ipilimumab | 539 | 16.14 | 245 | NA | 53.9 | Living | NA | NA | DCB | 0 | 0 | 0 | 0 | 0 |
| CR22640 | Snyder | 71 | Male | M1c | Cutaneous | NA | Ipilimumab | 334 | 10.00 | 217 | NA | 51.2 | Living | NA | NA | DCB | 1.234 | 1 | 0 | 0 | 0 |
| CR3665 | Snyder | 70 | Male | M1b | Cutaneous | Normal | Ipilimumab | 141 | 4.22 | 45 | NA | 31.9 | Living | NA | NA | DCB | 0 | 0 | 0 | 0 | 0 |
| CR4880 | Snyder | 63 | Male | M1b | Occult | Normal | Ipilimumab | 2570 | 76.95 | 2026 | NA | 64.3 | Living | NA | NA | DCB | 1.234 | 1 | 0 | 0 | 0 |
| CR6126 | Snyder | 66 | Female | M1b | Cutaneous | Normal | Ipilimumab | 562 | 16.83 | 450 | NA | 22.8 | Living | NA | NA | DCB | 2.182 | 1 | 0 | 1 | 0 |
| CR6161 | Snyder | 81 | Male | M1b | Cutaneous | NA | Ipilimumab | 930 | 27.84 | 959 | NA | 31.3 | Deceased | NA | NA | DCB | 2.87 | 1 | 0 | 0 | 1 |
| CR7623 | Snyder | 65 | Male | M1c | Cutaneous | Elevated | Ipilimumab | 503 | 15.06 | 425 | NA | 63.5 | Living | NA | NA | NCB | 1.636 | 0 | 0 | 0 | 1 |
| CR9306 | Snyder | 70 | Male | M1c | Cutaneous | Normal | Ipilimumab | 1389 | 41.59 | 214 | NA | 52.8 | Living | NA | NA | DCB | 0 | 0 | 0 | 0 | 0 |
| CR9699 | Snyder | 33 | Male | M1c | Cutaneous | Normal | Ipilimumab | 1228 | 36.77 | 413 | NA | 32.4 | Living | NA | NA | DCB | 3.276 | 0 | 1 | 0 | 1 |
| CRNR0244 | Snyder | 36 | Female | M1c | Occult | Normal | Ipilimumab | 543 | 16.26 | 333 | NA | 40.1 | Living | NA | NA | NCB | 0 | 0 | 0 | 0 | 0 |
| CRNR2472 | Snyder | 74 | Male | M1c | Cutaneous | Normal | Ipilimumab | 627 | 18.77 | 666 | NA | 34.8 | Living | NA | NA | NCB | 1.636 | 0 | 0 | 0 | 1 |
| CRNR4941 | Snyder | 62 | Male | M1c | Occult | NA | Ipilimumab | 117 | 3.50 | 125 | NA | 87.6 | Living | NA | NA | NCB | 0 | 0 | 0 | 0 | 0 |
| LSD0167 | Snyder | 52 | Male | M1c | Cutaneous | Normal | Ipilimumab | 399 | 11.95 | 344 | NA | 25.2 | Living | NA | NA | DCB | 1.636 | 0 | 0 | 0 | 1 |
| LSD2057 | Snyder | 55 | Male | M1c | Cutaneous | Elevated | Ipilimumab | 171 | 5.12 | 93 | NA | 40.8 | Living | NA | NA | DCB | 1.234 | 1 | 0 | 0 | 0 |
| LSD3484 | Snyder | 66 | Male | M1c | Cutaneous | Elevated | Tremelimumab | 531 | 15.90 | 529 | NA | 18.7 | Deceased | NA | NA | DCB | 2.87 | 1 | 0 | 0 | 1 |
| LSD4691 | Snyder | 78 | Male | M1c | Cutaneous | NA | Ipilimumab | 393 | 11.77 | 104 | NA | 50.2 | Living | NA | NA | DCB | 1.64 | 0 | 1 | 0 | 0 |
| LSD4744 | Snyder | 44 | Male | M1c | Cutaneous | Normal | Ipilimumab | 1124 | 33.65 | 1469 | NA | 25.3 | Living | NA | NA | DCB | 1.234 | 1 | 0 | 0 | 0 |
| LSD6819 | Snyder | 75 | Male | M1c | Acral | Normal | Ipilimumab | 216 | 6.47 | 123 | NA | 72.7 | Living | NA | NA | DCB | 0 | 0 | 0 | 0 | 0 |
| LSDNR1120 | Snyder | 65 | Female | M1a | Occult | Normal | Ipilimumab | 537 | 16.08 | 384 | NA | 72.4 | Living | NA | NA | NCB | 1.234 | 1 | 0 | 0 | 0 |
| LSDNR1650 | Snyder | 57 | Female | M1c | NA | Elevated | Ipilimumab | 25 | 0.75 | 10 | NA | 72.8 | Living | NA | NA | NCB | 0 | 0 | 0 | 0 | 0 |
| LSDNR3086 | Snyder | 43 | Female | M1c | Cutaneous | NA | Ipilimumab+dacarbazine | 54 | 1.62 | 43 | NA | 34.8 | Deceased | NA | NA | NCB | 1.636 | 0 | 0 | 0 | 1 |
| LSDNR9298 | Snyder | 70 | Male | M1c | Cutaneous | Normal | Ipilimumab | 1277 | 38.23 | 960 | NA | 23.7 | Living | NA | NA | NCB | 2.87 | 1 | 0 | 0 | 1 |
| NR1867 | Snyder | 39 | Female | M1c | Acral | Normal | Ipilimumab | 80 | 2.40 | 75 | NA | 47.2 | Living | NA | NA | NCB | 0 | 0 | 0 | 0 | 0 |
| NR2137 | Snyder | 53 | Male | M1c | Cutaneous | Elevated | Ipilimumab | 635 | 19.01 | 764 | NA | 83.4 | Living | NA | NA | NCB | 1.636 | 0 | 0 | 0 | 1 |
| NR3156 | Snyder | 62 | Male | M1b | Cutaneous | Normal | Ipilimumab | 6 | 0.18 | 5 | NA | 64.8 | Living | NA | NA | NCB | 0 | 0 | 0 | 0 | 0 |
| NR3549 | Snyder | 49 | Male | M1c | Cutaneous | Elevated | Ipilimumab | 365 | 10.93 | 268 | NA | 25.2 | Living | NA | NA | NCB | 0 | 0 | 0 | 0 | 0 |
| NR4018 | Snyder | 71 | Male | M1c | NA | NA | Ipilimumab | 146 | 4.37 | 142 | NA | 20.4 | Living | NA | NA | NCB | 0 | 0 | 0 | 0 | 0 |
| NR4045 | Snyder | 40 | Female | M1c | Cutaneous | Normal | Ipilimumab | 451 | 13.50 | 561 | NA | 86.4 | Living | NA | NA | NCB | 0.948 | 0 | 0 | 1 | 0 |
| NR4083 | Snyder | 68 | Male | M1c | NA | NA | Ipilimumab | 444 | 13.29 | 246 | NA | 55.2 | Living | NA | NA | NCB | 0 | 0 | 0 | 0 | 0 |
| NR4631 | Snyder | 71 | Female | M1c | Cutaneous | Elevated | Ipilimumab | 1165 | 34.88 | 864 | NA | 32.4 | Deceased | NA | NA | NCB | 1.234 | 1 | 0 | 0 | 0 |
| NR4810 | Snyder | 43 | Male | M1c | Cutaneous | Elevated | Ipilimumab | 358 | 10.72 | 237 | NA | 31.2 | Deceased | NA | NA | NCB | 4.51 | 1 | 1 | 0 | 1 |
| NR4949 | Snyder | 82 | Female | M1c | Occult | Normal | Ipilimumab | 513 | 15.36 | 294 | NA | 50.0 | Living | NA | NA | NCB | 0 | 0 | 0 | 0 | 0 |
| NR5784 | Snyder | 68 | Male | M1c | Cutaneous | Elevated | Ipilimumab | 39 | 1.17 | 32 | NA | 18.5 | Deceased | NA | NA | NCB | 0 | 0 | 0 | 0 | 0 |
| NR6689 | Snyder | 18 | Male | M1c | Cutaneous | Normal | Ipilimumab | 6 | 0.18 | 7 | NA | 9.8 | Living | NA | NA | NCB | 0 | 0 | 0 | 0 | 0 |
| NR6721 | Snyder | 68 | Female | M1a | Cutaneous | Normal | Ipilimumab | 128 | 3.83 | 22 | NA | 12.6 | Deceased | NA | NA | NCB | 1.234 | 1 | 0 | 0 | 0 |
| NR6842 | Snyder | 50 | Male | M1c | Cutaneous | Normal | Ipilimumab | 3 | 0.09 | 3 | NA | 6.2 | Deceased | NA | NA | NCB | 0 | 0 | 0 | 0 | 0 |
| NR8727 | Snyder | 55 | Male | M1c | Cutaneous | Normal | Ipilimumab | 2 | 0.06 | 0 | NA | 3.3 | Living | NA | NA | NCB | 0 | 0 | 0 | 0 | 0 |
| NR8815 | Snyder | 42 | Male | M1b | Cutaneous | Normal | Ipilimumab | 1699 | 50.87 | 801 | NA | 20.4 | Deceased | NA | NA | NCB | 0.948 | 0 | 0 | 1 | 0 |
| NR9341 | Snyder | 54 | Male | M1a | Cutaneous | NA | Ipilimumab | 323 | 9.67 | 243 | NA | 2.7 | Deceased | NA | NA | NCB | 0 | 0 | 0 | 0 | 0 |
| NR9445 | Snyder | 59 | Male | M1c | Cutaneous | Elevated | Ipilimumab | 267 | 7.99 | 187 | NA | 6.0 | Deceased | NA | NA | NCB | 1.234 | 1 | 0 | 0 | 0 |
| NR9449 | Snyder | 48 | Female | M1c | Cutaneous | Normal | Ipilimumab | 295 | 8.83 | 223 | NA | 4.8 | Deceased | NA | NA | NCB | 0 | 0 | 0 | 0 | 0 |
| NR9521 | Snyder | 79 | Female | M1b | Cutaneous | Normal | Ipilimumab | 1061 | 31.77 | 591 | NA | 8.4 | Deceased | NA | NA | NCB | 0 | 0 | 0 | 0 | 0 |
| NR9705 | Snyder | 58 | Female | M1c | Acral | Normal | Ipilimumab | 37 | 1.11 | 21 | NA | 5.5 | Deceased | NA | NA | NCB | 0 | 0 | 0 | 0 | 0 |
| NR9765 | Snyder | 46 | Female | M1b | Cutaneous | Normal | Ipilimumab | 1148 | 34.37 | 112 | NA | 15.0 | Deceased | NA | NA | NCB | 0 | 0 | 0 | 0 | 0 |
| PR03803 | Snyder | 64 | Male | M0 | Cutaneous | NA | Ipilimumab | 269 | 8.05 | 190 | NA | 9.6 | Deceased | NA | NA | NCB | 0.948 | 0 | 0 | 1 | 0 |
| PR12117 | Snyder | 64 | Male | M1c | Cutaneous | NA | Ipilimumab | 1081 | 32.37 | 909 | NA | 9.8 | Deceased | NA | NA | DCB | 4.224 | 0 | 1 | 1 | 1 |
| PR4035 | Snyder | 69 | Male | M1c | NA | NA | Ipilimumab | 525 | 15.72 | 494 | NA | 5.2 | Deceased | NA | NA | DCB | 0 | 0 | 0 | 0 | 0 |
| PR4046 | Snyder | 67 | Female | M1a | NA | NA | Ipilimumab | 139 | 4.16 | 105 | NA | 14.9 | Deceased | NA | NA | DCB | 1.234 | 1 | 0 | 0 | 0 |
| PR4077 | Snyder | 77 | Male | M1c | NA | NA | Ipilimumab | 1741 | 52.13 | 899 | NA | 12.1 | Deceased | NA | NA | DCB | 5.458 | 1 | 1 | 1 | 1 |
| PR4091 | Snyder | 50 | Male | M1a | NA | NA | Ipilimumab+vemurafenib | 277 | 8.29 | 178 | NA | 2.5 | Deceased | NA | NA | DCB | 2.182 | 1 | 0 | 1 | 0 |
| PR4092 | Snyder | 64 | Female | M1a | NA | NA | Ipilimumab | 3267 | 97.81 | 2611 | NA | 7.2 | Deceased | NA | NA | DCB | 1.64 | 0 | 1 | 0 | 0 |
| SD0346 | Snyder | 74 | Male | M1b | Cutaneous | Normal | Ipilimumab | 537 | 16.08 | 459 | NA | 32.4 | Deceased | NA | NA | DCB | 2.182 | 1 | 0 | 1 | 0 |
| SD1494 | Snyder | 63 | Female | M1c | Cutaneous | Elevated | Ipilimumab | 915 | 27.40 | 492 | NA | 14.4 | Deceased | NA | NA | DCB | 1.636 | 0 | 0 | 0 | 1 |
| SD2051 | Snyder | 38 | Female | M1c | Acral | Elevated | Ipilimumab | 38 | 1.14 | 4 | NA | 25.0 | Deceased | NA | NA | NCB | 0 | 0 | 0 | 0 | 0 |
| SD2056 | Snyder | 90 | Male | M1b | Cutaneous | Normal | Tremelimumab | 136 | 4.07 | 42 | NA | 94.6 | Deceased | NA | NA | DCB | 0 | 0 | 0 | 0 | 0 |
| SD5038 | Snyder | 61 | Female | M1c | Cutaneous | Normal | Ipilimumab+vemurafenib | 256 | 7.66 | 305 | NA | 10.2 | Deceased | NA | NA | NCB | 1.64 | 0 | 1 | 0 | 0 |
| SD5118 | Snyder | 55 | Male | M1c | Acral | Normal | Ipilimumab | 74 | 2.22 | 30 | NA | 14.0 | Deceased | NA | NA | NCB | 0 | 0 | 0 | 0 | 0 |
| SD5934 | Snyder | 55 | Female | M1c | Cutaneous | Normal | Ipilimumab | 156 | 4.67 | 10 | NA | 32.8 | Deceased | NA | NA | NCB | 0 | 0 | 0 | 0 | 0 |
| SD6336 | Snyder | 52 | Male | M1c | Cutaneous | NA | Ipilimumab | 338 | 10.12 | 174 | NA | 19.8 | Deceased | NA | NA | DCB | 1.64 | 0 | 1 | 0 | 0 |
| SD6494 | Snyder | 63 | Female | M1c | Occult | Elevated | Ipilimumab | 594 | 17.78 | 540 | NA | 8.7 | Deceased | NA | NA | NCB | 0 | 0 | 0 | 0 | 0 |
| SD7357 | Snyder | 50 | Female | M1c | Occult | Normal | Ipilimumab | 1148 | 34.37 | 1515 | NA | 24.5 | Deceased | NA | NA | NCB | 1.234 | 1 | 0 | 0 | 0 |
| Patient106 | Liu | NA | Female | M1c | Cutaneous | Elevated | Pembrolizumab | 245 | 6.36 | 726 | CR | 26.0 | Living | 26.0 | Living | DCB | 1.636 | 0 | 0 | 0 | 1 |
| Patient120 | Liu | NA | Female | M1c | Cutaneous | Normal | Pembrolizumab | 1090 | 28.31 | 3786 | CR | 43.7 | Living | 43.7 | Living | DCB | 1.64 | 0 | 1 | 0 | 0 |
| Patient125 | Liu | NA | Male | M1c | Cutaneous | Normal | Nivolumab | 57 | 1.48 | 92 | CR | 32.6 | Living | 32.6 | Living | DCB | 0.948 | 0 | 0 | 1 | 0 |
| Patient127 | Liu | NA | Male | M1c | Occult | Normal | Nivolumab | 414 | 10.75 | 770 | CR | 28.9 | Living | 28.9 | Living | DCB | 0.948 | 0 | 0 | 1 | 0 |
| Patient132 | Liu | NA | Male | M1b | Cutaneous | Normal | Pembrolizumab | 630 | 16.36 | 2078 | CR | 24.6 | Living | 13.0 | Progressed | DCB | 0 | 0 | 0 | 0 | 0 |
| Patient135 | Liu | NA | Male | M1c | Cutaneous | Elevated | Pembrolizumab | 388 | 10.08 | 1137 | CR | 20.9 | Living | 20.9 | Living | DCB | 1.636 | 0 | 0 | 0 | 1 |
| Patient137 | Liu | NA | Female | M1c | Occult | Normal | Pembrolizumab | 860 | 22.34 | 2250 | CR | 25.7 | Living | 25.7 | Living | DCB | 1.234 | 1 | 0 | 0 | 0 |
| Patient145 | Liu | NA | Male | M1c | Cutaneous | Elevated | Pembrolizumab | 288 | 7.48 | 1033 | CR | 27.8 | Living | 27.8 | Living | DCB | 1.636 | 0 | 0 | 0 | 1 |
| Patient146 | Liu | NA | Female | M1c | Cutaneous | Elevated | Pembrolizumab | 375 | 9.74 | 1173 | CR | 20.4 | Living | 20.4 | Living | DCB | 2.874 | 1 | 1 | 0 | 0 |
| Patient147 | Liu | NA | Male | M1c | Occult | Elevated | Nivolumab | 542 | 14.08 | 1540 | CR | 32.0 | Living | 32.0 | Living | DCB | 0 | 0 | 0 | 0 | 0 |
| Patient181 | Liu | NA | Female | M1c | Cutaneous | Elevated | Nivolumab | 275 | 7.14 | 757 | CR | 15.9 | Living | 15.9 | Living | DCB | 0 | 0 | 0 | 0 | 0 |
| Patient184 | Liu | NA | Male | M1c | Cutaneous | Normal | Nivolumab | 1983 | 51.51 | 6173 | CR | 17.1 | Living | 12.4 | Living | DCB | 5.458 | 1 | 1 | 1 | 1 |
| Patient187 | Liu | NA | Female | M1a | Cutaneous | Normal | Pembrolizumab | 133 | 3.45 | 328 | CR | 20.6 | Living | 20.6 | Living | DCB | 0 | 0 | 0 | 0 | 0 |
| Patient204 | Liu | NA | Male | M1a | Occult | Normal | Pembrolizumab | 1772 | 46.03 | 5582 | CR | 27.3 | Living | 21.0 | Living | DCB | 2.87 | 1 | 0 | 0 | 1 |
| Patient4 | Liu | NA | Male | M1c | Cutaneous | Normal | Pembrolizumab | 384 | 9.97 | 1321 | CR | 20.0 | Deceased | 19.1 | Progressed | DCB | 1.64 | 0 | 1 | 0 | 0 |
| Patient42 | Liu | NA | Male | M1c | Cutaneous | Elevated | Nivolumab | 507 | 13.17 | 1610 | CR | 34.2 | Living | 34.2 | Living | DCB | 0 | 0 | 0 | 0 | 0 |
| Patient96 | Liu | NA | Male | M1c | Cutaneous | Normal | Nivolumab | 649 | 16.86 | 2136 | CR | 30.4 | Living | 30.4 | Living | DCB | 1.234 | 1 | 0 | 0 | 0 |
| Patient102 | Liu | NA | Male | M1c | Acral | Elevated | Pembrolizumab | 246 | 6.39 | 825 | MR | 26.2 | Living | 5.9 | Progressed | NA | 1.234 | 1 | 0 | 0 | 0 |
| Patient105 | Liu | NA | Female | M1c | Cutaneous | Elevated | Pembrolizumab | 125 | 3.25 | 334 | MR | 17.4 | Deceased | 12.6 | Living | NA | 0 | 0 | 0 | 0 | 0 |
| Patient50 | Liu | NA | Female | M1c | Mucosal | Elevated | Pembrolizumab | 195 | 5.06 | 599 | MR | 5.8 | Deceased | 3.5 | Progressed | NA | 2.874 | 1 | 1 | 0 | 0 |
| Patient54 | Liu | NA | Male | M1c | Cutaneous | Elevated | Pembrolizumab | 288 | 7.48 | 536 | MR | 33.9 | Deceased | 14.3 | Progressed | NA | 1.234 | 1 | 0 | 0 | 0 |
| Patient1 | Liu | NA | Female | M1c | Occult | NA | Pembrolizumab | 22 | 0.57 | 49 | PD | 9.4 | Deceased | 3.1 | Progressed | NCB | 0 | 0 | 0 | 0 | 0 |
| Patient108 | Liu | NA | Male | M1c | Acral | Elevated | Pembrolizumab | 45 | 1.17 | 189 | PD | 5.5 | Deceased | 2.6 | Progressed | NCB | 1.636 | 0 | 0 | 0 | 1 |
| Patient11 | Liu | NA | Female | M1c | Cutaneous | Elevated | Pembrolizumab | 172 | 4.47 | 560 | PD | 5.3 | Deceased | 2.8 | Progressed | NCB | 0 | 0 | 0 | 0 | 0 |
| Patient110 | Liu | NA | Female | M1c | Cutaneous | Elevated | Pembrolizumab | 1007 | 26.16 | 2949 | PD | 16.9 | Living | 3.1 | Progressed | NCB | 2.874 | 1 | 1 | 0 | 0 |
| Patient112 | Liu | NA | Female | M1c | Cutaneous | Normal | Nivolumab | 241 | 6.26 | 947 | PD | 17.4 | Deceased | 3.0 | Progressed | NCB | 1.234 | 1 | 0 | 0 | 0 |
| Patient116 | Liu | NA | Female | M1c | Cutaneous | Elevated | Nivolumab | 230 | 5.97 | 636 | PD | 3.9 | Deceased | 2.1 | Progressed | NCB | 0 | 0 | 0 | 0 | 0 |
| Patient117 | Liu | NA | Female | M1c | Cutaneous | Elevated | Pembrolizumab | 34 | 0.88 | 110 | PD | 9.8 | Deceased | 3.3 | Progressed | NCB | 0 | 0 | 0 | 0 | 0 |
| Patient119 | Liu | NA | Female | M1c | Mucosal | Normal | Pembrolizumab | 80 | 2.08 | 263 | PD | 8.7 | Deceased | 2.5 | Progressed | NCB | 0 | 0 | 0 | 0 | 0 |
| Patient13 | Liu | NA | Male | M1c | Cutaneous | Elevated | Pembrolizumab | 593 | 15.40 | 1733 | PD | 1.9 | Deceased | 1.5 | Progressed | NCB | 2.182 | 1 | 0 | 1 | 0 |
| Patient130 | Liu | NA | Female | M1c | Occult | Elevated | Nivolumab | 281 | 7.30 | 728 | PD | 6.4 | Deceased | 4.1 | Progressed | NCB | 0 | 0 | 0 | 0 | 0 |
| Patient133 | Liu | NA | Male | M1c | Cutaneous | Elevated | Pembrolizumab | 341 | 8.86 | 999 | PD | 12.3 | Deceased | 2.1 | Progressed | NCB | 0.948 | 0 | 0 | 1 | 0 |
| Patient134 | Liu | NA | Male | M1b | Cutaneous | Normal | Pembrolizumab | 328 | 8.52 | 1029 | PD | 19.7 | Deceased | 2.1 | Progressed | NCB | 0 | 0 | 0 | 0 | 0 |
| Patient136 | Liu | NA | Male | M1b | Cutaneous | Normal | Pembrolizumab | 577 | 14.99 | 1895 | PD | 23.3 | Living | 2.1 | Progressed | NCB | 0 | 0 | 0 | 0 | 0 |
| Patient14 | Liu | NA | Female | M1b | Acral | Normal | Pembrolizumab | 30 | 0.78 | 52 | PD | 6.2 | Deceased | 2.5 | Progressed | NCB | 0 | 0 | 0 | 0 | 0 |
| Patient140 | Liu | NA | Male | M1b | Cutaneous | Normal | Pembrolizumab | 2077 | 53.95 | 6997 | PD | 11.7 | Deceased | 2.2 | Progressed | NCB | 0 | 0 | 0 | 0 | 0 |
| Patient143 | Liu | NA | Male | M0 | Cutaneous | Elevated | Pembrolizumab | 334 | 8.68 | 1163 | PD | 21.0 | Living | 5.1 | Progressed | NCB | 0 | 0 | 0 | 0 | 0 |
| Patient155 | Liu | NA | Male | M0 | Cutaneous | Normal | Pembrolizumab | 255 | 6.62 | 784 | PD | 15.8 | Living | 3.8 | Progressed | NCB | 0 | 0 | 0 | 0 | 0 |
| Patient156 | Liu | NA | Male | M1c | Cutaneous | Elevated | Pembrolizumab | 202 | 5.25 | 630 | PD | 2.9 | Deceased | 2.7 | Progressed | NCB | 1.234 | 1 | 0 | 0 | 0 |
| Patient157 | Liu | NA | Male | M1c | Cutaneous | Normal | Pembrolizumab | 443 | 11.51 | 1074 | PD | 4.8 | Living | 2.8 | Progressed | NCB | 0 | 0 | 0 | 0 | 0 |
| Patient162 | Liu | NA | Male | M1c | Occult | Elevated | Nivolumab | 1566 | 40.68 | 4779 | PD | 6.7 | Deceased | 1.8 | Progressed | NCB | 3.822 | 1 | 1 | 1 | 0 |
| Patient163 | Liu | NA | Male | M1c | Occult | Elevated | Nivolumab | 201 | 5.22 | 470 | PD | 5.8 | Deceased | 1.6 | Progressed | NCB | 0 | 0 | 0 | 0 | 0 |
| Patient165 | Liu | NA | Male | M1c | Cutaneous | Elevated | Pembrolizumab | 641 | 16.65 | 2015 | PD | 2.5 | Living | 0.4 | Progressed | NCB | 2.182 | 1 | 0 | 1 | 0 |
| Patient167 | Liu | NA | Female | M1c | Occult | Elevated | Nivolumab | 42 | 1.09 | 73 | PD | 9.4 | Deceased | 1.9 | Progressed | NCB | 0 | 0 | 0 | 0 | 0 |
| Patient173 | Liu | NA | Female | M1c | Cutaneous | Normal | Pembrolizumab | 72 | 1.87 | 212 | PD | 1.4 | Deceased | 0.5 | Progressed | NCB | 0 | 0 | 0 | 0 | 0 |
| Patient18 | Liu | NA | Male | M0 | Cutaneous | Normal | Nivolumab | 342 | 8.88 | 983 | PD | 4.3 | Deceased | 2.9 | Progressed | NCB | 1.234 | 1 | 0 | 0 | 0 |
| Patient182 | Liu | NA | Female | M1c | Cutaneous | Elevated | Nivolumab | 173 | 4.49 | 557 | PD | 24.9 | Deceased | 2.8 | Progressed | NCB | 0 | 0 | 0 | 0 | 0 |
| Patient183 | Liu | NA | Male | M1c | Cutaneous | Normal | Pembrolizumab | 327 | 8.49 | 938 | PD | 19.1 | Deceased | 2.8 | Progressed | NCB | 0 | 0 | 0 | 0 | 0 |
| Patient188 | Liu | NA | Male | M1a | Acral | Normal | Pembrolizumab | 22 | 0.57 | 54 | PD | 2.5 | Deceased | 0.8 | Progressed | NCB | 0 | 0 | 0 | 0 | 0 |
| Patient196 | Liu | NA | Male | M1c | Occult | Normal | Nivolumab | 1734 | 45.04 | 5696 | PD | 2.7 | Deceased | 2.7 | Progressed | NCB | 0 | 0 | 0 | 0 | 0 |
| Patient20 | Liu | NA | Female | M1c | Cutaneous | Normal | Nivolumab | 394 | 10.23 | 1216 | PD | 8.1 | Deceased | 2.8 | Progressed | NCB | 0 | 0 | 0 | 0 | 0 |
| Patient200 | Liu | NA | Male | M1c | Cutaneous | Elevated | Pembrolizumab | 735 | 19.09 | 1897 | PD | 17.1 | Deceased | 3.6 | Progressed | NCB | 1.234 | 1 | 0 | 0 | 0 |
| Patient201 | Liu | NA | Male | M0 | Mucosal | Normal | Pembrolizumab | 58 | 1.51 | 85 | PD | 5.6 | Deceased | 2.3 | Progressed | NCB | 0 | 0 | 0 | 0 | 0 |
| Patient203 | Liu | NA | Male | M1b | Acral | Normal | Pembrolizumab | 72 | 1.87 | 141 | PD | 20.9 | Deceased | 3.3 | Progressed | NCB | 0 | 0 | 0 | 0 | 0 |
| Patient205 | Liu | NA | Female | M1c | Cutaneous | Elevated | Pembrolizumab | 572 | 14.86 | 1702 | PD | 13.1 | Deceased | 6.3 | Progressed | NCB | 2.182 | 1 | 0 | 1 | 0 |
| Patient206 | Liu | NA | Male | M1c | Cutaneous | Normal | Pembrolizumab | 5426 | 140.94 | 17933 | PD | 3.0 | Deceased | 1.4 | Progressed | NCB | 3.822 | 1 | 1 | 1 | 0 |
| Patient22 | Liu | NA | Male | M1c | Acral | Elevated | Nivolumab | 58 | 1.51 | 203 | PD | 8.4 | Deceased | 3.0 | Progressed | NCB | 1.234 | 1 | 0 | 0 | 0 |
| Patient23 | Liu | NA | Male | M0 | Cutaneous | NA | Nivolumab | 132 | 3.43 | 450 | PD | 32.5 | Living | 2.6 | Progressed | NCB | 1.234 | 1 | 0 | 0 | 0 |
| Patient24 | Liu | NA | Male | M1c | Occult | Elevated | Nivolumab | 276 | 7.17 | 837 | PD | 4.6 | Deceased | 1.0 | Progressed | NCB | 0 | 0 | 0 | 0 | 0 |
| Patient27 | Liu | NA | Male | M1a | Cutaneous | Normal | Pembrolizumab | 217 | 5.64 | 717 | PD | 14.4 | Deceased | 3.1 | Progressed | NCB | 1.64 | 0 | 1 | 0 | 0 |
| Patient31 | Liu | NA | Male | M1c | Cutaneous | NA | Pembrolizumab | 167 | 4.34 | 372 | PD | 14.2 | Deceased | 2.8 | Progressed | NCB | 0 | 0 | 0 | 0 | 0 |
| Patient32 | Liu | NA | Female | M1c | Cutaneous | Elevated | Pembrolizumab | 270 | 7.01 | 769 | PD | 1.8 | Deceased | 1.6 | Progressed | NCB | 0 | 0 | 0 | 0 | 0 |
| Patient36 | Liu | NA | Male | M1c | Cutaneous | Elevated | Nivolumab | 180 | 4.68 | 543 | PD | 15.0 | Deceased | 2.1 | Progressed | NCB | 0 | 0 | 0 | 0 | 0 |
| Patient37 | Liu | NA | Male | M1c | Mucosal | Elevated | Nivolumab | 40 | 1.04 | 60 | PD | 9.2 | Deceased | 2.6 | Progressed | NCB | 0 | 0 | 0 | 0 | 0 |
| Patient38 | Liu | NA | Male | M1c | Cutaneous | Elevated | Nivolumab | 39 | 1.01 | 85 | PD | 11.4 | Deceased | 3.3 | Progressed | NCB | 0 | 0 | 0 | 0 | 0 |
| Patient40 | Liu | NA | Female | M1c | Cutaneous | Elevated | Nivolumab | 6103 | 158.52 | 13972 | PD | 15.6 | Deceased | 2.1 | Progressed | NCB | 4.51 | 1 | 1 | 0 | 1 |
| Patient47 | Liu | NA | Female | M1c | Cutaneous | Elevated | Nivolumab | 756 | 19.64 | 2511 | PD | 31.2 | Living | 2.5 | Progressed | NCB | 1.64 | 0 | 1 | 0 | 0 |
| Patient48 | Liu | NA | Female | M1c | Occult | Elevated | Pembrolizumab | 29 | 0.75 | 87 | PD | 1.8 | Deceased | 1.7 | Progressed | NCB | 0 | 0 | 0 | 0 | 0 |
| Patient56 | Liu | NA | Male | M1c | Cutaneous | Elevated | Nivolumab | 17 | 0.44 | 42 | PD | 4.1 | Deceased | 2.4 | Progressed | NCB | 0 | 0 | 0 | 0 | 0 |
| Patient58 | Liu | NA | Male | M1c | Cutaneous | Elevated | Nivolumab | 91 | 2.36 | 303 | PD | 10.1 | Deceased | 2.6 | Progressed | NCB | 0 | 0 | 0 | 0 | 0 |
| Patient59 | Liu | NA | Male | M1c | Cutaneous | Elevated | Nivolumab | 315 | 8.18 | 1025 | PD | 6.4 | Deceased | 2.7 | Progressed | NCB | 0 | 0 | 0 | 0 | 0 |
| Patient63 | Liu | NA | Female | M1c | Mucosal | Elevated | Nivolumab | 84 | 2.18 | 188 | PD | 5.9 | Deceased | 3.0 | Progressed | NCB | 0 | 0 | 0 | 0 | 0 |
| Patient67 | Liu | NA | Female | M1c | Mucosal | Normal | Nivolumab | 74 | 1.92 | 281 | PD | 4.5 | Deceased | 3.1 | Progressed | NCB | 0 | 0 | 0 | 0 | 0 |
| Patient72 | Liu | NA | Male | M1a | Cutaneous | Normal | Nivolumab | 121 | 3.14 | 371 | PD | 13.2 | Living | 2.2 | Progressed | NCB | 2.874 | 1 | 1 | 0 | 0 |
| Patient73 | Liu | NA | Male | M1c | Cutaneous | Elevated | Nivolumab | 90 | 2.34 | 262 | PD | 28.2 | Deceased | 2.8 | Progressed | NCB | 0 | 0 | 0 | 0 | 0 |
| Patient77 | Liu | NA | Female | M1c | Cutaneous | Normal | Pembrolizumab | 92 | 2.39 | 201 | PD | 14.8 | Deceased | 3.2 | Progressed | NCB | 0 | 0 | 0 | 0 | 0 |
| Patient78 | Liu | NA | Female | M1b | Cutaneous | Normal | Pembrolizumab | 43 | 1.12 | 123 | PD | 11.9 | Deceased | 2.9 | Progressed | NCB | 0 | 0 | 0 | 0 | 0 |
| Patient79 | Liu | NA | Female | M1c | Cutaneous | Normal | Pembrolizumab | 205 | 5.32 | 591 | PD | 28.3 | Living | 2.8 | Progressed | NCB | 0 | 0 | 0 | 0 | 0 |
| Patient80 | Liu | NA | Male | M1b | Acral | Normal | Pembrolizumab | 33 | 0.86 | 104 | PD | 16.7 | Deceased | 2.6 | Progressed | NCB | 0 | 0 | 0 | 0 | 0 |
| Patient82 | Liu | NA | Male | M1c | Occult | Elevated | Pembrolizumab | 1477 | 38.36 | 4650 | PD | 4.4 | Deceased | 3.0 | Progressed | NCB | 3.276 | 0 | 1 | 0 | 1 |
| Patient83 | Liu | NA | Male | M1c | Cutaneous | Elevated | Nivolumab | 194 | 5.04 | 539 | PD | 2.4 | Deceased | 2.4 | Progressed | NCB | 0 | 0 | 0 | 0 | 0 |
| Patient84 | Liu | NA | Female | M1c | Mucosal | Normal | Pembrolizumab | 47 | 1.22 | 134 | PD | 5.5 | Deceased | 2.8 | Progressed | NCB | 0 | 0 | 0 | 0 | 0 |
| Patient88 | Liu | NA | Male | M1c | Acral | Elevated | Pembrolizumab | 37 | 0.96 | 131 | PD | 2.5 | Deceased | 2.3 | Progressed | NCB | 0 | 0 | 0 | 0 | 0 |
| Patient9 | Liu | NA | Female | M0 | Cutaneous | Normal | Nivolumab | 652 | 16.94 | 1996 | PD | 32.1 | Living | 0.7 | Progressed | NCB | 3.276 | 0 | 1 | 0 | 1 |
| Patient94 | Liu | NA | Female | M1c | Cutaneous | Elevated | Pembrolizumab | 268 | 6.96 | 929 | PD | 1.3 | Deceased | 1.3 | Progressed | NCB | 0 | 0 | 0 | 0 | 0 |
| Patient98 | Liu | NA | Female | M1b | Occult | Normal | Nivolumab | 302 | 7.84 | 790 | PD | 22.8 | Deceased | 2.4 | Progressed | NCB | 2.874 | 1 | 1 | 0 | 0 |
| Patient10 | Liu | NA | Female | M1c | Cutaneous | Elevated | Pembrolizumab | 71 | 1.84 | 230 | PR | 38.0 | Deceased | 5.6 | Progressed | DCB | 0 | 0 | 0 | 0 | 0 |
| Patient100 | Liu | NA | Female | M1c | Occult | Elevated | Nivolumab | 126 | 3.27 | 301 | PR | 31.3 | Deceased | 30.7 | Living | DCB | 0 | 0 | 0 | 0 | 0 |
| Patient104 | Liu | NA | Female | M1c | Mucosal | Elevated | Pembrolizumab | 96 | 2.49 | 329 | PR | 26.3 | Living | 26.3 | Living | DCB | 1.636 | 0 | 0 | 0 | 1 |
| Patient107 | Liu | NA | Male | M1c | Cutaneous | Elevated | Pembrolizumab | 158 | 4.10 | 489 | PR | 1.6 | Deceased | 1.6 | Progressed | DCB | 0 | 0 | 0 | 0 | 0 |
| Patient118 | Liu | NA | Female | M1c | Cutaneous | Normal | Pembrolizumab | 221 | 5.74 | 729 | PR | 43.9 | Living | 43.9 | Living | DCB | 1.64 | 0 | 1 | 0 | 0 |
| Patient121 | Liu | NA | Female | M1c | Cutaneous | Normal | Pembrolizumab | 767 | 19.92 | 2257 | PR | 43.5 | Living | 40.9 | Progressed | DCB | 1.234 | 1 | 0 | 0 | 0 |
| Patient126 | Liu | NA | Male | M1c | Occult | Normal | Nivolumab | 656 | 17.04 | 1862 | PR | 33.1 | Living | 24.7 | Progressed | DCB | 1.636 | 0 | 0 | 0 | 1 |
| Patient131 | Liu | NA | Male | M1b | Cutaneous | Normal | Pembrolizumab | 311 | 8.08 | 790 | PR | 27.8 | Living | 27.8 | Living | DCB | 0 | 0 | 0 | 0 | 0 |
| Patient141 | Liu | NA | Female | M1c | Cutaneous | Elevated | Nivolumab | 60 | 1.56 | 194 | PR | 29.2 | Living | 29.2 | Living | DCB | 0 | 0 | 0 | 0 | 0 |
| Patient144 | Liu | NA | Male | M1c | Cutaneous | Elevated | Pembrolizumab | 824 | 21.40 | 2594 | PR | 22.1 | Living | 22.1 | Living | DCB | 2.182 | 1 | 0 | 1 | 0 |
| Patient148 | Liu | NA | Female | M1a | Cutaneous | Normal | Pembrolizumab | 320 | 8.31 | 1103 | PR | 26.4 | Living | 26.4 | Living | DCB | 1.234 | 1 | 0 | 0 | 0 |
| Patient15 | Liu | NA | Female | M1c | Cutaneous | Normal | Pembrolizumab | 182 | 4.73 | 530 | PR | 5.0 | Deceased | 3.5 | Progressed | DCB | 0 | 0 | 0 | 0 | 0 |
| Patient150 | Liu | NA | Male | M1c | Cutaneous | Normal | Pembrolizumab | 23 | 0.60 | 41 | PR | 22.8 | Living | 22.8 | Living | DCB | 0 | 0 | 0 | 0 | 0 |
| Patient158 | Liu | NA | Male | M1c | Cutaneous | Elevated | Pembrolizumab | 2296 | 59.64 | 6776 | PR | 6.5 | Living | 5.4 | Progressed | DCB | 4.51 | 1 | 1 | 0 | 1 |
| Patient166 | Liu | NA | Female | M1c | Cutaneous | Elevated | Nivolumab | 10 | 0.26 | 20 | PR | 6.8 | Deceased | 6.8 | Living | DCB | 0 | 0 | 0 | 0 | 0 |
| Patient168 | Liu | NA | Male | M1c | Cutaneous | Elevated | Nivolumab | 31 | 0.81 | 94 | PR | 20.8 | Living | 19.3 | Living | DCB | 0 | 0 | 0 | 0 | 0 |
| Patient169 | Liu | NA | Male | M1c | Cutaneous | Elevated | Pembrolizumab | 1382 | 35.90 | 4545 | PR | 18.1 | Living | 18.1 | Living | DCB | 5.458 | 1 | 1 | 1 | 1 |
| Patient179 | Liu | NA | Female | M1b | Cutaneous | Normal | Pembrolizumab | 478 | 12.42 | 1485 | PR | 21.8 | Living | 11.2 | Progressed | DCB | 2.182 | 1 | 0 | 1 | 0 |
| Patient189 | Liu | NA | Female | M1c | Acral | Elevated | Pembrolizumab | 50 | 1.30 | 134 | PR | 23.9 | Living | 13.5 | Progressed | DCB | 0 | 0 | 0 | 0 | 0 |
| Patient191 | Liu | NA | Male | M1b | Cutaneous | Normal | Nivolumab | 4234 | 109.97 | 10277 | PR | 21.9 | Living | 21.9 | Living | DCB | 1.234 | 1 | 0 | 0 | 0 |
| Patient192 | Liu | NA | Female | M1c | Cutaneous | Elevated | Pembrolizumab | 461 | 11.97 | 1419 | PR | 9.7 | Living | 8.3 | Progressed | DCB | 1.636 | 0 | 0 | 0 | 1 |
| Patient195 | Liu | NA | Male | M1c | Cutaneous | Normal | Nivolumab | 1963 | 50.99 | 5347 | PR | 29.8 | Living | 29.8 | Living | DCB | 4.51 | 1 | 1 | 0 | 1 |
| Patient197 | Liu | NA | Female | M1c | Cutaneous | Elevated | Nivolumab | 59 | 1.53 | 185 | PR | 32.0 | Living | 3.0 | Progressed | DCB | 0 | 0 | 0 | 0 | 0 |
| Patient21 | Liu | NA | Female | M1c | Acral | Normal | Pembrolizumab | 105 | 2.73 | 184 | PR | 30.2 | Living | 30.2 | Living | DCB | 0 | 0 | 0 | 0 | 0 |
| Patient25 | Liu | NA | Male | M1c | Cutaneous | Elevated | Pembrolizumab | 328 | 8.52 | 1088 | PR | 36.5 | Living | 25.1 | Progressed | DCB | 0 | 0 | 0 | 0 | 0 |
| Patient33 | Liu | NA | Female | M1c | Cutaneous | Normal | Pembrolizumab | 10 | 0.26 | 35 | PR | 56.0 | Living | 56.0 | Living | DCB | 0 | 0 | 0 | 0 | 0 |
| Patient34 | Liu | NA | Male | M1b | Cutaneous | Normal | Pembrolizumab | 18 | 0.47 | 24 | PR | 56.4 | Living | 8.0 | Progressed | DCB | 0 | 0 | 0 | 0 | 0 |
| Patient41 | Liu | NA | Male | M1b | Cutaneous | Normal | Nivolumab | 9835 | 255.45 | 24622 | PR | 56.2 | Living | 3.5 | Progressed | DCB | 5.458 | 1 | 1 | 1 | 1 |
| Patient43 | Liu | NA | Male | M1c | Cutaneous | Elevated | Nivolumab | 84 | 2.18 | 291 | PR | 30.8 | Living | 25.0 | Progressed | DCB | 0 | 0 | 0 | 0 | 0 |
| Patient44 | Liu | NA | Male | M1c | Mucosal | Elevated | Nivolumab | 64 | 1.66 | 101 | PR | 30.7 | Living | 30.7 | Living | DCB | 0 | 0 | 0 | 0 | 0 |
| Patient45 | Liu | NA | Male | M1c | Cutaneous | Elevated | Nivolumab | 293 | 7.61 | 793 | PR | 55.5 | Living | 55.5 | Living | DCB | 0 | 0 | 0 | 0 | 0 |
| Patient49 | Liu | NA | Female | M1c | Cutaneous | Normal | Nivolumab | 1624 | 42.18 | 4836 | PR | 54.5 | Living | 9.3 | Progressed | DCB | 0 | 0 | 0 | 0 | 0 |
| Patient51 | Liu | NA | Male | M1b | Cutaneous | Normal | Nivolumab | 1134 | 29.45 | 3069 | PR | 51.6 | Living | 15.7 | Progressed | DCB | 1.636 | 0 | 0 | 0 | 1 |
| Patient55 | Liu | NA | Male | M1c | Cutaneous | Normal | Nivolumab | 478 | 12.42 | 1421 | PR | 50.1 | Living | 50.1 | Living | DCB | 1.234 | 1 | 0 | 0 | 0 |
| Patient62 | Liu | NA | Female | M1c | Cutaneous | Normal | Nivolumab | 6249 | 162.31 | 18457 | PR | 33.4 | Living | 33.4 | Living | DCB | 5.458 | 1 | 1 | 1 | 1 |
| Patient75 | Liu | NA | Male | M1c | Occult | Elevated | Nivolumab | 725 | 18.83 | 2304 | PR | 29.7 | Living | 29.7 | Progressed | DCB | 1.234 | 1 | 0 | 0 | 0 |
| Patient87 | Liu | NA | Female | M1c | Occult | Elevated | Pembrolizumab | 87 | 2.26 | 222 | PR | 42.1 | Living | 38.1 | Progressed | DCB | 0 | 0 | 0 | 0 | 0 |
| Patient99 | Liu | NA | Male | M1c | Cutaneous | Elevated | Nivolumab | 909 | 23.61 | 2796 | PR | 30.7 | Living | 10.6 | Progressed | DCB | 1.234 | 1 | 0 | 0 | 0 |
| Patient30 | Liu | NA | Female | M1c | Cutaneous | Elevated | Pembrolizumab | 158 | 4.10 | 481 | SD | 7.1 | Deceased | 4.4 | Progressed | NCB | 0.948 | 0 | 0 | 1 | 0 |
| Patient6 | Liu | NA | Female | M1b | Cutaneous | Normal | Pembrolizumab | 935 | 24.29 | 2041 | SD | 22.9 | Deceased | 4.1 | Progressed | NCB | 0 | 0 | 0 | 0 | 0 |
| Patient60 | Liu | NA | Male | M1b | Cutaneous | Normal | Nivolumab | 392 | 10.18 | 1106 | SD | 22.1 | Deceased | 5.8 | Progressed | NCB | 1.636 | 0 | 0 | 0 | 1 |
| Patient142 | Liu | NA | Female | M1a | Cutaneous | Normal | Pembrolizumab | 48 | 1.25 | 198 | SD | 33.2 | Living | 33.2 | Living | DCB | 0 | 0 | 0 | 0 | 0 |
| Patient149 | Liu | NA | Male | M1c | Cutaneous | Normal | Pembrolizumab | 219 | 5.69 | 690 | SD | 16.6 | Deceased | 7.0 | Progressed | DCB | 0 | 0 | 0 | 0 | 0 |
| Patient154 | Liu | NA | Female | M0 | Cutaneous | Normal | Pembrolizumab | 72 | 1.87 | 216 | SD | 21.2 | Living | 8.4 | Progressed | DCB | 1.636 | 0 | 0 | 0 | 1 |
| Patient159 | Liu | NA | Female | M1c | Mucosal | Normal | Pembrolizumab | 46 | 1.19 | 204 | SD | 9.6 | Living | 7.2 | Progressed | DCB | 0 | 0 | 0 | 0 | 0 |
| Patient17 | Liu | NA | Male | M1c | Cutaneous | Normal | Nivolumab | 365 | 9.48 | 821 | SD | 22.9 | Deceased | 6.7 | Progressed | DCB | 1.64 | 0 | 1 | 0 | 0 |
| Patient170 | Liu | NA | Male | M0 | Cutaneous | Normal | Pembrolizumab | 56 | 1.45 | 148 | SD | 11.6 | Living | 11.6 | Living | DCB | 0 | 0 | 0 | 0 | 0 |
| Patient172 | Liu | NA | Male | M0 | Cutaneous | Normal | Pembrolizumab | 678 | 17.61 | 2400 | SD | 9.1 | Living | 9.1 | Progressed | DCB | 0.948 | 0 | 0 | 1 | 0 |
| Patient180 | Liu | NA | Male | M1c | Cutaneous | Elevated | Pembrolizumab | 583 | 15.14 | 2146 | SD | 12.7 | Deceased | 11.4 | Progressed | DCB | 0 | 0 | 0 | 0 | 0 |
| Patient185 | Liu | NA | Male | M1c | Cutaneous | Elevated | Nivolumab | 157 | 4.08 | 490 | SD | 6.8 | Living | 6.8 | Living | DCB | 0 | 0 | 0 | 0 | 0 |
| Patient193 | Liu | NA | Female | M0 | Cutaneous | Normal | Pembrolizumab | 571 | 14.83 | 1570 | SD | 22.0 | Living | 15.9 | Progressed | DCB | 0 | 0 | 0 | 0 | 0 |
| Patient35 | Liu | NA | Male | M1c | Cutaneous | Normal | Nivolumab | 40 | 1.04 | 112 | SD | 32.3 | Deceased | 6.0 | Progressed | DCB | 0 | 0 | 0 | 0 | 0 |
| Patient39 | Liu | NA | Male | M1a | Cutaneous | Normal | Nivolumab | 11 | 0.29 | 24 | SD | 31.2 | Living | 31.2 | Living | DCB | 0 | 0 | 0 | 0 | 0 |
| Patient46 | Liu | NA | Male | M1c | Cutaneous | Elevated | Pembrolizumab | 833 | 21.64 | 2022 | SD | 29.9 | Living | 29.9 | Living | DCB | 0.948 | 0 | 0 | 1 | 0 |
| Patient61 | Liu | NA | Female | M1c | Cutaneous | Normal | Nivolumab | 2117 | 54.99 | 5854 | SD | 32.9 | Living | 32.9 | Living | DCB | 1.234 | 1 | 0 | 0 | 0 |
| Patient7 | Liu | NA | Male | M1c | Occult | Elevated | Pembrolizumab | 79 | 2.05 | 138 | SD | 9.0 | Deceased | 7.2 | Progressed | DCB | 0 | 0 | 0 | 0 | 0 |
| Patient8 | Liu | NA | Male | M1b | Cutaneous | Normal | Pembrolizumab | 194 | 5.04 | 638 | SD | 33.5 | Deceased | 31.8 | Progressed | DCB | 1.234 | 1 | 0 | 0 | 0 |
| Patient86 | Liu | NA | Male | M1c | Cutaneous | Normal | Pembrolizumab | 279 | 7.25 | 576 | SD | 44.2 | Living | 44.2 | Progressed | DCB | 1.636 | 0 | 0 | 0 | 1 |
