# Supplementary material for: Integrated genomic analysis identifies a genetic mutation model predicting response to immune checkpoint inhibitors in melanoma
Source: Cancer Med. 2020 Sep 24;9(22):8498–518. doi: 10.1002/cam4.3481 (PMC7666739; doi:10.1002/cam4.3481)
Supplement: Supplementary file 10 — Table S2 [file CAM4-9-8498-s010.docx]

| **Table S2. The detailed clinical and genomic characteristics of melanoma patients in the TCGA-SKCM and ICGC-MELA cohort** | | | | | | | | |
| --- | --- | --- | --- | --- | --- | --- | --- | --- |
| ID | Cohort | OS time (months) | OS status | ITS | *THSD7B* | *SYNE2* | *GRM3* | *FLNC* |
| TCGA-ER-A2NF-06 | TCGA-SKCM | NA | NA | 0 | 0 | 0 | 0 | 0 |
| TCGA-HR-A2OG-01 | TCGA-SKCM | NA | NA | 0 | 0 | 0 | 0 | 0 |
| TCGA-D9-A1X3-01 | TCGA-SKCM | NA | NA | 0 | 0 | 0 | 0 | 0 |
| TCGA-ER-A19T-06 | TCGA-SKCM | NA | NA | 0 | 0 | 0 | 0 | 0 |
| TCGA-HR-A2OH-01 | TCGA-SKCM | NA | NA | 0.948 | 0 | 0 | 1 | 0 |
| TCGA-D9-A4Z6-01 | TCGA-SKCM | NA | NA | 1.234 | 1 | 0 | 0 | 0 |
| TCGA-D3-A3C1-06 | TCGA-SKCM | NA | Living | 0 | 0 | 0 | 0 | 0 |
| TCGA-D3-A3C3-06 | TCGA-SKCM | NA | Living | 0 | 0 | 0 | 0 | 0 |
| TCGA-D3-A51G-06 | TCGA-SKCM | NA | Living | 0.948 | 0 | 0 | 1 | 0 |
| TCGA-RP-A695-06 | TCGA-SKCM | NA | Living | 1.234 | 1 | 0 | 0 | 0 |
| TCGA-FR-A3YO-06 | TCGA-SKCM | NA | Living | 2.87 | 1 | 0 | 0 | 1 |
| TCGA-EB-A430-01 | TCGA-SKCM | NA | Living | 2.584 | 0 | 0 | 1 | 1 |
| TCGA-RP-A690-06 | TCGA-SKCM | 0.2 | Living | 0 | 0 | 0 | 0 | 0 |
| TCGA-RP-A693-06 | TCGA-SKCM | 0.3 | Living | 1.234 | 1 | 0 | 0 | 0 |
| TCGA-BF-A1PV-01 | TCGA-SKCM | 0.5 | Living | 1.234 | 1 | 0 | 0 | 0 |
| TCGA-GF-A2C7-01 | TCGA-SKCM | 0.7 | Living | 0 | 0 | 0 | 0 | 0 |
| TCGA-RP-A694-06 | TCGA-SKCM | 0.7 | Living | 1.234 | 1 | 0 | 0 | 0 |
| TCGA-EB-A3HV-01 | TCGA-SKCM | 1.3 | Living | 0 | 0 | 0 | 0 | 0 |
| TCGA-GF-A6C8-06 | TCGA-SKCM | 2.0 | Living | 1.636 | 0 | 0 | 0 | 1 |
| TCGA-EB-A44O-01 | TCGA-SKCM | 2.7 | Living | 1.234 | 1 | 0 | 0 | 0 |
| TCGA-D9-A1JW-06 | TCGA-SKCM | 3.7 | Living | 2.87 | 1 | 0 | 0 | 1 |
| TCGA-EB-A3Y6-01 | TCGA-SKCM | 4.1 | Living | 1.234 | 1 | 0 | 0 | 0 |
| TCGA-EB-A3XE-01 | TCGA-SKCM | 5.9 | Living | 0 | 0 | 0 | 0 | 0 |
| TCGA-EB-A5VV-06 | TCGA-SKCM | 7.0 | Living | 0 | 0 | 0 | 0 | 0 |
| TCGA-D9-A4Z5-01 | TCGA-SKCM | 7.2 | Living | 0 | 0 | 0 | 0 | 0 |
| TCGA-QB-A6FS-06 | TCGA-SKCM | 7.2 | Living | 2.87 | 1 | 0 | 0 | 1 |
| TCGA-EB-A553-01 | TCGA-SKCM | 7.4 | Living | 0.948 | 0 | 0 | 1 | 0 |
| TCGA-EB-A41A-01 | TCGA-SKCM | 8.6 | Living | 1.636 | 0 | 0 | 0 | 1 |
| TCGA-EB-A3XF-01 | TCGA-SKCM | 9.1 | Living | 2.874 | 1 | 1 | 0 | 0 |
| TCGA-EB-A41B-01 | TCGA-SKCM | 9.6 | Living | 1.234 | 1 | 0 | 0 | 0 |
| TCGA-GF-A3OT-06 | TCGA-SKCM | 9.9 | Living | 0 | 0 | 0 | 0 | 0 |
| TCGA-D9-A6E9-06 | TCGA-SKCM | 9.9 | Living | 0 | 0 | 0 | 0 | 0 |
| TCGA-BF-A5EQ-01 | TCGA-SKCM | 10.6 | Living | 1.636 | 0 | 0 | 0 | 1 |
| TCGA-BF-A5ER-01 | TCGA-SKCM | 10.7 | Living | 0 | 0 | 0 | 0 | 0 |
| TCGA-BF-A5EP-01 | TCGA-SKCM | 11.0 | Living | 0 | 0 | 0 | 0 | 0 |
| TCGA-EB-A299-01 | TCGA-SKCM | 12.4 | Living | 0 | 0 | 0 | 0 | 0 |
| TCGA-EB-A6QY-01 | TCGA-SKCM | 12.6 | Living | 1.64 | 0 | 1 | 0 | 0 |
| TCGA-BF-A1PU-01 | TCGA-SKCM | 12.7 | Living | 0 | 0 | 0 | 0 | 0 |
| TCGA-FW-A3TV-06 | TCGA-SKCM | 13.5 | Living | 4.51 | 1 | 1 | 0 | 1 |
| TCGA-EB-A44Q-06 | TCGA-SKCM | 13.9 | Living | 0 | 0 | 0 | 0 | 0 |
| TCGA-EE-A29R-06 | TCGA-SKCM | 14.5 | Living | 0 | 0 | 0 | 0 | 0 |
| TCGA-EB-A42Z-01 | TCGA-SKCM | 14.5 | Living | 0 | 0 | 0 | 0 | 0 |
| TCGA-BF-A3DJ-01 | TCGA-SKCM | 15.2 | Living | 0 | 0 | 0 | 0 | 0 |
| TCGA-FR-A69P-06 | TCGA-SKCM | 15.7 | Living | 0.948 | 0 | 0 | 1 | 0 |
| TCGA-GF-A6C9-06 | TCGA-SKCM | 15.8 | Living | 3.822 | 1 | 1 | 1 | 0 |
| TCGA-D3-A5GT-01 | TCGA-SKCM | 16.0 | Living | 0 | 0 | 0 | 0 | 0 |
| TCGA-BF-A5ES-01 | TCGA-SKCM | 16.1 | Living | 0.948 | 0 | 0 | 1 | 0 |
| TCGA-D9-A4Z3-01 | TCGA-SKCM | 16.6 | Living | 1.234 | 1 | 0 | 0 | 0 |
| TCGA-IH-A3EA-01 | TCGA-SKCM | 17.2 | Living | 1.234 | 1 | 0 | 0 | 0 |
| TCGA-FW-A3I3-06 | TCGA-SKCM | 17.4 | Living | 0 | 0 | 0 | 0 | 0 |
| TCGA-D3-A5GS-06 | TCGA-SKCM | 18.2 | Living | 1.636 | 0 | 0 | 0 | 1 |
| TCGA-EB-A431-01 | TCGA-SKCM | 18.7 | Living | 2.87 | 1 | 0 | 0 | 1 |
| TCGA-FR-A728-01 | TCGA-SKCM | 19.2 | Living | 1.636 | 0 | 0 | 0 | 1 |
| TCGA-FW-A5DY-06 | TCGA-SKCM | 19.3 | Living | 0 | 0 | 0 | 0 | 0 |
| TCGA-EB-A551-01 | TCGA-SKCM | 19.4 | Living | 0 | 0 | 0 | 0 | 0 |
| TCGA-BF-A3DM-01 | TCGA-SKCM | 19.7 | Living | 0.948 | 0 | 0 | 1 | 0 |
| TCGA-EB-A4OZ-01 | TCGA-SKCM | 20.4 | Living | 0 | 0 | 0 | 0 | 0 |
| TCGA-EB-A24C-01 | TCGA-SKCM | 20.8 | Living | 0 | 0 | 0 | 0 | 0 |
| TCGA-FW-A5DX-01 | TCGA-SKCM | 21.0 | Living | 0 | 0 | 0 | 0 | 0 |
| TCGA-EB-A24D-01 | TCGA-SKCM | 21.2 | Living | 0 | 0 | 0 | 0 | 0 |
| TCGA-EB-A3XC-01 | TCGA-SKCM | 21.4 | Living | 1.636 | 0 | 0 | 0 | 1 |
| TCGA-D9-A3Z3-06 | TCGA-SKCM | 22.3 | Living | 0.948 | 0 | 0 | 1 | 0 |
| TCGA-FR-A3R1-01 | TCGA-SKCM | 22.5 | Living | 1.234 | 1 | 0 | 0 | 0 |
| TCGA-D3-A51N-06 | TCGA-SKCM | 22.6 | Living | 0 | 0 | 0 | 0 | 0 |
| TCGA-BF-A5EO-01 | TCGA-SKCM | 23.1 | Living | 1.636 | 0 | 0 | 0 | 1 |
| TCGA-BF-A3DN-01 | TCGA-SKCM | 23.6 | Living | 0 | 0 | 0 | 0 | 0 |
| TCGA-EB-A44P-01 | TCGA-SKCM | 24.3 | Living | 0 | 0 | 0 | 0 | 0 |
| TCGA-D9-A6EA-06 | TCGA-SKCM | 25.2 | Living | 0 | 0 | 0 | 0 | 0 |
| TCGA-BF-A3DL-01 | TCGA-SKCM | 25.3 | Living | 1.234 | 1 | 0 | 0 | 0 |
| TCGA-EB-A4IS-01 | TCGA-SKCM | 25.4 | Living | 2.182 | 1 | 0 | 1 | 0 |
| TCGA-EB-A4XL-01 | TCGA-SKCM | 25.5 | Living | 1.234 | 1 | 0 | 0 | 0 |
| TCGA-EB-A5UM-01 | TCGA-SKCM | 25.6 | Living | 0 | 0 | 0 | 0 | 0 |
| TCGA-EB-A3XB-01 | TCGA-SKCM | 26.2 | Living | 1.234 | 1 | 0 | 0 | 0 |
| TCGA-D3-A51T-06 | TCGA-SKCM | 26.9 | Living | 1.64 | 0 | 1 | 0 | 0 |
| TCGA-BF-A1Q0-01 | TCGA-SKCM | 27.3 | Living | 1.636 | 0 | 0 | 0 | 1 |
| TCGA-BF-A1PZ-01 | TCGA-SKCM | 28.0 | Living | 0 | 0 | 0 | 0 | 0 |
| TCGA-EB-A5UL-06 | TCGA-SKCM | 29.3 | Living | 1.234 | 1 | 0 | 0 | 0 |
| TCGA-EB-A4OY-01 | TCGA-SKCM | 32.1 | Living | 0 | 0 | 0 | 0 | 0 |
| TCGA-D3-A51K-06 | TCGA-SKCM | 32.9 | Living | 0 | 0 | 0 | 0 | 0 |
| TCGA-ER-A3PL-06 | TCGA-SKCM | 33.2 | Living | 0 | 0 | 0 | 0 | 0 |
| TCGA-FS-A4F9-06 | TCGA-SKCM | 34.0 | Living | 0 | 0 | 0 | 0 | 0 |
| TCGA-EB-A1NK-01 | TCGA-SKCM | 34.1 | Living | 0 | 0 | 0 | 0 | 0 |
| TCGA-EB-A6L9-06 | TCGA-SKCM | 36.4 | Living | 0 | 0 | 0 | 0 | 0 |
| TCGA-FW-A3R5-06 | TCGA-SKCM | 36.9 | Living | 5.458 | 1 | 1 | 1 | 1 |
| TCGA-GN-A4U5-01 | TCGA-SKCM | 38.0 | Living | 0 | 0 | 0 | 0 | 0 |
| TCGA-EB-A3XD-01 | TCGA-SKCM | 38.1 | Living | 0 | 0 | 0 | 0 | 0 |
| TCGA-GN-A4U4-06 | TCGA-SKCM | 39.3 | Living | 2.874 | 1 | 1 | 0 | 0 |
| TCGA-D3-A3MU-06 | TCGA-SKCM | 39.7 | Living | 1.234 | 1 | 0 | 0 | 0 |
| TCGA-ER-A2NH-06 | TCGA-SKCM | 41.5 | Living | 1.636 | 0 | 0 | 0 | 1 |
| TCGA-D3-A2JH-06 | TCGA-SKCM | 42.1 | Living | 1.636 | 0 | 0 | 0 | 1 |
| TCGA-DA-A3F8-06 | TCGA-SKCM | 43.3 | Living | 2.182 | 1 | 0 | 1 | 0 |
| TCGA-EE-A2GT-06 | TCGA-SKCM | 44.8 | Living | 0 | 0 | 0 | 0 | 0 |
| TCGA-D3-A3MV-06 | TCGA-SKCM | 45.3 | Living | 2.87 | 1 | 0 | 0 | 1 |
| TCGA-D3-A3C8-06 | TCGA-SKCM | 46.3 | Living | 1.64 | 0 | 1 | 0 | 0 |
| TCGA-D3-A3C7-06 | TCGA-SKCM | 46.9 | Living | 1.234 | 1 | 0 | 0 | 0 |
| TCGA-EE-A2GI-06 | TCGA-SKCM | 48.7 | Living | 1.234 | 1 | 0 | 0 | 0 |
| TCGA-GN-A4U8-06 | TCGA-SKCM | 48.9 | Living | 1.234 | 1 | 0 | 0 | 0 |
| TCGA-ER-A19S-06 | TCGA-SKCM | 49.4 | Living | 0 | 0 | 0 | 0 | 0 |
| TCGA-FS-A1ZW-06 | TCGA-SKCM | 49.4 | Living | 1.234 | 1 | 0 | 0 | 0 |
| TCGA-EE-A3JE-06 | TCGA-SKCM | 51.3 | Living | 0 | 0 | 0 | 0 | 0 |
| TCGA-FS-A1ZT-06 | TCGA-SKCM | 53.1 | Living | 2.182 | 1 | 0 | 1 | 0 |
| TCGA-EE-A2MU-06 | TCGA-SKCM | 53.2 | Living | 1.234 | 1 | 0 | 0 | 0 |
| TCGA-EB-A5SH-06 | TCGA-SKCM | 54.0 | Living | 0 | 0 | 0 | 0 | 0 |
| TCGA-EE-A3AE-06 | TCGA-SKCM | 54.5 | Living | 1.636 | 0 | 0 | 0 | 1 |
| TCGA-D9-A149-06 | TCGA-SKCM | 54.6 | Living | 0 | 0 | 0 | 0 | 0 |
| TCGA-EE-A2GK-06 | TCGA-SKCM | 54.7 | Living | 0 | 0 | 0 | 0 | 0 |
| TCGA-D3-A51F-06 | TCGA-SKCM | 55.7 | Living | 1.64 | 0 | 1 | 0 | 0 |
| TCGA-D3-A51H-06 | TCGA-SKCM | 56.3 | Living | 0 | 0 | 0 | 0 | 0 |
| TCGA-EE-A29P-06 | TCGA-SKCM | 56.4 | Living | 0 | 0 | 0 | 0 | 0 |
| TCGA-EE-A29M-06 | TCGA-SKCM | 56.8 | Living | 2.87 | 1 | 0 | 0 | 1 |
| TCGA-ER-A196-01 | TCGA-SKCM | 58.6 | Living | 0 | 0 | 0 | 0 | 0 |
| TCGA-EB-A5UN-06 | TCGA-SKCM | 58.9 | Living | 0 | 0 | 0 | 0 | 0 |
| TCGA-EE-A2GB-06 | TCGA-SKCM | 59.2 | Living | 1.234 | 1 | 0 | 0 | 0 |
| TCGA-D3-A2JP-06 | TCGA-SKCM | 59.5 | Living | 0 | 0 | 0 | 0 | 0 |
| TCGA-EE-A2A2-06 | TCGA-SKCM | 59.6 | Living | 0 | 0 | 0 | 0 | 0 |
| TCGA-D3-A2JF-06 | TCGA-SKCM | 62.0 | Living | 3.818 | 1 | 0 | 1 | 1 |
| TCGA-EE-A29E-06 | TCGA-SKCM | 63.7 | Living | 2.87 | 1 | 0 | 0 | 1 |
| TCGA-D3-A51R-06 | TCGA-SKCM | 63.8 | Living | 2.588 | 0 | 1 | 1 | 0 |
| TCGA-EE-A3AC-06 | TCGA-SKCM | 64.0 | Living | 2.182 | 1 | 0 | 1 | 0 |
| TCGA-EE-A3J7-06 | TCGA-SKCM | 64.0 | Living | 1.64 | 0 | 1 | 0 | 0 |
| TCGA-EE-A29H-06 | TCGA-SKCM | 64.6 | Living | 1.234 | 1 | 0 | 0 | 0 |
| TCGA-D3-A2JO-06 | TCGA-SKCM | 66.0 | Living | 2.182 | 1 | 0 | 1 | 0 |
| TCGA-EE-A2GC-06 | TCGA-SKCM | 67.4 | Living | 2.584 | 0 | 0 | 1 | 1 |
| TCGA-EB-A5SG-06 | TCGA-SKCM | 68.2 | Living | 0 | 0 | 0 | 0 | 0 |
| TCGA-EE-A2MT-06 | TCGA-SKCM | 71.2 | Living | 2.588 | 0 | 1 | 1 | 0 |
| TCGA-EE-A2GM-06 | TCGA-SKCM | 75.4 | Living | 2.182 | 1 | 0 | 1 | 0 |
| TCGA-DA-A1HV-06 | TCGA-SKCM | 76.5 | Living | 2.182 | 1 | 0 | 1 | 0 |
| TCGA-D9-A6EC-06 | TCGA-SKCM | 77.5 | Living | 5.458 | 1 | 1 | 1 | 1 |
| TCGA-ER-A19A-06 | TCGA-SKCM | 77.7 | Living | 1.234 | 1 | 0 | 0 | 0 |
| TCGA-EE-A2GL-06 | TCGA-SKCM | 79.6 | Living | 0 | 0 | 0 | 0 | 0 |
| TCGA-EE-A2A6-06 | TCGA-SKCM | 86.1 | Living | 0 | 0 | 0 | 0 | 0 |
| TCGA-D3-A2JC-06 | TCGA-SKCM | 86.7 | Living | 0 | 0 | 0 | 0 | 0 |
| TCGA-D3-A3CC-06 | TCGA-SKCM | 86.9 | Living | 0 | 0 | 0 | 0 | 0 |
| TCGA-DA-A1I7-06 | TCGA-SKCM | 88.8 | Living | 1.234 | 1 | 0 | 0 | 0 |
| TCGA-D3-A1QA-06 | TCGA-SKCM | 90.8 | Living | 0 | 0 | 0 | 0 | 0 |
| TCGA-EE-A20F-06 | TCGA-SKCM | 91.5 | Living | 0 | 0 | 0 | 0 | 0 |
| TCGA-FR-A3YN-06 | TCGA-SKCM | 92.9 | Living | 0 | 0 | 0 | 0 | 0 |
| TCGA-EE-A2GU-06 | TCGA-SKCM | 94.7 | Living | 0 | 0 | 0 | 0 | 0 |
| TCGA-D3-A1QB-06 | TCGA-SKCM | 95.7 | Living | 0 | 0 | 0 | 0 | 0 |
| TCGA-GN-A265-06 | TCGA-SKCM | 96.9 | Living | 0 | 0 | 0 | 0 | 0 |
| TCGA-FS-A1ZM-06 | TCGA-SKCM | 101.2 | Living | 0 | 0 | 0 | 0 | 0 |
| TCGA-D3-A3MR-06 | TCGA-SKCM | 103.5 | Living | 2.87 | 1 | 0 | 0 | 1 |
| TCGA-ER-A1A1-06 | TCGA-SKCM | 105.0 | Living | 0 | 0 | 0 | 0 | 0 |
| TCGA-D3-A1Q4-06 | TCGA-SKCM | 112.0 | Living | 1.234 | 1 | 0 | 0 | 0 |
| TCGA-D3-A2JA-06 | TCGA-SKCM | 115.4 | Living | 0 | 0 | 0 | 0 | 0 |
| TCGA-EE-A2A1-06 | TCGA-SKCM | 115.9 | Living | 1.234 | 1 | 0 | 0 | 0 |
| TCGA-GN-A4U3-06 | TCGA-SKCM | 121.8 | Living | 0 | 0 | 0 | 0 | 0 |
| TCGA-EE-A3AB-06 | TCGA-SKCM | 122.6 | Living | 0 | 0 | 0 | 0 | 0 |
| TCGA-EE-A3AA-06 | TCGA-SKCM | 124.2 | Living | 1.234 | 1 | 0 | 0 | 0 |
| TCGA-D3-A5GL-06 | TCGA-SKCM | 125.7 | Living | 0 | 0 | 0 | 0 | 0 |
| TCGA-EE-A2GO-06 | TCGA-SKCM | 126.7 | Living | 0 | 0 | 0 | 0 | 0 |
| TCGA-EE-A2M6-06 | TCGA-SKCM | 129.2 | Living | 0 | 0 | 0 | 0 | 0 |
| TCGA-D3-A3BZ-06 | TCGA-SKCM | 130.6 | Living | 0 | 0 | 0 | 0 | 0 |
| TCGA-D3-A1Q7-06 | TCGA-SKCM | 133.2 | Living | 0 | 0 | 0 | 0 | 0 |
| TCGA-EE-A20B-06 | TCGA-SKCM | 133.7 | Living | 1.64 | 0 | 1 | 0 | 0 |
| TCGA-EE-A3JH-06 | TCGA-SKCM | 134.2 | Living | 0 | 0 | 0 | 0 | 0 |
| TCGA-EE-A2MR-06 | TCGA-SKCM | 134.3 | Living | 4.51 | 1 | 1 | 0 | 1 |
| TCGA-DA-A1I5-06 | TCGA-SKCM | 134.9 | Living | 1.234 | 1 | 0 | 0 | 0 |
| TCGA-D3-A5GN-06 | TCGA-SKCM | 135.6 | Living | 0 | 0 | 0 | 0 | 0 |
| TCGA-D3-A5GO-06 | TCGA-SKCM | 137.8 | Living | 0 | 0 | 0 | 0 | 0 |
| TCGA-GN-A262-06 | TCGA-SKCM | 139.8 | Living | 0 | 0 | 0 | 0 | 0 |
| TCGA-DA-A1HY-06 | TCGA-SKCM | 144.8 | Living | 0 | 0 | 0 | 0 | 0 |
| TCGA-D3-A51J-06 | TCGA-SKCM | 145.0 | Living | 0 | 0 | 0 | 0 | 0 |
| TCGA-FS-A1ZS-06 | TCGA-SKCM | 148.7 | Living | 0 | 0 | 0 | 0 | 0 |
| TCGA-ER-A42L-06 | TCGA-SKCM | 148.9 | Living | 2.87 | 1 | 0 | 0 | 1 |
| TCGA-D9-A148-06 | TCGA-SKCM | 151.4 | Living | 0 | 0 | 0 | 0 | 0 |
| TCGA-EE-A2MS-06 | TCGA-SKCM | 162.4 | Living | 3.818 | 1 | 0 | 1 | 1 |
| TCGA-D3-A3CB-06 | TCGA-SKCM | 166.4 | Living | 1.234 | 1 | 0 | 0 | 0 |
| TCGA-D3-A2JL-06 | TCGA-SKCM | 171.5 | Living | 0 | 0 | 0 | 0 | 0 |
| TCGA-EE-A2GE-06 | TCGA-SKCM | 173.7 | Living | 0 | 0 | 0 | 0 | 0 |
| TCGA-FR-A44A-06 | TCGA-SKCM | 174.1 | Living | 1.234 | 1 | 0 | 0 | 0 |
| TCGA-D3-A51E-06 | TCGA-SKCM | 174.7 | Living | 1.234 | 1 | 0 | 0 | 0 |
| TCGA-D3-A5GR-06 | TCGA-SKCM | 178.2 | Living | 0 | 0 | 0 | 0 | 0 |
| TCGA-EE-A2MK-06 | TCGA-SKCM | 180.3 | Living | 1.636 | 0 | 0 | 0 | 1 |
| TCGA-EE-A29W-06 | TCGA-SKCM | 194.9 | Living | 0 | 0 | 0 | 0 | 0 |
| TCGA-EE-A3JB-06 | TCGA-SKCM | 201.6 | Living | 2.182 | 1 | 0 | 1 | 0 |
| TCGA-EE-A2GH-06 | TCGA-SKCM | 220.1 | Living | 0 | 0 | 0 | 0 | 0 |
| TCGA-DA-A1I1-06 | TCGA-SKCM | 222.3 | Living | 0 | 0 | 0 | 0 | 0 |
| TCGA-EE-A2MP-06 | TCGA-SKCM | 248.5 | Living | 1.234 | 1 | 0 | 0 | 0 |
| TCGA-ER-A19G-06 | TCGA-SKCM | 301.8 | Living | 0 | 0 | 0 | 0 | 0 |
| TCGA-EE-A29T-06 | TCGA-SKCM | 369.7 | Living | 1.234 | 1 | 0 | 0 | 0 |
| TCGA-ER-A19O-06 | TCGA-SKCM | NA | Deceased | 0 | 0 | 0 | 0 | 0 |
| TCGA-EE-A29L-06 | TCGA-SKCM | 2.6 | Deceased | 2.87 | 1 | 0 | 0 | 1 |
| TCGA-EE-A185-06 | TCGA-SKCM | 5.0 | Deceased | 1.234 | 1 | 0 | 0 | 0 |
| TCGA-GN-A269-01 | TCGA-SKCM | 5.6 | Deceased | 0 | 0 | 0 | 0 | 0 |
| TCGA-D9-A4Z2-01 | TCGA-SKCM | 6.2 | Deceased | 0 | 0 | 0 | 0 | 0 |
| TCGA-ER-A19J-06 | TCGA-SKCM | 6.4 | Deceased | 0 | 0 | 0 | 0 | 0 |
| TCGA-EB-A44N-01 | TCGA-SKCM | 6.7 | Deceased | 1.234 | 1 | 0 | 0 | 0 |
| TCGA-D9-A1JX-06 | TCGA-SKCM | 7.1 | Deceased | 0 | 0 | 0 | 0 | 0 |
| TCGA-FS-A1Z7-06 | TCGA-SKCM | 7.8 | Deceased | 1.234 | 1 | 0 | 0 | 0 |
| TCGA-EE-A17Z-06 | TCGA-SKCM | 8.6 | Deceased | 0 | 0 | 0 | 0 | 0 |
| TCGA-ER-A19T-01 | TCGA-SKCM | 8.9 | Deceased | 0 | 0 | 0 | 0 | 0 |
| TCGA-ER-A199-06 | TCGA-SKCM | 9.2 | Deceased | 1.636 | 0 | 0 | 0 | 1 |
| TCGA-BF-A1PX-01 | TCGA-SKCM | 9.3 | Deceased | 0.948 | 0 | 0 | 1 | 0 |
| TCGA-D3-A3MO-06 | TCGA-SKCM | 9.3 | Deceased | 0 | 0 | 0 | 0 | 0 |
| TCGA-FS-A1ZG-06 | TCGA-SKCM | 9.7 | Deceased | 0 | 0 | 0 | 0 | 0 |
| TCGA-FR-A726-01 | TCGA-SKCM | 10.0 | Deceased | 3.818 | 1 | 0 | 1 | 1 |
| TCGA-GN-A266-06 | TCGA-SKCM | 10.1 | Deceased | 5.458 | 1 | 1 | 1 | 1 |
| TCGA-EB-A44R-06 | TCGA-SKCM | 10.4 | Deceased | 0 | 0 | 0 | 0 | 0 |
| TCGA-GN-A4U7-06 | TCGA-SKCM | 10.4 | Deceased | 1.234 | 1 | 0 | 0 | 0 |
| TCGA-DA-A3F3-06 | TCGA-SKCM | 10.5 | Deceased | 0 | 0 | 0 | 0 | 0 |
| TCGA-EB-A5VU-01 | TCGA-SKCM | 10.6 | Deceased | 0 | 0 | 0 | 0 | 0 |
| TCGA-EB-A4P0-01 | TCGA-SKCM | 10.7 | Deceased | 0 | 0 | 0 | 0 | 0 |
| TCGA-EB-A3Y7-01 | TCGA-SKCM | 10.7 | Deceased | 3.822 | 1 | 1 | 1 | 0 |
| TCGA-FS-A1ZR-06 | TCGA-SKCM | 11.4 | Deceased | 0 | 0 | 0 | 0 | 0 |
| TCGA-EB-A6QZ-01 | TCGA-SKCM | 11.6 | Deceased | 1.636 | 0 | 0 | 0 | 1 |
| TCGA-D3-A2JD-06 | TCGA-SKCM | 11.9 | Deceased | 0.948 | 0 | 0 | 1 | 0 |
| TCGA-D3-A2JK-06 | TCGA-SKCM | 12.1 | Deceased | 0 | 0 | 0 | 0 | 0 |
| TCGA-FR-A2OS-01 | TCGA-SKCM | 12.1 | Deceased | 0 | 0 | 0 | 0 | 0 |
| TCGA-EB-A5SF-01 | TCGA-SKCM | 12.1 | Deceased | 0 | 0 | 0 | 0 | 0 |
| TCGA-ER-A19D-06 | TCGA-SKCM | 12.6 | Deceased | 0 | 0 | 0 | 0 | 0 |
| TCGA-ER-A42K-06 | TCGA-SKCM | 12.9 | Deceased | 0 | 0 | 0 | 0 | 0 |
| TCGA-ER-A19E-06 | TCGA-SKCM | 13.0 | Deceased | 1.636 | 0 | 0 | 0 | 1 |
| TCGA-EB-A5SE-01 | TCGA-SKCM | 13.2 | Deceased | 0 | 0 | 0 | 0 | 0 |
| TCGA-EE-A20I-06 | TCGA-SKCM | 13.5 | Deceased | 0 | 0 | 0 | 0 | 0 |
| TCGA-EE-A3AF-06 | TCGA-SKCM | 13.8 | Deceased | 3.276 | 0 | 1 | 0 | 1 |
| TCGA-D3-A3ML-06 | TCGA-SKCM | 13.9 | Deceased | 0 | 0 | 0 | 0 | 0 |
| TCGA-EE-A2GP-06 | TCGA-SKCM | 13.9 | Deceased | 2.87 | 1 | 0 | 0 | 1 |
| TCGA-ER-A197-06 | TCGA-SKCM | 13.9 | Deceased | 0 | 0 | 0 | 0 | 0 |
| TCGA-EE-A29D-06 | TCGA-SKCM | 14.0 | Deceased | 5.458 | 1 | 1 | 1 | 1 |
| TCGA-ER-A42H-01 | TCGA-SKCM | 14.0 | Deceased | 0 | 0 | 0 | 0 | 0 |
| TCGA-EE-A182-06 | TCGA-SKCM | 14.7 | Deceased | 1.234 | 1 | 0 | 0 | 0 |
| TCGA-EB-A5FP-01 | TCGA-SKCM | 14.9 | Deceased | 0 | 0 | 0 | 0 | 0 |
| TCGA-GN-A263-01 | TCGA-SKCM | 15.3 | Deceased | 0 | 0 | 0 | 0 | 0 |
| TCGA-D9-A3Z1-06 | TCGA-SKCM | 15.4 | Deceased | 0 | 0 | 0 | 0 | 0 |
| TCGA-ER-A19K-01 | TCGA-SKCM | 15.4 | Deceased | 0 | 0 | 0 | 0 | 0 |
| TCGA-FS-A1ZF-06 | TCGA-SKCM | 15.4 | Deceased | 0 | 0 | 0 | 0 | 0 |
| TCGA-EB-A57M-01 | TCGA-SKCM | 15.5 | Deceased | 0 | 0 | 0 | 0 | 0 |
| TCGA-D3-A1Q1-06 | TCGA-SKCM | 16.6 | Deceased | 0 | 0 | 0 | 0 | 0 |
| TCGA-D3-A1Q3-06 | TCGA-SKCM | 16.7 | Deceased | 0 | 0 | 0 | 0 | 0 |
| TCGA-EE-A2MH-06 | TCGA-SKCM | 17.0 | Deceased | 0 | 0 | 0 | 0 | 0 |
| TCGA-D9-A3Z4-01 | TCGA-SKCM | 17.1 | Deceased | 0 | 0 | 0 | 0 | 0 |
| TCGA-EE-A29X-06 | TCGA-SKCM | 17.9 | Deceased | 0 | 0 | 0 | 0 | 0 |
| TCGA-EE-A29N-06 | TCGA-SKCM | 18.6 | Deceased | 2.87 | 1 | 0 | 0 | 1 |
| TCGA-EE-A2M8-06 | TCGA-SKCM | 19.7 | Deceased | 0 | 0 | 0 | 0 | 0 |
| TCGA-EB-A6R0-01 | TCGA-SKCM | 20.0 | Deceased | 1.234 | 1 | 0 | 0 | 0 |
| TCGA-ER-A2NE-06 | TCGA-SKCM | 20.1 | Deceased | 1.636 | 0 | 0 | 0 | 1 |
| TCGA-EB-A5KH-06 | TCGA-SKCM | 20.3 | Deceased | 0 | 0 | 0 | 0 | 0 |
| TCGA-DA-A1I0-06 | TCGA-SKCM | 20.4 | Deceased | 1.636 | 0 | 0 | 0 | 1 |
| TCGA-EB-A4IQ-01 | TCGA-SKCM | 20.9 | Deceased | 0 | 0 | 0 | 0 | 0 |
| TCGA-FS-A1Z3-06 | TCGA-SKCM | 20.9 | Deceased | 0 | 0 | 0 | 0 | 0 |
| TCGA-EE-A2M5-06 | TCGA-SKCM | 21.7 | Deceased | 5.458 | 1 | 1 | 1 | 1 |
| TCGA-GN-A4U9-06 | TCGA-SKCM | 22.1 | Deceased | 0 | 0 | 0 | 0 | 0 |
| TCGA-D9-A6EG-06 | TCGA-SKCM | 22.9 | Deceased | 0 | 0 | 0 | 0 | 0 |
| TCGA-ER-A2ND-06 | TCGA-SKCM | 23.3 | Deceased | 0 | 0 | 0 | 0 | 0 |
| TCGA-EB-A42Y-01 | TCGA-SKCM | 23.7 | Deceased | 0 | 0 | 0 | 0 | 0 |
| TCGA-D3-A2J9-06 | TCGA-SKCM | 23.8 | Deceased | 0 | 0 | 0 | 0 | 0 |
| TCGA-FS-A1ZK-06 | TCGA-SKCM | 23.9 | Deceased | 0 | 0 | 0 | 0 | 0 |
| TCGA-FS-A1ZN-01 | TCGA-SKCM | 24.0 | Deceased | 0 | 0 | 0 | 0 | 0 |
| TCGA-D3-A3CF-06 | TCGA-SKCM | 24.5 | Deceased | 0 | 0 | 0 | 0 | 0 |
| TCGA-EE-A29V-06 | TCGA-SKCM | 25.9 | Deceased | 2.87 | 1 | 0 | 0 | 1 |
| TCGA-ER-A19F-06 | TCGA-SKCM | 26.4 | Deceased | 0.948 | 0 | 0 | 1 | 0 |
| TCGA-FS-A1ZU-06 | TCGA-SKCM | 26.5 | Deceased | 0 | 0 | 0 | 0 | 0 |
| TCGA-FS-A4FB-06 | TCGA-SKCM | 26.7 | Deceased | 0 | 0 | 0 | 0 | 0 |
| TCGA-EE-A183-06 | TCGA-SKCM | 26.9 | Deceased | 1.64 | 0 | 1 | 0 | 0 |
| TCGA-GN-A26C-01 | TCGA-SKCM | 27.0 | Deceased | 2.182 | 1 | 0 | 1 | 0 |
| TCGA-FS-A1ZZ-06 | TCGA-SKCM | 27.0 | Deceased | 4.224 | 0 | 1 | 1 | 1 |
| TCGA-FS-A1ZY-06 | TCGA-SKCM | 27.1 | Deceased | 0 | 0 | 0 | 0 | 0 |
| TCGA-EE-A17Y-06 | TCGA-SKCM | 27.2 | Deceased | 0 | 0 | 0 | 0 | 0 |
| TCGA-EE-A3JD-06 | TCGA-SKCM | 27.3 | Deceased | 5.458 | 1 | 1 | 1 | 1 |
| TCGA-FS-A1ZA-06 | TCGA-SKCM | 27.7 | Deceased | 0 | 0 | 0 | 0 | 0 |
| TCGA-FS-A1Z4-06 | TCGA-SKCM | 28.1 | Deceased | 0 | 0 | 0 | 0 | 0 |
| TCGA-D3-A1Q8-06 | TCGA-SKCM | 28.1 | Deceased | 0 | 0 | 0 | 0 | 0 |
| TCGA-ER-A2NB-01 | TCGA-SKCM | 28.2 | Deceased | 0 | 0 | 0 | 0 | 0 |
| TCGA-FS-A4F5-06 | TCGA-SKCM | 28.7 | Deceased | 1.234 | 1 | 0 | 0 | 0 |
| TCGA-EE-A3AD-06 | TCGA-SKCM | 28.8 | Deceased | 0 | 0 | 0 | 0 | 0 |
| TCGA-ER-A2NF-01 | TCGA-SKCM | 28.8 | Deceased | 0 | 0 | 0 | 0 | 0 |
| TCGA-EE-A2M7-06 | TCGA-SKCM | 28.8 | Deceased | 0 | 0 | 0 | 0 | 0 |
| TCGA-EE-A17X-06 | TCGA-SKCM | 29.8 | Deceased | 3.276 | 0 | 1 | 0 | 1 |
| TCGA-ER-A193-06 | TCGA-SKCM | 31.4 | Deceased | 4.51 | 1 | 1 | 0 | 1 |
| TCGA-D3-A1Q9-06 | TCGA-SKCM | 31.6 | Deceased | 0 | 0 | 0 | 0 | 0 |
| TCGA-GN-A26A-06 | TCGA-SKCM | 32.5 | Deceased | 0 | 0 | 0 | 0 | 0 |
| TCGA-FS-A1ZH-06 | TCGA-SKCM | 32.7 | Deceased | 0 | 0 | 0 | 0 | 0 |
| TCGA-EE-A181-06 | TCGA-SKCM | 33.7 | Deceased | 3.818 | 1 | 0 | 1 | 1 |
| TCGA-DA-A3F2-06 | TCGA-SKCM | 33.9 | Deceased | 0 | 0 | 0 | 0 | 0 |
| TCGA-EE-A3J8-06 | TCGA-SKCM | 34.3 | Deceased | 0.948 | 0 | 0 | 1 | 0 |
| TCGA-GF-A769-01 | TCGA-SKCM | 35.2 | Deceased | 0 | 0 | 0 | 0 | 0 |
| TCGA-ER-A195-06 | TCGA-SKCM | 35.4 | Deceased | 0 | 0 | 0 | 0 | 0 |
| TCGA-DA-A1I4-06 | TCGA-SKCM | 35.9 | Deceased | 0 | 0 | 0 | 0 | 0 |
| TCGA-DA-A1HW-06 | TCGA-SKCM | 36.0 | Deceased | 0.948 | 0 | 0 | 1 | 0 |
| TCGA-EE-A3J5-06 | TCGA-SKCM | 36.9 | Deceased | 5.458 | 1 | 1 | 1 | 1 |
| TCGA-EE-A2A5-06 | TCGA-SKCM | 39.3 | Deceased | 0 | 0 | 0 | 0 | 0 |
| TCGA-DA-A1IB-06 | TCGA-SKCM | 40.6 | Deceased | 0 | 0 | 0 | 0 | 0 |
| TCGA-EE-A3AG-06 | TCGA-SKCM | 41.6 | Deceased | 1.234 | 1 | 0 | 0 | 0 |
| TCGA-EE-A2GR-06 | TCGA-SKCM | 42.7 | Deceased | 5.458 | 1 | 1 | 1 | 1 |
| TCGA-EE-A2MQ-06 | TCGA-SKCM | 43.2 | Deceased | 0 | 0 | 0 | 0 | 0 |
| TCGA-D3-A2J6-06 | TCGA-SKCM | 43.4 | Deceased | 0 | 0 | 0 | 0 | 0 |
| TCGA-ER-A2NC-06 | TCGA-SKCM | 43.8 | Deceased | 1.234 | 1 | 0 | 0 | 0 |
| TCGA-ER-A19N-06 | TCGA-SKCM | 44.1 | Deceased | 0 | 0 | 0 | 0 | 0 |
| TCGA-ER-A194-01 | TCGA-SKCM | 44.5 | Deceased | 3.276 | 0 | 1 | 0 | 1 |
| TCGA-FS-A1ZE-06 | TCGA-SKCM | 46.4 | Deceased | 0 | 0 | 0 | 0 | 0 |
| TCGA-EE-A2A0-06 | TCGA-SKCM | 46.8 | Deceased | 0 | 0 | 0 | 0 | 0 |
| TCGA-ER-A3EV-06 | TCGA-SKCM | 46.9 | Deceased | 0 | 0 | 0 | 0 | 0 |
| TCGA-EE-A2MD-06 | TCGA-SKCM | 47.2 | Deceased | 3.818 | 1 | 0 | 1 | 1 |
| TCGA-FS-A1ZJ-06 | TCGA-SKCM | 47.3 | Deceased | 0 | 0 | 0 | 0 | 0 |
| TCGA-EE-A2MN-06 | TCGA-SKCM | 47.5 | Deceased | 1.234 | 1 | 0 | 0 | 0 |
| TCGA-GN-A26D-06 | TCGA-SKCM | 48.0 | Deceased | 0 | 0 | 0 | 0 | 0 |
| TCGA-FS-A1YX-06 | TCGA-SKCM | 48.6 | Deceased | 0.948 | 0 | 0 | 1 | 0 |
| TCGA-FS-A1ZB-06 | TCGA-SKCM | 48.8 | Deceased | 0 | 0 | 0 | 0 | 0 |
| TCGA-ER-A19C-06 | TCGA-SKCM | 48.9 | Deceased | 0 | 0 | 0 | 0 | 0 |
| TCGA-ER-A2NG-06 | TCGA-SKCM | 49.0 | Deceased | 0 | 0 | 0 | 0 | 0 |
| TCGA-ER-A198-06 | TCGA-SKCM | 50.7 | Deceased | 2.87 | 1 | 0 | 0 | 1 |
| TCGA-ER-A19Q-06 | TCGA-SKCM | 50.9 | Deceased | 0 | 0 | 0 | 0 | 0 |
| TCGA-EE-A3JA-06 | TCGA-SKCM | 53.2 | Deceased | 2.874 | 1 | 1 | 0 | 0 |
| TCGA-FS-A1ZD-06 | TCGA-SKCM | 53.5 | Deceased | 0 | 0 | 0 | 0 | 0 |
| TCGA-DA-A1I8-06 | TCGA-SKCM | 53.9 | Deceased | 0 | 0 | 0 | 0 | 0 |
| TCGA-FS-A4FC-06 | TCGA-SKCM | 54.4 | Deceased | 2.182 | 1 | 0 | 1 | 0 |
| TCGA-FW-A3TU-06 | TCGA-SKCM | 55.6 | Deceased | 0 | 0 | 0 | 0 | 0 |
| TCGA-D3-A3C6-06 | TCGA-SKCM | 58.0 | Deceased | 0 | 0 | 0 | 0 | 0 |
| TCGA-D3-A3CE-06 | TCGA-SKCM | 60.2 | Deceased | 0 | 0 | 0 | 0 | 0 |
| TCGA-ER-A19M-06 | TCGA-SKCM | 61.0 | Deceased | 2.87 | 1 | 0 | 0 | 1 |
| TCGA-EE-A29S-06 | TCGA-SKCM | 61.2 | Deceased | 2.182 | 1 | 0 | 1 | 0 |
| TCGA-EE-A2MC-06 | TCGA-SKCM | 61.5 | Deceased | 2.182 | 1 | 0 | 1 | 0 |
| TCGA-GN-A268-06 | TCGA-SKCM | 62.8 | Deceased | 1.234 | 1 | 0 | 0 | 0 |
| TCGA-EE-A29A-06 | TCGA-SKCM | 63.3 | Deceased | 1.234 | 1 | 0 | 0 | 0 |
| TCGA-GN-A267-06 | TCGA-SKCM | 64.4 | Deceased | 0 | 0 | 0 | 0 | 0 |
| TCGA-D3-A2J8-06 | TCGA-SKCM | 65.4 | Deceased | 2.874 | 1 | 1 | 0 | 0 |
| TCGA-DA-A1IA-06 | TCGA-SKCM | 65.9 | Deceased | 1.234 | 1 | 0 | 0 | 0 |
| TCGA-D3-A2JN-06 | TCGA-SKCM | 66.4 | Deceased | 1.636 | 0 | 0 | 0 | 1 |
| TCGA-FS-A4F4-06 | TCGA-SKCM | 66.6 | Deceased | 0 | 0 | 0 | 0 | 0 |
| TCGA-EE-A29Q-06 | TCGA-SKCM | 66.7 | Deceased | 0 | 0 | 0 | 0 | 0 |
| TCGA-DA-A1IC-06 | TCGA-SKCM | 68.0 | Deceased | 0 | 0 | 0 | 0 | 0 |
| TCGA-EE-A184-06 | TCGA-SKCM | 68.1 | Deceased | 0 | 0 | 0 | 0 | 0 |
| TCGA-D3-A1Q6-06 | TCGA-SKCM | 71.8 | Deceased | 3.818 | 1 | 0 | 1 | 1 |
| TCGA-EE-A29G-06 | TCGA-SKCM | 72.0 | Deceased | 1.234 | 1 | 0 | 0 | 0 |
| TCGA-FS-A1ZP-06 | TCGA-SKCM | 74.7 | Deceased | 3.822 | 1 | 1 | 1 | 0 |
| TCGA-EE-A29C-06 | TCGA-SKCM | 78.9 | Deceased | 0.948 | 0 | 0 | 1 | 0 |
| TCGA-FS-A4FD-06 | TCGA-SKCM | 80.6 | Deceased | 0 | 0 | 0 | 0 | 0 |
| TCGA-EE-A2GS-06 | TCGA-SKCM | 81.1 | Deceased | 0 | 0 | 0 | 0 | 0 |
| TCGA-EE-A29B-06 | TCGA-SKCM | 85.0 | Deceased | 0 | 0 | 0 | 0 | 0 |
| TCGA-ER-A3ET-06 | TCGA-SKCM | 92.9 | Deceased | 0 | 0 | 0 | 0 | 0 |
| TCGA-EE-A180-06 | TCGA-SKCM | 94.9 | Deceased | 0 | 0 | 0 | 0 | 0 |
| TCGA-EE-A2MJ-06 | TCGA-SKCM | 96.2 | Deceased | 2.87 | 1 | 0 | 0 | 1 |
| TCGA-ER-A19B-06 | TCGA-SKCM | 98.3 | Deceased | 0 | 0 | 0 | 0 | 0 |
| TCGA-EE-A2GN-06 | TCGA-SKCM | 102.0 | Deceased | 0 | 0 | 0 | 0 | 0 |
| TCGA-D3-A2J7-06 | TCGA-SKCM | 103.0 | Deceased | 1.636 | 0 | 0 | 0 | 1 |
| TCGA-EE-A2MG-06 | TCGA-SKCM | 103.1 | Deceased | 0 | 0 | 0 | 0 | 0 |
| TCGA-EE-A2ME-06 | TCGA-SKCM | 103.2 | Deceased | 0 | 0 | 0 | 0 | 0 |
| TCGA-EE-A2GJ-06 | TCGA-SKCM | 107.3 | Deceased | 0 | 0 | 0 | 0 | 0 |
| TCGA-D3-A1Q5-06 | TCGA-SKCM | 112.5 | Deceased | 2.584 | 0 | 0 | 1 | 1 |
| TCGA-D3-A2JG-06 | TCGA-SKCM | 113.4 | Deceased | 0 | 0 | 0 | 0 | 0 |
| TCGA-GN-A264-06 | TCGA-SKCM | 117.8 | Deceased | 0 | 0 | 0 | 0 | 0 |
| TCGA-EE-A3J4-06 | TCGA-SKCM | 127.1 | Deceased | 0.948 | 0 | 0 | 1 | 0 |
| TCGA-ER-A19L-06 | TCGA-SKCM | 131.4 | Deceased | 0 | 0 | 0 | 0 | 0 |
| TCGA-FS-A1ZQ-06 | TCGA-SKCM | 133.4 | Deceased | 0 | 0 | 0 | 0 | 0 |
| TCGA-EE-A3AH-06 | TCGA-SKCM | 138.7 | Deceased | 0 | 0 | 0 | 0 | 0 |
| TCGA-ER-A19W-06 | TCGA-SKCM | 148.1 | Deceased | 0 | 0 | 0 | 0 | 0 |
| TCGA-EE-A20C-06 | TCGA-SKCM | 151.2 | Deceased | 2.874 | 1 | 1 | 0 | 0 |
| TCGA-ER-A19H-06 | TCGA-SKCM | 152.2 | Deceased | 0 | 0 | 0 | 0 | 0 |
| TCGA-EE-A3JI-06 | TCGA-SKCM | 152.7 | Deceased | 1.234 | 1 | 0 | 0 | 0 |
| TCGA-ER-A19P-06 | TCGA-SKCM | 162.0 | Deceased | 1.64 | 0 | 1 | 0 | 0 |
| TCGA-EE-A2MM-06 | TCGA-SKCM | 167.8 | Deceased | 0 | 0 | 0 | 0 | 0 |
| TCGA-D3-A2JB-06 | TCGA-SKCM | 167.9 | Deceased | 0 | 0 | 0 | 0 | 0 |
| TCGA-EE-A20H-06 | TCGA-SKCM | 168.1 | Deceased | 2.87 | 1 | 0 | 0 | 1 |
| TCGA-EE-A3J3-06 | TCGA-SKCM | 172.0 | Deceased | 1.234 | 1 | 0 | 0 | 0 |
| TCGA-FS-A4F8-06 | TCGA-SKCM | 174.7 | Deceased | 0 | 0 | 0 | 0 | 0 |
| TCGA-DA-A1I2-06 | TCGA-SKCM | 176.4 | Deceased | 0 | 0 | 0 | 0 | 0 |
| TCGA-FS-A1Z0-06 | TCGA-SKCM | 202.5 | Deceased | 1.234 | 1 | 0 | 0 | 0 |
| TCGA-EE-A2MI-06 | TCGA-SKCM | 204.5 | Deceased | 1.234 | 1 | 0 | 0 | 0 |
| TCGA-EE-A2ML-06 | TCGA-SKCM | 216.5 | Deceased | 1.234 | 1 | 0 | 0 | 0 |
| TCGA-FS-A1YW-06 | TCGA-SKCM | 216.8 | Deceased | 0 | 0 | 0 | 0 | 0 |
| TCGA-DA-A3F5-06 | TCGA-SKCM | 225.8 | Deceased | 0 | 0 | 0 | 0 | 0 |
| TCGA-FS-A1YY-06 | TCGA-SKCM | 228.4 | Deceased | 0 | 0 | 0 | 0 | 0 |
| TCGA-ER-A3ES-06 | TCGA-SKCM | 246.9 | Deceased | 0 | 0 | 0 | 0 | 0 |
| TCGA-EE-A2MF-06 | TCGA-SKCM | 268.5 | Deceased | 0 | 0 | 0 | 0 | 0 |
| TCGA-OD-A75X-06 | TCGA-SKCM | 297.7 | Deceased | 0.948 | 0 | 0 | 1 | 0 |
| TCGA-EE-A2GD-06 | TCGA-SKCM | 339.9 | Deceased | 1.234 | 1 | 0 | 0 | 0 |
| TCGA-FS-A1ZC-06 | TCGA-SKCM | 357.1 | Deceased | 0 | 0 | 0 | 0 | 0 |
| DO220846 | ICGC-MELA | 9.8 | Deceased | 0 | 0 | 0 | 0 | 0 |
| DO220845 | ICGC-MELA | 90.6 | Deceased | 0 | 0 | 0 | 0 | 0 |
| DO220848 | ICGC-MELA | 63.9 | Living | 0 | 0 | 0 | 0 | 0 |
| DO220847 | ICGC-MELA | 57.7 | Deceased | 0 | 0 | 0 | 0 | 0 |
| DO220841 | ICGC-MELA | 202.7 | Living | 0 | 0 | 0 | 0 | 0 |
| DO220844 | ICGC-MELA | 162.3 | Deceased | 0 | 0 | 0 | 0 | 0 |
| DO220843 | ICGC-MELA | 17.3 | Deceased | 0 | 0 | 0 | 0 | 0 |
| DO220849 | ICGC-MELA | 10.8 | Deceased | 0 | 0 | 0 | 0 | 0 |
| DO220850 | ICGC-MELA | 140.6 | Living | 0 | 0 | 0 | 0 | 0 |
| DO220859 | ICGC-MELA | 158.4 | Deceased | 0 | 0 | 0 | 0 | 0 |
| DO220858 | ICGC-MELA | 19.7 | Deceased | 0 | 0 | 0 | 0 | 0 |
| DO220854 | ICGC-MELA | 281.5 | Living | 0 | 0 | 0 | 0 | 0 |
| DO220860 | ICGC-MELA | 183.4 | Living | 0 | 0 | 0 | 0 | 0 |
| DO220868 | ICGC-MELA | 41.9 | Living | 0 | 0 | 0 | 0 | 0 |
| DO220867 | ICGC-MELA | 42.5 | Living | 0 | 0 | 0 | 0 | 0 |
| DO220869 | ICGC-MELA | 123.0 | Living | 0 | 0 | 0 | 0 | 0 |
| DO220864 | ICGC-MELA | 62.0 | Living | 0 | 0 | 0 | 0 | 0 |
| DO220863 | ICGC-MELA | 50.1 | Deceased | 0 | 0 | 0 | 0 | 0 |
| DO220866 | ICGC-MELA | 39.6 | Deceased | 0 | 0 | 0 | 0 | 0 |
| DO220865 | ICGC-MELA | 17.4 | Living | 0 | 0 | 0 | 0 | 0 |
| DO220871 | ICGC-MELA | 4.3 | Living | 0 | 0 | 0 | 0 | 0 |
| DO220870 | ICGC-MELA | 64.1 | Deceased | 0 | 0 | 0 | 0 | 0 |
| DO220879 | ICGC-MELA | 60.3 | Deceased | 0 | 0 | 0 | 0 | 0 |
| DO220881 | ICGC-MELA | 186.3 | Living | 0 | 0 | 0 | 0 | 0 |
| DO220888 | ICGC-MELA | 48.2 | Deceased | 0 | 0 | 0 | 0 | 0 |
| DO220887 | ICGC-MELA | 83.2 | Living | 0 | 0 | 0 | 0 | 0 |
| DO220892 | ICGC-MELA | 177.5 | Deceased | 0 | 0 | 0 | 0 | 0 |
| DO220895 | ICGC-MELA | 112.6 | Living | 0 | 0 | 0 | 0 | 0 |
| DO220894 | ICGC-MELA | 0.0 | Living | 0 | 0 | 0 | 0 | 0 |
| DO220897 | ICGC-MELA | 74.5 | Living | 0 | 0 | 0 | 0 | 0 |
| DO220896 | ICGC-MELA | 124.5 | Living | 0 | 0 | 0 | 0 | 0 |
| DO219882 | ICGC-MELA | 14.7 | Deceased | 0 | 0 | 0 | 0 | 0 |
| DO219880 | ICGC-MELA | 8.2 | Deceased | 0 | 0 | 0 | 0 | 0 |
| DO219883 | ICGC-MELA | 16.2 | Deceased | 0 | 0 | 0 | 0 | 0 |
| DO219877 | ICGC-MELA | 53.6 | Living | 0 | 0 | 0 | 0 | 0 |
| DO219876 | ICGC-MELA | 10.1 | Deceased | 0 | 0 | 0 | 0 | 0 |
| DO219875 | ICGC-MELA | 3.5 | Deceased | 0 | 0 | 0 | 0 | 0 |
| DO219872 | ICGC-MELA | 53.2 | Living | 0 | 0 | 0 | 0 | 0 |
| DO228017 | ICGC-MELA | 33.8 | Deceased | 0 | 0 | 0 | 0 | 0 |
| DO228016 | ICGC-MELA | 11.7 | Deceased | 0 | 0 | 0 | 0 | 0 |
| DO228019 | ICGC-MELA | 97.6 | Deceased | 0 | 0 | 0 | 0 | 0 |
| DO228018 | ICGC-MELA | 37.0 | Deceased | 0 | 0 | 0 | 0 | 0 |
| DO228010 | ICGC-MELA | 24.1 | Deceased | 0 | 0 | 0 | 0 | 0 |
| DO228013 | ICGC-MELA | 59.4 | Living | 0 | 0 | 0 | 0 | 0 |
| DO228012 | ICGC-MELA | 80.9 | Living | 0 | 0 | 0 | 0 | 0 |
| DO228015 | ICGC-MELA | 59.9 | Deceased | 0 | 0 | 0 | 0 | 0 |
| DO228020 | ICGC-MELA | 66.2 | Living | 0 | 0 | 0 | 0 | 0 |
| DO220909 | ICGC-MELA | 45.5 | Living | 0 | 0 | 0 | 0 | 0 |
| DO220908 | ICGC-MELA | 114.6 | Living | 0 | 0 | 0 | 0 | 0 |
| DO220904 | ICGC-MELA | 100.8 | Deceased | 0 | 0 | 0 | 0 | 0 |
| DO229499 | ICGC-MELA | 74.0 | Deceased | 0 | 0 | 0 | 0 | 0 |
| DO229467 | ICGC-MELA | 165.6 | Deceased | 0 | 0 | 0 | 0 | 0 |
| DO229479 | ICGC-MELA | 102.9 | Living | 0 | 0 | 0 | 0 | 0 |
| DO229475 | ICGC-MELA | 4.1 | Deceased | 0 | 0 | 0 | 0 | 0 |
| DO229485 | ICGC-MELA | 32.0 | Living | 0 | 0 | 0 | 0 | 0 |
| DO229491 | ICGC-MELA | 57.0 | Deceased | 0 | 0 | 0 | 0 | 0 |
| DO218910 | ICGC-MELA | 10.1 | Deceased | 0 | 0 | 0 | 0 | 0 |
| DO218914 | ICGC-MELA | 126.4 | Living | 0 | 0 | 0 | 0 | 0 |
| DO218908 | ICGC-MELA | 79.4 | Living | 0 | 0 | 0 | 0 | 0 |
| DO218906 | ICGC-MELA | 11.8 | Deceased | 0 | 0 | 0 | 0 | 0 |
| DO218904 | ICGC-MELA | 48.0 | Deceased | 0 | 0 | 0 | 0 | 0 |
| DO218902 | ICGC-MELA | 51.0 | Living | 0 | 0 | 0 | 0 | 0 |
| DO218876 | ICGC-MELA | 41.4 | Deceased | 0 | 0 | 0 | 0 | 0 |
| DO218870 | ICGC-MELA | 22.0 | Deceased | 0 | 0 | 0 | 0 | 0 |
| DO218872 | ICGC-MELA | 158.1 | Living | 0 | 0 | 0 | 0 | 0 |
| DO218866 | ICGC-MELA | 62.3 | Deceased | 0 | 0 | 0 | 0 | 0 |
| DO218864 | ICGC-MELA | 49.5 | Deceased | 0 | 0 | 0 | 0 | 0 |
| DO218868 | ICGC-MELA | 19.7 | Deceased | 0 | 0 | 0 | 0 | 0 |
| DO218862 | ICGC-MELA | 8.7 | Deceased | 0 | 0 | 0 | 0 | 0 |
| DO218860 | ICGC-MELA | 34.6 | Deceased | 0 | 0 | 0 | 0 | 0 |
| DO218856 | ICGC-MELA | 167.8 | Deceased | 0 | 0 | 0 | 0 | 0 |
| DO218855 | ICGC-MELA | 24.7 | Deceased | 0 | 0 | 0 | 0 | 0 |
| DO218854 | ICGC-MELA | 29.7 | Living | 0 | 0 | 0 | 0 | 0 |
| DO218853 | ICGC-MELA | 35.4 | Deceased | 0 | 0 | 0 | 0 | 0 |
| DO218859 | ICGC-MELA | 75.4 | Deceased | 0 | 0 | 0 | 0 | 0 |
| DO218858 | ICGC-MELA | 73.1 | Deceased | 0 | 0 | 0 | 0 | 0 |
| DO218852 | ICGC-MELA | 80.7 | Living | 0 | 0 | 0 | 0 | 0 |
| DO218851 | ICGC-MELA | 59.8 | Deceased | 0 | 0 | 0 | 0 | 0 |
| DO218850 | ICGC-MELA | 23.5 | Deceased | 0 | 0 | 0 | 0 | 0 |
| DO218845 | ICGC-MELA | 60.5 | Living | 0 | 0 | 0 | 0 | 0 |
| DO218844 | ICGC-MELA | 64.7 | Deceased | 0 | 0 | 0 | 0 | 0 |
| DO218843 | ICGC-MELA | 22.2 | Deceased | 0 | 0 | 0 | 0 | 0 |
| DO218842 | ICGC-MELA | 123.2 | Living | 0 | 0 | 0 | 0 | 0 |
| DO218849 | ICGC-MELA | 29.1 | Deceased | 0 | 0 | 0 | 0 | 0 |
| DO218848 | ICGC-MELA | 28.6 | Deceased | 0 | 0 | 0 | 0 | 0 |
| DO218841 | ICGC-MELA | 13.3 | Deceased | 0 | 0 | 0 | 0 | 0 |
| DO218839 | ICGC-MELA | 60.7 | Living | 0 | 0 | 0 | 0 | 0 |
| DO218838 | ICGC-MELA | 27.7 | Deceased | 0 | 0 | 0 | 0 | 0 |
| DO218837 | ICGC-MELA | 70.5 | Living | 0 | 0 | 0 | 0 | 0 |
| DO229506 | ICGC-MELA | 33.0 | Living | 0 | 0 | 0 | 0 | 0 |
| DO229515 | ICGC-MELA | 38.5 | Living | 0 | 0 | 0 | 0 | 0 |
| DO229518 | ICGC-MELA | 20.6 | Deceased | 0 | 0 | 0 | 0 | 0 |
| DO229510 | ICGC-MELA | 28.8 | Living | 0 | 0 | 0 | 0 | 0 |
| DO218898 | ICGC-MELA | 40.3 | Living | 0 | 0 | 0 | 0 | 0 |
| DO218896 | ICGC-MELA | 68.5 | Living | 0 | 0 | 0 | 0 | 0 |
| DO218894 | ICGC-MELA | 176.9 | Deceased | 0 | 0 | 0 | 0 | 0 |
| DO229520 | ICGC-MELA | 10.7 | Deceased | 0 | 0 | 0 | 0 | 0 |
| DO218888 | ICGC-MELA | 13.2 | Deceased | 0 | 0 | 0 | 0 | 0 |
| DO218886 | ICGC-MELA | 65.3 | Deceased | 0 | 0 | 0 | 0 | 0 |
| DO218880 | ICGC-MELA | 60.6 | Living | 0 | 0 | 0 | 0 | 0 |
| DO218884 | ICGC-MELA | 31.7 | Living | 0 | 0 | 0 | 0 | 0 |
| DO218882 | ICGC-MELA | 51.9 | Living | 0 | 0 | 0 | 0 | 0 |
| DO227677 | ICGC-MELA | 17.7 | Deceased | 0 | 0 | 0 | 0 | 0 |
| DO227688 | ICGC-MELA | 92.3 | Living | 0 | 0 | 0 | 0 | 0 |
| DO227691 | ICGC-MELA | 36.1 | Deceased | 0 | 0 | 0 | 0 | 0 |
| DO222368 | ICGC-MELA | 56.2 | Deceased | 0 | 0 | 0 | 0 | 0 |
| DO222379 | ICGC-MELA | 2.3 | Living | 0 | 0 | 0 | 0 | 0 |
| DO222377 | ICGC-MELA | 31.8 | Living | 0 | 0 | 0 | 0 | 0 |
| DO222384 | ICGC-MELA | 123.2 | Living | 0 | 0 | 0 | 0 | 0 |
| DO222399 | ICGC-MELA | 44.1 | Deceased | 0 | 0 | 0 | 0 | 0 |
| DO222464 | ICGC-MELA | 38.6 | Living | 0 | 0 | 0 | 0 | 0 |
| DO222475 | ICGC-MELA | 50.2 | Deceased | 0 | 0 | 0 | 0 | 0 |
| DO222484 | ICGC-MELA | 44.5 | Deceased | 0 | 0 | 0 | 0 | 0 |
| DO222498 | ICGC-MELA | 96.9 | Living | 0 | 0 | 0 | 0 | 0 |
| DO222409 | ICGC-MELA | 18.2 | Living | 0 | 0 | 0 | 0 | 0 |
| DO222407 | ICGC-MELA | 56.1 | Living | 0 | 0 | 0 | 0 | 0 |
| DO222413 | ICGC-MELA | 41.2 | Living | 0 | 0 | 0 | 0 | 0 |
| DO222411 | ICGC-MELA | 40.0 | Living | 0 | 0 | 0 | 0 | 0 |
| DO222416 | ICGC-MELA | 0.0 | Living | 0 | 0 | 0 | 0 | 0 |
| DO222426 | ICGC-MELA | 62.4 | Deceased | 0 | 0 | 0 | 0 | 0 |
| DO222447 | ICGC-MELA | 17.0 | Living | 0 | 0 | 0 | 0 | 0 |
| DO222453 | ICGC-MELA | 21.3 | Deceased | 0 | 0 | 0 | 0 | 0 |
| DO222458 | ICGC-MELA | 19.1 | Living | 0 | 0 | 0 | 0 | 0 |
| DO222591 | ICGC-MELA | 81.5 | Living | 0 | 0 | 0 | 0 | 0 |
| DO222502 | ICGC-MELA | 40.1 | Living | 0 | 0 | 0 | 0 | 0 |
| DO222510 | ICGC-MELA | 18.2 | Deceased | 0 | 0 | 0 | 0 | 0 |
| DO222516 | ICGC-MELA | 29.2 | Deceased | 0 | 0 | 0 | 0 | 0 |
| DO222532 | ICGC-MELA | 27.6 | Deceased | 0 | 0 | 0 | 0 | 0 |
| DO222542 | ICGC-MELA | 5.4 | Deceased | 0 | 0 | 0 | 0 | 0 |
| DO222554 | ICGC-MELA | 37.3 | Deceased | 0 | 0 | 0 | 0 | 0 |
| DO222567 | ICGC-MELA | 36.6 | Living | 0 | 0 | 0 | 0 | 0 |
| DO222562 | ICGC-MELA | 0.0 | Living | 0 | 0 | 0 | 0 | 0 |
| DO222578 | ICGC-MELA | 35.1 | Deceased | 0 | 0 | 0 | 0 | 0 |
| DO222572 | ICGC-MELA | 0.0 |  | 0 | 0 | 0 | 0 | 0 |
| DO222628 | ICGC-MELA | 17.2 | Deceased | 0 | 0 | 0 | 0 | 0 |
| DO222635 | ICGC-MELA | 140.7 | Deceased | 0 | 0 | 0 | 0 | 0 |
| DO222647 | ICGC-MELA | 12.1 | Living | 0 | 0 | 0 | 0 | 0 |
| DO222670 | ICGC-MELA | 124.4 | Living | 0 | 0 | 0 | 0 | 0 |
| DO222699 | ICGC-MELA | 11.7 | Deceased | 0 | 0 | 0 | 0 | 0 |
| DO222696 | ICGC-MELA | 50.5 | Living | 0 | 0 | 0 | 0 | 0 |
| DO222694 | ICGC-MELA | 18.6 | Deceased | 0 | 0 | 0 | 0 | 0 |
| DO222619 | ICGC-MELA | 20.0 | Deceased | 0 | 0 | 0 | 0 | 0 |
| DO222766 | ICGC-MELA | 16.9 | Deceased | 0 | 0 | 0 | 0 | 0 |
| DO222738 | ICGC-MELA | 13.7 | Deceased | 0 | 0 | 0 | 0 | 0 |
| DO222898 | ICGC-MELA | 188.3 | Living | 0 | 0 | 0 | 0 | 0 |
| DO222802 | ICGC-MELA | 15.4 | Deceased | 0 | 0 | 0 | 0 | 0 |
| DO222810 | ICGC-MELA | 31.3 | Deceased | 0 | 0 | 0 | 0 | 0 |
| DO222818 | ICGC-MELA | 4.1 | Deceased | 0 | 0 | 0 | 0 | 0 |
| DO222820 | ICGC-MELA | 21.7 | Deceased | 0 | 0 | 0 | 0 | 0 |
| DO222824 | ICGC-MELA | 9.3 | Deceased | 0 | 0 | 0 | 0 | 0 |
| DO222832 | ICGC-MELA | 54.1 | Living | 0 | 0 | 0 | 0 | 0 |
| DO222859 | ICGC-MELA | 17.0 | Deceased | 0 | 0 | 0 | 0 | 0 |
| DO222904 | ICGC-MELA | 37.9 | Living | 0 | 0 | 0 | 0 | 0 |
| DO222914 | ICGC-MELA | 28.3 | Living | 0 | 0 | 0 | 0 | 0 |
| DO222911 | ICGC-MELA | 3.5 | Deceased | 0 | 0 | 0 | 0 | 0 |
| DO220856 | ICGC-MELA | 55.1 | Deceased | 0.948 | 0 | 0 | 1 | 0 |
| DO220884 | ICGC-MELA | 93.3 | Deceased | 0.948 | 0 | 0 | 1 | 0 |
| DO222504 | ICGC-MELA | 87.0 | Living | 0.948 | 0 | 0 | 1 | 0 |
| DO222521 | ICGC-MELA | 21.1 | Deceased | 0.948 | 0 | 0 | 1 | 0 |
| DO222536 | ICGC-MELA | 37.9 | Deceased | 0.948 | 0 | 0 | 1 | 0 |
| DO222691 | ICGC-MELA | 22.0 | Deceased | 0.948 | 0 | 0 | 1 | 0 |
| DO222747 | ICGC-MELA | 260.5 | Deceased | 0.948 | 0 | 0 | 1 | 0 |
| DO222708 | ICGC-MELA | 20.8 | Deceased | 0.948 | 0 | 0 | 1 | 0 |
| DO222727 | ICGC-MELA | 80.1 | Deceased | 0.948 | 0 | 0 | 1 | 0 |
| DO220842 | ICGC-MELA | 82.1 | Deceased | 1.234 | 1 | 0 | 0 | 0 |
| DO220855 | ICGC-MELA | 144.8 | Deceased | 1.234 | 1 | 0 | 0 | 0 |
| DO220861 | ICGC-MELA | 10.4 | Deceased | 1.234 | 1 | 0 | 0 | 0 |
| DO220873 | ICGC-MELA | 135.0 | Living | 1.234 | 1 | 0 | 0 | 0 |
| DO220872 | ICGC-MELA | 8.8 | Deceased | 1.234 | 1 | 0 | 0 | 0 |
| DO220876 | ICGC-MELA | 347.9 | Deceased | 1.234 | 1 | 0 | 0 | 0 |
| DO220883 | ICGC-MELA | 99.8 | Deceased | 1.234 | 1 | 0 | 0 | 0 |
| DO220880 | ICGC-MELA | 124.8 | Deceased | 1.234 | 1 | 0 | 0 | 0 |
| DO220889 | ICGC-MELA | 16.2 | Deceased | 1.234 | 1 | 0 | 0 | 0 |
| DO219873 | ICGC-MELA | 40.2 | Deceased | 1.234 | 1 | 0 | 0 | 0 |
| DO228011 | ICGC-MELA | 63.0 | Living | 1.234 | 1 | 0 | 0 | 0 |
| DO228014 | ICGC-MELA | 52.8 | Living | 1.234 | 1 | 0 | 0 | 0 |
| DO220910 | ICGC-MELA | 154.5 | Living | 1.234 | 1 | 0 | 0 | 0 |
| DO229465 | ICGC-MELA | 27.6 | Deceased | 1.234 | 1 | 0 | 0 | 0 |
| DO218900 | ICGC-MELA | 17.1 | Deceased | 1.234 | 1 | 0 | 0 | 0 |
| DO218857 | ICGC-MELA | 112.8 | Living | 1.234 | 1 | 0 | 0 | 0 |
| DO218846 | ICGC-MELA | 37.5 | Deceased | 1.234 | 1 | 0 | 0 | 0 |
| DO222382 | ICGC-MELA | 13.8 | Deceased | 1.234 | 1 | 0 | 0 | 0 |
| DO222395 | ICGC-MELA | 25.4 | Living | 1.234 | 1 | 0 | 0 | 0 |
| DO222396 | ICGC-MELA | 84.9 | Living | 1.234 | 1 | 0 | 0 | 0 |
| DO222494 | ICGC-MELA | 29.4 | Deceased | 1.234 | 1 | 0 | 0 | 0 |
| DO222405 | ICGC-MELA | 44.6 | Living | 1.234 | 1 | 0 | 0 | 0 |
| DO222429 | ICGC-MELA | 219.7 | Deceased | 1.234 | 1 | 0 | 0 | 0 |
| DO222438 | ICGC-MELA | 48.2 | Deceased | 1.234 | 1 | 0 | 0 | 0 |
| DO222514 | ICGC-MELA | 131.1 | Living | 1.234 | 1 | 0 | 0 | 0 |
| DO222527 | ICGC-MELA | 42.1 | Deceased | 1.234 | 1 | 0 | 0 | 0 |
| DO222550 | ICGC-MELA | 151.7 | Living | 1.234 | 1 | 0 | 0 | 0 |
| DO222638 | ICGC-MELA | 9.4 | Deceased | 1.234 | 1 | 0 | 0 | 0 |
| DO222658 | ICGC-MELA | 5.0 | Deceased | 1.234 | 1 | 0 | 0 | 0 |
| DO222678 | ICGC-MELA | 170.2 | Deceased | 1.234 | 1 | 0 | 0 | 0 |
| DO222683 | ICGC-MELA | 60.1 | Living | 1.234 | 1 | 0 | 0 | 0 |
| DO222721 | ICGC-MELA | 24.8 | Living | 1.234 | 1 | 0 | 0 | 0 |
| DO220853 | ICGC-MELA | 233.3 | Deceased | 1.636 | 0 | 0 | 0 | 1 |
| DO220874 | ICGC-MELA | 152.2 | Deceased | 1.636 | 0 | 0 | 0 | 1 |
| DO220890 | ICGC-MELA | 22.1 | Deceased | 1.636 | 0 | 0 | 0 | 1 |
| DO220898 | ICGC-MELA | 76.9 | Living | 1.636 | 0 | 0 | 0 | 1 |
| DO219879 | ICGC-MELA | 31.2 | Deceased | 1.636 | 0 | 0 | 0 | 1 |
| DO220905 | ICGC-MELA | 8.3 | Deceased | 1.636 | 0 | 0 | 0 | 1 |
| DO220913 | ICGC-MELA | 17.1 | Deceased | 1.636 | 0 | 0 | 0 | 1 |
| DO218912 | ICGC-MELA | 45.0 | Deceased | 1.636 | 0 | 0 | 0 | 1 |
| DO218847 | ICGC-MELA | 24.5 | Deceased | 1.636 | 0 | 0 | 0 | 1 |
| DO218890 | ICGC-MELA | 50.9 | Deceased | 1.636 | 0 | 0 | 0 | 1 |
| DO222481 | ICGC-MELA | 10.3 | Living | 1.636 | 0 | 0 | 0 | 1 |
| DO222402 | ICGC-MELA | 44.1 | Deceased | 1.636 | 0 | 0 | 0 | 1 |
| DO222415 | ICGC-MELA | 25.4 | Living | 1.636 | 0 | 0 | 0 | 1 |
| DO222615 | ICGC-MELA | 160.8 | Living | 1.636 | 0 | 0 | 0 | 1 |
| DO222749 | ICGC-MELA | 67.1 | Deceased | 1.636 | 0 | 0 | 0 | 1 |
| DO220907 | ICGC-MELA | 39.2 | Living | 1.64 | 0 | 1 | 0 | 0 |
| DO220911 | ICGC-MELA | 133.8 | Deceased | 1.64 | 0 | 1 | 0 | 0 |
| DO218892 | ICGC-MELA | 113.4 | Deceased | 1.64 | 0 | 1 | 0 | 0 |
| DO222359 | ICGC-MELA | 0.8 | Living | 1.64 | 0 | 1 | 0 | 0 |
| DO222470 | ICGC-MELA | 5.2 | Living | 1.64 | 0 | 1 | 0 | 0 |
| DO222491 | ICGC-MELA | 272.5 | Deceased | 1.64 | 0 | 1 | 0 | 0 |
| DO222457 | ICGC-MELA | 49.5 | Living | 1.64 | 0 | 1 | 0 | 0 |
| DO222546 | ICGC-MELA | 125.7 | Living | 1.64 | 0 | 1 | 0 | 0 |
| DO222743 | ICGC-MELA | 42.3 | Living | 1.64 | 0 | 1 | 0 | 0 |
| DO220857 | ICGC-MELA | 116.0 | Living | 2.182 | 1 | 0 | 1 | 0 |
| DO220862 | ICGC-MELA | 222.4 | Living | 2.182 | 1 | 0 | 1 | 0 |
| DO220875 | ICGC-MELA | 87.1 | Deceased | 2.182 | 1 | 0 | 1 | 0 |
| DO220902 | ICGC-MELA | 19.8 | Deceased | 2.182 | 1 | 0 | 1 | 0 |
| DO220906 | ICGC-MELA | 28.1 | Deceased | 2.182 | 1 | 0 | 1 | 0 |
| DO218874 | ICGC-MELA | 116.7 | Deceased | 2.182 | 1 | 0 | 1 | 0 |
| DO218863 | ICGC-MELA | 19.2 | Deceased | 2.182 | 1 | 0 | 1 | 0 |
| DO222390 | ICGC-MELA | 113.6 | Living | 2.182 | 1 | 0 | 1 | 0 |
| DO222418 | ICGC-MELA | 7.1 | Living | 2.182 | 1 | 0 | 1 | 0 |
| DO222595 | ICGC-MELA | 64.5 | Living | 2.182 | 1 | 0 | 1 | 0 |
| DO222557 | ICGC-MELA | 134.3 | Deceased | 2.182 | 1 | 0 | 1 | 0 |
| DO222644 | ICGC-MELA | 141.9 | Deceased | 2.182 | 1 | 0 | 1 | 0 |
| DO222710 | ICGC-MELA | 36.4 | Deceased | 2.182 | 1 | 0 | 1 | 0 |
| DO222847 | ICGC-MELA | 36.5 | Living | 2.182 | 1 | 0 | 1 | 0 |
| DO218861 | ICGC-MELA | 23.8 | Deceased | 2.588 | 0 | 1 | 1 | 0 |
| DO218840 | ICGC-MELA | 80.9 | Deceased | 2.588 | 0 | 1 | 1 | 0 |
| DO220885 | ICGC-MELA | 132.6 | Living | 2.87 | 1 | 0 | 0 | 1 |
| DO220893 | ICGC-MELA | 10.3 | Living | 2.87 | 1 | 0 | 0 | 1 |
| DO229502 | ICGC-MELA | 12.0 | Deceased | 2.87 | 1 | 0 | 0 | 1 |
| DO222599 | ICGC-MELA | 2.6 | Deceased | 2.87 | 1 | 0 | 0 | 1 |
| DO222775 | ICGC-MELA | 148.6 | Deceased | 2.87 | 1 | 0 | 0 | 1 |
| DO219871 | ICGC-MELA | 57.0 | Living | 2.874 | 1 | 1 | 0 | 0 |
| DO220900 | ICGC-MELA | 8.9 | Deceased | 2.874 | 1 | 1 | 0 | 0 |
| DO229494 | ICGC-MELA | 59.8 | Deceased | 2.874 | 1 | 1 | 0 | 0 |
| DO229462 | ICGC-MELA | 44.6 | Deceased | 2.874 | 1 | 1 | 0 | 0 |
| DO218878 | ICGC-MELA | 36.3 | Living | 2.874 | 1 | 1 | 0 | 0 |
| DO222420 | ICGC-MELA | 16.3 | Living | 2.874 | 1 | 1 | 0 | 0 |
| DO222662 | ICGC-MELA | 1.2 | Living | 2.874 | 1 | 1 | 0 | 0 |
| DO222714 | ICGC-MELA | 14.1 | Deceased | 2.874 | 1 | 1 | 0 | 0 |
| DO222731 | ICGC-MELA | 47.3 | Deceased | 2.874 | 1 | 1 | 0 | 0 |
| DO222887 | ICGC-MELA | 40.1 | Living | 2.874 | 1 | 1 | 0 | 0 |
| DO220852 | ICGC-MELA | 101.6 | Deceased | 3.276 | 0 | 1 | 0 | 1 |
| DO220882 | ICGC-MELA | 19.9 | Deceased | 3.276 | 0 | 1 | 0 | 1 |
| DO219881 | ICGC-MELA | 99.6 | Living | 3.276 | 0 | 1 | 0 | 1 |
| DO227681 | ICGC-MELA | 5.2 | Deceased | 3.276 | 0 | 1 | 0 | 1 |
| DO220878 | ICGC-MELA | 137.9 | Living | 3.818 | 1 | 0 | 1 | 1 |
| DO229446 | ICGC-MELA | 52.3 | Deceased | 3.818 | 1 | 0 | 1 | 1 |
| DO222843 | ICGC-MELA | 30.2 | Deceased | 3.818 | 1 | 0 | 1 | 1 |
| DO220886 | ICGC-MELA | 257.7 | Living | 3.822 | 1 | 1 | 1 | 0 |
| DO220891 | ICGC-MELA | 44.1 | Living | 3.822 | 1 | 1 | 1 | 0 |
| DO219878 | ICGC-MELA | 41.0 | Deceased | 3.822 | 1 | 1 | 1 | 0 |
| DO229459 | ICGC-MELA | 57.2 | Living | 3.822 | 1 | 1 | 1 | 0 |
| DO229453 | ICGC-MELA | 23.6 | Deceased | 3.822 | 1 | 1 | 1 | 0 |
| DO229456 | ICGC-MELA | 9.7 | Deceased | 3.822 | 1 | 1 | 1 | 0 |
| DO222632 | ICGC-MELA | 50.9 | Living | 3.822 | 1 | 1 | 1 | 0 |
| DO222880 | ICGC-MELA | 63.8 | Deceased | 3.822 | 1 | 1 | 1 | 0 |
| DO220851 | ICGC-MELA | 38.7 | Living | 4.51 | 1 | 1 | 0 | 1 |
| DO220899 | ICGC-MELA | 342.5 | Deceased | 4.51 | 1 | 1 | 0 | 1 |
| DO220901 | ICGC-MELA | 282.6 | Living | 4.51 | 1 | 1 | 0 | 1 |
| DO220903 | ICGC-MELA | 41.2 | Living | 4.51 | 1 | 1 | 0 | 1 |
| DO222653 | ICGC-MELA | 74.2 | Living | 4.51 | 1 | 1 | 0 | 1 |
| DO222753 | ICGC-MELA | 25.2 | Deceased | 4.51 | 1 | 1 | 0 | 1 |
| DO222868 | ICGC-MELA | 3.3 | Living | 4.51 | 1 | 1 | 0 | 1 |
| DO222837 | ICGC-MELA | 37.0 | Deceased | 4.51 | 1 | 1 | 0 | 1 |
| DO222908 | ICGC-MELA | 5.4 | Living | 4.51 | 1 | 1 | 0 | 1 |
| DO220877 | ICGC-MELA | 14.2 | Living | 5.458 | 1 | 1 | 1 | 1 |
| DO220912 | ICGC-MELA | 85.8 | Deceased | 5.458 | 1 | 1 | 1 | 1 |
| DO229470 | ICGC-MELA | 12.1 | Deceased | 5.458 | 1 | 1 | 1 | 1 |
| DO222363 | ICGC-MELA | 5.1 | Deceased | 5.458 | 1 | 1 | 1 | 1 |
| DO222423 | ICGC-MELA | 22.0 | Deceased | 5.458 | 1 | 1 | 1 | 1 |
| DO222434 | ICGC-MELA | 47.9 | Deceased | 5.458 | 1 | 1 | 1 | 1 |
| DO222702 | ICGC-MELA | 30.0 | Deceased | 5.458 | 1 | 1 | 1 | 1 |
| DO222875 | ICGC-MELA | 39.7 | Living | 5.458 | 1 | 1 | 1 | 1 |
