# Supplementary material for: Integrated genomic analysis identifies a genetic mutation model predicting response to immune checkpoint inhibitors in melanoma
Source: Cancer Med. 2020 Sep 24;9(22):8498–518. doi: 10.1002/cam4.3481 (PMC7666739; doi:10.1002/cam4.3481)
Supplement: Supplementary file 11 — Table S3 [file CAM4-9-8498-s011.docx]

| **Table S3. The detailed clinical and genomic characteristics of melanoma patients with ICIs therapy in the Hellmann cohort and Miao cohort** | | | | | | | | | | | |
| --- | --- | --- | --- | --- | --- | --- | --- | --- | --- | --- | --- |
| ID | Cohort | Histology | Best objective response | PFS time (months) | PFS status | Clinical benefit | ITS | *THSD7B* | *SYNE2* | *GRM3* | *FLNC* |
| AL4602_T_N | Miao | Non-Squamous | SD | 8.5 | Censored | DCB | 0 | 0 | 0 | 0 | 0 |
| AU5884_T_N | Miao | Non-Squamous | PD | 1.8 | Progressed | NCB | 0 | 0 | 0 | 0 | 0 |
| BL3403_T_N | Miao | Non-Squamous | SD | 6.6 | Progressed | DCB | 0 | 0 | 0 | 0 | 0 |
| CA9903_T_N | Miao | Non-Squamous | PR | 14.7 | Progressed | DCB | 2.584 | 0 | 0 | 1 | 1 |
| CANSEQU01-0100425-TP-NB-SM-6ZL67-SM-6ZL66 | Miao | Non-Squamous | PR | 6.9 | Progressed | DCB | 0 | 0 | 0 | 0 | 0 |
| DI6359_T_N | Miao | Non-Squamous | PR | 9.9 | Censored | DCB | 0 | 0 | 0 | 0 | 0 |
| DM123062_T_N | Miao | Non-Squamous | PD | 1.9 | Progressed | NCB | 0 | 0 | 0 | 0 | 0 |
| FR9547_T_N | Miao | Non-Squamous | PR | 4.2 | Censored | DCB | 0 | 0 | 0 | 0 | 0 |
| HE3202_T_N | Miao | Non-Squamous | PR | 14.9 | Censored | DCB | 2.87 | 1 | 0 | 0 | 1 |
| JB112852_T_N | Miao | Non-Squamous | PD | 3.3 | Progressed | NCB | 0 | 0 | 0 | 0 | 0 |
| KA3947_T_N | Miao | Non-Squamous | SD | 8.2 | Progressed | DCB | 0 | 0 | 0 | 0 | 0 |
| LO3793_T_N | Miao | Non-Squamous | SD | 3.5 | Progressed | NCB | 0 | 0 | 0 | 0 | 0 |
| LO5004_T_N | Miao | Non-Squamous | SD | 6.4 | Progressed | DCB | 0 | 0 | 0 | 0 | 0 |
| LUAD-BS-08-013532-TP-NT-SM-9J2Y1-SM-9J2Y2 | Miao | Non-Squamous | PR | 8.1 | Progressed | DCB | 0 | 0 | 0 | 0 | 0 |
| LUAD-BS-10-R22401-TP-NB-SM-9J2XK-SM-9HBZR | Miao | Non-Squamous | SD | 1.7 | Progressed | NCB | 0 | 0 | 0 | 0 | 0 |
| LUAD-BS-11-F1305-TP-NT-SM-9J2XG-SM-9J2XH | Miao | Non-Squamous | SD | 1.2 | Progressed | NCB | 0 | 0 | 0 | 0 | 0 |
| LUAD-BS-12-R10269-TP-NB-SM-9J2XO-SM-9HBZT | Miao | Non-Squamous | PR | 4.8 | Progressed | DCB | 0 | 0 | 0 | 0 | 0 |
| LUAD-BS-12-X27523-TP-NT-SM-9J2Y5-SM-9J2Y6 | Miao | Non-Squamous | SD | 4.2 | Progressed | NCB | 0 | 0 | 0 | 0 | 0 |
| LUAD-BS-13-F33496-TP-NB-SM-9J2XU-SM-9HBZX | Miao | Non-Squamous | SD | 3.3 | Progressed | NCB | 0 | 0 | 0 | 0 | 0 |
| LUAD-BS-13-J60666-TP-NB-SM-9J2YL-SM-9HBZW | Miao | Non-Squamous | PR | 33.3 | Progressed | DCB | 0 | 0 | 0 | 0 | 0 |
| LUAD-BS-13-N35728-TP-NB-SM-9J2XI-SM-9HBZQ | Miao | Non-Squamous | SD | 6.5 | Progressed | DCB | 0 | 0 | 0 | 0 | 0 |
| LUAD-BS-14-J45174-TP-NT-SM-9J2YJ-SM-9J2YK | Miao | Non-Squamous | PD | 1.3 | Progressed | NCB | 0 | 0 | 0 | 0 | 0 |
| LUAD-BS-14-N19784-TP-NT-SM-9J2XW-SM-9J2XX | Miao | Non-Squamous | SD | 4.2 | Progressed | NCB | 1.234 | 1 | 0 | 0 | 0 |
| LUNG-2630-TP-NB-SM-CLFOY-SM-AV34W | Miao | Non-Squamous | SD | 7.8 | Progressed | DCB | 1.64 | 0 | 1 | 0 | 0 |
| Lung-DFCI-11-104-009-TM-NB-SM-5YS7O-SM-5YS7P | Miao | Non-Squamous | CR | 22.0 | Censored | DCB | 3.818 | 1 | 0 | 1 | 1 |
| M4945_T_N | Miao | Non-Squamous | PR | 14.8 | Censored | DCB | 0 | 0 | 0 | 0 | 0 |
| MA7027_T_N | Miao | Non-Squamous | PD | 1.8 | Progressed | NCB | 1.636 | 0 | 0 | 0 | 1 |
| NI9507_T_N | Miao | Non-Squamous | PD | 1.9 | Progressed | NCB | 0 | 0 | 0 | 0 | 0 |
| nsclc_mskcc_20181 | Hellmann | Non-Squamous | PD | 1.4 | Progressed | NCB | 0 | 0 | 0 | 0 | 0 |
| nsclc_mskcc_201811 | Hellmann | Non-Squamous | SD | 7.8 | Progressed | DCB | 0 | 0 | 0 | 0 | 0 |
| nsclc_mskcc_201812 | Hellmann | Non-Squamous | PR | 26.2 | Censored | DCB | 0 | 0 | 0 | 0 | 0 |
| nsclc_mskcc_201813 | Hellmann | Non-Squamous | SD | 24.0 | Progressed | DCB | 0 | 0 | 0 | 0 | 0 |
| nsclc_mskcc_201814 | Hellmann | Non-Squamous | PD | 1.7 | Progressed | NCB | 0 | 0 | 0 | 0 | 0 |
| nsclc_mskcc_201815 | Hellmann | Non-Squamous | PD | 0.0 | Censored | NCB | 0 | 0 | 0 | 0 | 0 |
| nsclc_mskcc_201816 | Hellmann | Non-Squamous | SD | 3.6 | Progressed | NCB | 0 | 0 | 0 | 0 | 0 |
| nsclc_mskcc_201817 | Hellmann | Non-Squamous | SD | 12.1 | Progressed | DCB | 0 | 0 | 0 | 0 | 0 |
| nsclc_mskcc_201818 | Hellmann | Non-Squamous | PR | 29.7 | Censored | DCB | 2.87 | 1 | 0 | 0 | 1 |
| nsclc_mskcc_201819 | Hellmann | Non-Squamous | PR | 28.8 | Censored | DCB | 3.276 | 0 | 1 | 0 | 1 |
| nsclc_mskcc_201820 | Hellmann | Non-Squamous | PD | 1.4 | Progressed | NCB | 0 | 0 | 0 | 0 | 0 |
| nsclc_mskcc_201821 | Hellmann | Non-Squamous | CR | 21.6 | Censored | DCB | 0 | 0 | 0 | 0 | 0 |
| nsclc_mskcc_201822 | Hellmann | Non-Squamous | PD | 2.5 | Progressed | NCB | 1.636 | 0 | 0 | 0 | 1 |
| nsclc_mskcc_201823 | Hellmann | Non-Squamous | PR | 8.0 | Progressed | DCB | 1.636 | 0 | 0 | 0 | 1 |
| nsclc_mskcc_201824 | Hellmann | Non-Squamous | PD | 2.3 | Progressed | NCB | 0 | 0 | 0 | 0 | 0 |
| nsclc_mskcc_201825 | Hellmann | Non-Squamous | SD | 22.1 | Progressed | DCB | 0 | 0 | 0 | 0 | 0 |
| nsclc_mskcc_201826 | Hellmann | Non-Squamous | PR | 23.0 | Censored | DCB | 4.51 | 1 | 1 | 0 | 1 |
| nsclc_mskcc_201827 | Hellmann | Non-Squamous | SD | 4.1 | Progressed | NCB | 0 | 0 | 0 | 0 | 0 |
| nsclc_mskcc_201828 | Hellmann | Non-Squamous | SD | 17.1 | Progressed | DCB | 1.234 | 1 | 0 | 0 | 0 |
| nsclc_mskcc_201829 | Hellmann | Non-Squamous | PD | 2.6 | Progressed | NCB | 0 | 0 | 0 | 0 | 0 |
| nsclc_mskcc_20183 | Hellmann | Non-Squamous | SD | 1.5 | Censored | NCB | 0 | 0 | 0 | 0 | 0 |
| nsclc_mskcc_201831 | Hellmann | Non-Squamous | PD | 2.3 | Progressed | NCB | 0 | 0 | 0 | 0 | 0 |
| nsclc_mskcc_201832 | Hellmann | Non-Squamous | CR | 19.0 | Censored | DCB | 0 | 0 | 0 | 0 | 0 |
| nsclc_mskcc_201834 | Hellmann | Non-Squamous | PR | 22.3 | Censored | DCB | 0 | 0 | 0 | 0 | 0 |
| nsclc_mskcc_201835 | Hellmann | Non-Squamous | PD | 2.3 | Progressed | NCB | 0 | 0 | 0 | 0 | 0 |
| nsclc_mskcc_201836 | Hellmann | Non-Squamous | SD | 13.3 | Progressed | DCB | 0 | 0 | 0 | 0 | 0 |
| nsclc_mskcc_201837 | Hellmann | Non-Squamous | PR | 19.0 | Censored | DCB | 0 | 0 | 0 | 0 | 0 |
| nsclc_mskcc_201838 | Hellmann | Non-Squamous | PR | 7.8 | Progressed | DCB | 1.636 | 0 | 0 | 0 | 1 |
| nsclc_mskcc_201839 | Hellmann | Non-Squamous | NA | 0.0 | Censored | NCB | 0 | 0 | 0 | 0 | 0 |
| nsclc_mskcc_201840 | Hellmann | Non-Squamous | PR | 8.1 | Progressed | DCB | 0 | 0 | 0 | 0 | 0 |
| nsclc_mskcc_201841 | Hellmann | Non-Squamous | CR | 13.5 | Censored | DCB | 2.87 | 1 | 0 | 0 | 1 |
| nsclc_mskcc_201842 | Hellmann | Non-Squamous | SD | 3.9 | Progressed | NCB | 0 | 0 | 0 | 0 | 0 |
| nsclc_mskcc_201843 | Hellmann | Non-Squamous | SD | 5.1 | Progressed | NCB | 2.87 | 1 | 0 | 0 | 1 |
| nsclc_mskcc_201849 | Hellmann | Non-Squamous | SD | 11.7 | Progressed | DCB | 0 | 0 | 0 | 0 | 0 |
| nsclc_mskcc_20185 | Hellmann | Non-Squamous | SD | 7.6 | Progressed | DCB | 0 | 0 | 0 | 0 | 0 |
| nsclc_mskcc_201851 | Hellmann | Non-Squamous | PR | 29.9 | Censored | DCB | 1.64 | 0 | 1 | 0 | 0 |
| nsclc_mskcc_201852 | Hellmann | Non-Squamous | PR | 30.3 | Censored | DCB | 0 | 0 | 0 | 0 | 0 |
| nsclc_mskcc_201853 | Hellmann | Non-Squamous | PD | 1.4 | Progressed | NCB | 0 | 0 | 0 | 0 | 0 |
| nsclc_mskcc_201854 | Hellmann | Non-Squamous | SD | 5.2 | Progressed | NCB | 0 | 0 | 0 | 0 | 0 |
| nsclc_mskcc_201855 | Hellmann | Non-Squamous | PD | 2.1 | Progressed | NCB | 0 | 0 | 0 | 0 | 0 |
| nsclc_mskcc_201856 | Hellmann | Non-Squamous | SD | 6.5 | Progressed | DCB | 2.87 | 1 | 0 | 0 | 1 |
| nsclc_mskcc_201857 | Hellmann | Non-Squamous | PD | 2.0 | Progressed | NCB | 0 | 0 | 0 | 0 | 0 |
| nsclc_mskcc_201858 | Hellmann | Non-Squamous | NA | 1.9 | Progressed | NCB | 1.636 | 0 | 0 | 0 | 1 |
| nsclc_mskcc_201859 | Hellmann | Non-Squamous | PD | 2.3 | Progressed | NCB | 0 | 0 | 0 | 0 | 0 |
| nsclc_mskcc_20186 | Hellmann | Non-Squamous | SD | 3.7 | Progressed | NCB | 0 | 0 | 0 | 0 | 0 |
| nsclc_mskcc_201860 | Hellmann | Non-Squamous | PR | 24.3 | Censored | DCB | 0 | 0 | 0 | 0 | 0 |
| nsclc_mskcc_201861 | Hellmann | Non-Squamous | NA | 1.7 | Progressed | NCB | 1.64 | 0 | 1 | 0 | 0 |
| nsclc_mskcc_201862 | Hellmann | Non-Squamous | PR | 23.0 | Progressed | DCB | 2.874 | 1 | 1 | 0 | 0 |
| nsclc_mskcc_201864 | Hellmann | Non-Squamous | PR | 20.0 | Censored | DCB | 0 | 0 | 0 | 0 | 0 |
| nsclc_mskcc_201865 | Hellmann | Non-Squamous | SD | 4.8 | Censored | NCB | 0 | 0 | 0 | 0 | 0 |
| nsclc_mskcc_201868 | Hellmann | Non-Squamous | SD | 8.0 | Progressed | DCB | 1.234 | 1 | 0 | 0 | 0 |
| nsclc_mskcc_201869 | Hellmann | Non-Squamous | PD | 2.6 | Progressed | NCB | 0 | 0 | 0 | 0 | 0 |
| nsclc_mskcc_201871 | Hellmann | Non-Squamous | SD | 3.5 | Progressed | NCB | 0 | 0 | 0 | 0 | 0 |
| nsclc_mskcc_201872 | Hellmann | Non-Squamous | PD | 2.3 | Progressed | NCB | 0 | 0 | 0 | 0 | 0 |
| nsclc_mskcc_201873 | Hellmann | Non-Squamous | PR | 16.5 | Censored | DCB | 1.636 | 0 | 0 | 0 | 1 |
| nsclc_mskcc_201874 | Hellmann | Non-Squamous | SD | 8.0 | Progressed | DCB | 1.636 | 0 | 0 | 0 | 1 |
| nsclc_mskcc_201875 | Hellmann | Non-Squamous | CR | 14.9 | Censored | DCB | 0 | 0 | 0 | 0 | 0 |
| nsclc_mskcc_20188 | Hellmann | Non-Squamous | SD | 3.0 | Progressed | NCB | 2.87 | 1 | 0 | 0 | 1 |
| nsclc_mskcc_20189 | Hellmann | Non-Squamous | SD | 7.8 | Progressed | DCB | 0 | 0 | 0 | 0 | 0 |
| R7495_T_N | Miao | Non-Squamous | PD | 1.4 | Progressed | NCB | 1.636 | 0 | 0 | 0 | 1 |
| RH090935_T_N | Miao | Non-Squamous | PR | 10.5 | Censored | DCB | 0 | 0 | 0 | 0 | 0 |
| RI1933_T_N | Miao | Non-Squamous | PR | 16.8 | Censored | DCB | 1.636 | 0 | 0 | 0 | 1 |
| RO3338_T_N | Miao | Non-Squamous | PD | 2.1 | Progressed | NCB | 0 | 0 | 0 | 0 | 0 |
| SC0899_T_N | Miao | Non-Squamous | PR | 12.8 | Censored | DCB | 0 | 0 | 0 | 0 | 0 |
| SC6470_T_N | Miao | Non-Squamous | SD | 8.4 | Progressed | DCB | 0 | 0 | 0 | 0 | 0 |
| SU2C_Lung-SU2C-DFCI-LUAD-1002-TP-NB-SM-AOL41-SM-A46NE | Miao | Non-Squamous | PD | 1.9 | Progressed | NCB | 0 | 0 | 0 | 0 | 0 |
| SU2C_Lung-SU2C-DFCI-LUAD-1003-TP-NB-SM-AOL4D-SM-A46NF | Miao | Non-Squamous | PD | 1.9 | Progressed | NCB | 0 | 0 | 0 | 0 | 0 |
| SU2C_Lung-SU2C-DFCI-LUAD-1004-TP-NB-SM-AOL4P-SM-A46NG | Miao | Non-Squamous | SD | 3.7 | Progressed | NCB | 1.64 | 0 | 1 | 0 | 0 |
| SU2C_Lung-SU2C-DFCI-LUAD-1005-TP-NB-SM-AOL52-SM-A46NH | Miao | Non-Squamous | PD | 1.1 | Progressed | NCB | 0 | 0 | 0 | 0 | 0 |
| SU2C_Lung-SU2C-DFCI-LUAD-1006-TP-NB-SM-AOL5E-SM-A46NI | Miao | Non-Squamous | PD | 6.1 | Progressed | NCB | 0 | 0 | 0 | 0 | 0 |
| SU2C_Lung-SU2C-DFCI-LUAD-1010-TM-NB-SM-AOL6S-SM-A46NM | Miao | Non-Squamous | SD | 4.0 | Progressed | NCB | 0 | 0 | 0 | 0 | 0 |
| SU2C_Lung-SU2C-DFCI-LUAD-1011-TM-NB-SM-AOL75-SM-A46NN | Miao | Non-Squamous | PD | 1.9 | Progressed | NCB | 0 | 0 | 0 | 0 | 0 |
| SU2C_Lung-SU2C-DFCI-LUAD-1013-TP-NB-SM-AOL7V-SM-A46NP | Miao | Non-Squamous | PD | 1.1 | Progressed | NCB | 0 | 0 | 0 | 0 | 0 |
| SU2C_Lung-SU2C-DFCI-LUAD-1017-TM-NB-SM-AOL99-SM-A46NT | Miao | Non-Squamous | PR | 14.4 | Censored | DCB | 0.948 | 0 | 0 | 1 | 0 |
| SU2C_Lung-SU2C-DFCI-LUAD-1018-TP-NB-SM-AOL9H-SM-A46NU | Miao | Non-Squamous | PD | 1.2 | Progressed | NCB | 0 | 0 | 0 | 0 | 0 |
| SU2C_Lung-SU2C-DFCI-LUAD-1020-TM-NB-SM-AOL3C-SM-A46NC | Miao | Non-Squamous | PR | 29.9 | Censored | DCB | 0 | 0 | 0 | 0 | 0 |
| WA7899_T_N | Miao | Non-Squamous | PD | 1.9 | Progressed | NCB | 0 | 0 | 0 | 0 | 0 |
| Y2087_T_N | Miao | Non-Squamous | SD | 8.4 | Progressed | DCB | 0 | 0 | 0 | 0 | 0 |
| LUAD-BS-13-X14864-TP-NB-SM-9J2XQ-SM-9HBZU | Miao | NSCLC NOS | SD | 5.7 | Progressed | NCB | 0 | 0 | 0 | 0 | 0 |
| SA9755_T_N | Miao | NSCLC NOS | PR | 8.5 | Censored | DCB | 1.636 | 0 | 0 | 0 | 1 |
| LUAD-BS-14-G65174-TP-NT-SM-9J2YF-SM-9J2YG | Miao | Small Cell Lung Cancer | PD | 1.3 | Progressed | NCB | 1.234 | 1 | 0 | 0 | 0 |
| CU9061_T_N | Miao | Squamous | SD | 4.0 | Censored | NCB | 0 | 0 | 0 | 0 | 0 |
| GR4788_T_N | Miao | Squamous | PD | 1.9 | Progressed | NCB | 0 | 0 | 0 | 0 | 0 |
| LUAD-BS-10-J11656-TP-NT-SM-9J2Y7-SM-9J2Y8 | Miao | Squamous | SD | 4.3 | Progressed | NCB | 0 | 0 | 0 | 0 | 0 |
| LUAD-BS-11-R21845-TP-NT-SM-9J2YH-SM-9J2YI | Miao | Squamous | SD | 13.9 | Progressed | DCB | 0 | 0 | 0 | 0 | 0 |
| nsclc_mskcc_201810 | Hellmann | Squamous | SD | 5.4 | Progressed | NCB | 2.182 | 1 | 0 | 1 | 0 |
| nsclc_mskcc_20182 | Hellmann | Squamous | PR | 6.8 | Progressed | DCB | 0 | 0 | 0 | 0 | 0 |
| nsclc_mskcc_201830 | Hellmann | Squamous | PR | 21.2 | Censored | DCB | 0 | 0 | 0 | 0 | 0 |
| nsclc_mskcc_201833 | Hellmann | Squamous | PR | 21.6 | Censored | DCB | 1.234 | 1 | 0 | 0 | 0 |
| nsclc_mskcc_20184 | Hellmann | Squamous | SD | 3.8 | Progressed | NCB | 0 | 0 | 0 | 0 | 0 |
| nsclc_mskcc_201844 | Hellmann | Squamous | NA | 0.3 | Progressed | NCB | 0 | 0 | 0 | 0 | 0 |
| nsclc_mskcc_201845 | Hellmann | Squamous | NA | 2.2 | Progressed | NCB | 0 | 0 | 0 | 0 | 0 |
| nsclc_mskcc_201846 | Hellmann | Squamous | SD | 3.3 | Progressed | NCB | 3.276 | 0 | 1 | 0 | 1 |
| nsclc_mskcc_201847 | Hellmann | Squamous | PD | 2.1 | Progressed | NCB | 0 | 0 | 0 | 0 | 0 |
| nsclc_mskcc_201848 | Hellmann | Squamous | SD | 2.3 | Censored | NCB | 0 | 0 | 0 | 0 | 0 |
| nsclc_mskcc_201850 | Hellmann | Squamous | SD | 10.6 | Progressed | DCB | 0 | 0 | 0 | 0 | 0 |
| nsclc_mskcc_201863 | Hellmann | Squamous | PR | 21.9 | Censored | DCB | 0 | 0 | 0 | 0 | 0 |
| nsclc_mskcc_201866 | Hellmann | Squamous | NA | 0.0 | Censored | NCB | 0 | 0 | 0 | 0 | 0 |
| nsclc_mskcc_201867 | Hellmann | Squamous | PD | 2.5 | Progressed | NCB | 0 | 0 | 0 | 0 | 0 |
| nsclc_mskcc_20187 | Hellmann | Squamous | PR | 38.6 | Censored | DCB | 0 | 0 | 0 | 0 | 0 |
| nsclc_mskcc_201870 | Hellmann | Squamous | NA | 1.3 | Progressed | NCB | 0 | 0 | 0 | 0 | 0 |
| SB010944_T_N | Miao | Squamous | PR | 27.7 | Censored | DCB | 0 | 0 | 0 | 0 | 0 |
| SR070761_T_N | Miao | Squamous | PD | 3.4 | Progressed | NCB | 0 | 0 | 0 | 0 | 0 |
| SU2C_Lung-SU2C-DFCI-LUAD-1016-TM-NB-SM-AOL8W-SM-A46NS | Miao | Squamous | SD | 7.7 | Progressed | DCB | 1.64 | 0 | 1 | 0 | 0 |
