# Supplementary material for: Integrated genomic analysis identifies a genetic mutation model predicting response to immune checkpoint inhibitors in melanoma
Source: Cancer Med. 2020 Sep 24;9(22):8498–518. doi: 10.1002/cam4.3481 (PMC7666739; doi:10.1002/cam4.3481)
Supplement: Supplementary file 12 — Table S4 [file CAM4-9-8498-s012.docx]

| **Table S4. The mutational signatures of melanoma patients in the Allen cohort, Snyder cohort and Liu cohort** | | | | | | |
| --- | --- | --- | --- | --- | --- | --- |
|  | Percentage | | | Status | | |
| ID | Signature 7 | Signature 11 | Signature 1 | Signature 7 | Signature 11 | Signature 1 |
| Pat02 | 0.732 | 0.000 | 0.268 | 1 | 0 | 0 |
| Pat03 | 0.000 | 1.000 | 0.000 | 0 | 1 | 0 |
| Pat04 | 0.498 | 0.110 | 0.393 | 1 | 0 | 1 |
| Pat06 | 0.831 | 0.148 | 0.021 | 1 | 0 | 0 |
| Pat07 | 0.625 | 0.304 | 0.071 | 1 | 0 | 0 |
| Pat08 | 0.918 | 0.000 | 0.082 | 1 | 0 | 0 |
| Pat100 | 0.934 | 0.051 | 0.015 | 1 | 0 | 0 |
| Pat101 | 0.344 | 0.000 | 0.656 | 1 | 0 | 1 |
| Pat103 | 0.879 | 0.000 | 0.121 | 1 | 0 | 0 |
| Pat104 | 1.000 | 0.000 | 0.000 | 1 | 0 | 0 |
| Pat105 | 1.000 | 0.000 | 0.000 | 1 | 0 | 0 |
| Pat106 | 0.000 | 0.000 | 1.000 | 0 | 0 | 1 |
| Pat109 | 0.881 | 0.000 | 0.119 | 1 | 0 | 0 |
| Pat11 | 0.934 | 0.066 | 0.000 | 1 | 0 | 0 |
| Pat110 | 0.881 | 0.000 | 0.119 | 1 | 0 | 0 |
| Pat113 | 1.000 | 0.000 | 0.000 | 1 | 0 | 0 |
| Pat115 | 0.216 | 0.230 | 0.554 | 0 | 0 | 1 |
| Pat117 | 0.868 | 0.034 | 0.098 | 1 | 0 | 0 |
| Pat118 | 0.759 | 0.000 | 0.241 | 1 | 0 | 0 |
| Pat119 | 0.624 | 0.332 | 0.045 | 1 | 0 | 0 |
| Pat121 | 0.083 | 0.075 | 0.842 | 0 | 0 | 1 |
| Pat123 | 1.000 | 0.000 | 0.000 | 1 | 0 | 0 |
| Pat124 | 0.829 | 0.000 | 0.171 | 1 | 0 | 0 |
| Pat126 | 0.835 | 0.084 | 0.080 | 1 | 0 | 0 |
| Pat127 | 0.739 | 0.123 | 0.139 | 1 | 0 | 0 |
| Pat128 | 0.855 | 0.000 | 0.145 | 1 | 0 | 0 |
| Pat129 | 0.624 | 0.071 | 0.305 | 1 | 0 | 0 |
| Pat13 | 0.610 | 0.352 | 0.038 | 1 | 1 | 0 |
| Pat130 | 0.950 | 0.026 | 0.024 | 1 | 0 | 0 |
| Pat131 | 1.000 | 0.000 | 0.000 | 1 | 0 | 0 |
| Pat132 | 0.000 | 1.000 | 0.000 | 0 | 1 | 0 |
| Pat133 | 0.375 | 0.498 | 0.127 | 1 | 1 | 0 |
| Pat135 | 0.528 | 0.176 | 0.296 | 1 | 0 | 0 |
| Pat138 | 0.000 | 1.000 | 0.000 | 0 | 1 | 0 |
| Pat139 | 0.926 | 0.000 | 0.074 | 1 | 0 | 0 |
| Pat14 | 0.000 | 0.653 | 0.347 | 0 | 1 | 1 |
| Pat140 | 0.757 | 0.029 | 0.214 | 1 | 0 | 0 |
| Pat143 | 0.814 | 0.000 | 0.186 | 1 | 0 | 0 |
| Pat147 | 0.829 | 0.135 | 0.035 | 1 | 0 | 0 |
| Pat148 | 0.810 | 0.000 | 0.190 | 1 | 0 | 0 |
| Pat15 | 0.703 | 0.240 | 0.057 | 1 | 0 | 0 |
| Pat151 | 0.000 | 1.000 | 0.000 | 0 | 1 | 0 |
| Pat157 | 0.435 | 0.029 | 0.536 | 1 | 0 | 1 |
| Pat159 | 0.823 | 0.000 | 0.177 | 1 | 0 | 0 |
| Pat16 | 0.882 | 0.017 | 0.102 | 1 | 0 | 0 |
| Pat160 | 0.000 | 0.000 | 1.000 | 0 | 0 | 1 |
| Pat162 | 0.000 | 0.000 | 1.000 | 0 | 0 | 1 |
| Pat163 | 1.000 | 0.000 | 0.000 | 1 | 0 | 0 |
| Pat165 | 0.093 | 0.000 | 0.907 | 0 | 0 | 1 |
| Pat166 | 0.072 | 0.000 | 0.928 | 0 | 0 | 1 |
| Pat167 | 0.320 | 0.000 | 0.680 | 0 | 0 | 1 |
| Pat168 | 0.969 | 0.031 | 0.000 | 1 | 0 | 0 |
| Pat17 | 0.963 | 0.017 | 0.020 | 1 | 0 | 0 |
| Pat170 | 0.812 | 0.000 | 0.188 | 1 | 0 | 0 |
| Pat171 | 0.000 | 0.374 | 0.626 | 0 | 1 | 1 |
| Pat174 | 0.846 | 0.000 | 0.154 | 1 | 0 | 0 |
| Pat175 | 0.404 | 0.184 | 0.412 | 1 | 0 | 1 |
| Pat18 | 0.000 | 0.000 | 1.000 | 0 | 0 | 1 |
| Pat19 | 0.822 | 0.029 | 0.149 | 1 | 0 | 0 |
| Pat21 | 0.903 | 0.059 | 0.038 | 1 | 0 | 0 |
| Pat24 | 0.000 | 0.000 | 1.000 | 0 | 0 | 1 |
| Pat25 | 0.119 | 0.000 | 0.881 | 0 | 0 | 1 |
| Pat27 | 0.530 | 0.109 | 0.361 | 1 | 0 | 1 |
| Pat28 | 0.078 | 0.922 | 0.000 | 0 | 1 | 0 |
| Pat29 | 0.933 | 0.067 | 0.000 | 1 | 0 | 0 |
| Pat32 | 0.822 | 0.040 | 0.138 | 1 | 0 | 0 |
| Pat33 | 0.975 | 0.025 | 0.000 | 1 | 0 | 0 |
| Pat36 | 0.000 | 0.000 | 1.000 | 0 | 0 | 1 |
| Pat37 | 0.428 | 0.118 | 0.454 | 1 | 0 | 1 |
| Pat38 | 0.000 | 1.000 | 0.000 | 0 | 1 | 0 |
| Pat39 | 0.301 | 0.000 | 0.699 | 0 | 0 | 1 |
| Pat40 | 0.000 | 0.000 | 1.000 | 0 | 0 | 1 |
| Pat41 | 0.889 | 0.000 | 0.111 | 1 | 0 | 0 |
| Pat43 | 0.640 | 0.000 | 0.360 | 1 | 0 | 1 |
| Pat44 | 0.477 | 0.269 | 0.254 | 1 | 0 | 0 |
| Pat45 | 0.997 | 0.000 | 0.003 | 1 | 0 | 0 |
| Pat46 | 0.901 | 0.099 | 0.000 | 1 | 0 | 0 |
| Pat47 | 0.575 | 0.179 | 0.246 | 1 | 0 | 0 |
| Pat49 | 0.806 | 0.055 | 0.139 | 1 | 0 | 0 |
| Pat50 | 0.899 | 0.095 | 0.006 | 1 | 0 | 0 |
| Pat54 | 0.949 | 0.044 | 0.007 | 1 | 0 | 0 |
| Pat55 | 0.639 | 0.277 | 0.085 | 1 | 0 | 0 |
| Pat56 | 0.103 | 0.000 | 0.897 | 0 | 0 | 1 |
| Pat57 | 0.929 | 0.000 | 0.071 | 1 | 0 | 0 |
| Pat58 | 0.039 | 0.961 | 0.000 | 0 | 1 | 0 |
| Pat59 | 0.928 | 0.072 | 0.000 | 1 | 0 | 0 |
| Pat60 | 0.919 | 0.077 | 0.004 | 1 | 0 | 0 |
| Pat62 | 0.620 | 0.287 | 0.093 | 1 | 0 | 0 |
| Pat63 | 0.944 | 0.051 | 0.005 | 1 | 0 | 0 |
| Pat64 | 0.766 | 0.000 | 0.234 | 1 | 0 | 0 |
| Pat66 | 0.890 | 0.019 | 0.091 | 1 | 0 | 0 |
| Pat67 | 0.482 | 0.000 | 0.518 | 1 | 0 | 1 |
| Pat70 | 1.000 | 0.000 | 0.000 | 1 | 0 | 0 |
| Pat71 | 0.890 | 0.000 | 0.110 | 1 | 0 | 0 |
| Pat73 | 0.763 | 0.143 | 0.093 | 1 | 0 | 0 |
| Pat74 | 0.752 | 0.113 | 0.135 | 1 | 0 | 0 |
| Pat76 | 0.897 | 0.041 | 0.062 | 1 | 0 | 0 |
| Pat77 | 0.933 | 0.047 | 0.020 | 1 | 0 | 0 |
| Pat78 | 0.000 | 0.000 | 0.000 | 0 | 0 | 0 |
| Pat79 | 1.000 | 0.000 | 0.000 | 1 | 0 | 0 |
| Pat80 | 0.000 | 1.000 | 0.000 | 0 | 1 | 0 |
| Pat81 | 0.000 | 0.000 | 1.000 | 0 | 0 | 1 |
| Pat82 | 0.632 | 0.074 | 0.294 | 1 | 0 | 0 |
| Pat83 | 0.499 | 0.000 | 0.501 | 1 | 0 | 1 |
| Pat85 | 0.762 | 0.000 | 0.238 | 1 | 0 | 0 |
| Pat86 | 0.903 | 0.000 | 0.097 | 1 | 0 | 0 |
| Pat88 | 0.956 | 0.017 | 0.027 | 1 | 0 | 0 |
| Pat90 | 0.909 | 0.000 | 0.091 | 1 | 0 | 0 |
| Pat92 | 0.091 | 0.000 | 0.909 | 0 | 0 | 1 |
| Pat98 | 0.053 | 0.000 | 0.947 | 0 | 0 | 1 |
| CR04885 | 0.849 | 0.035 | 0.115 | 1 | 0 | 0 |
| CR06670 | 0.834 | 0.035 | 0.131 | 1 | 0 | 0 |
| CR22640 | 0.851 | 0.000 | 0.149 | 1 | 0 | 0 |
| CR6161 | 0.961 | 0.039 | 0.000 | 1 | 0 | 0 |
| CRNR4941 | 0.566 | 0.434 | 0.000 | 1 | 1 | 0 |
| LSD4691 | 0.157 | 0.825 | 0.018 | 0 | 1 | 0 |
| LSDNR3086 | 0.727 | 0.114 | 0.159 | 1 | 0 | 0 |
| NR4018 | 0.825 | 0.000 | 0.175 | 1 | 0 | 0 |
| NR4083 | 0.670 | 0.000 | 0.330 | 1 | 0 | 0 |
| NR9341 | 0.754 | 0.147 | 0.099 | 1 | 0 | 0 |
| PR03803 | 0.617 | 0.000 | 0.383 | 1 | 0 | 1 |
| PR12117 | 0.850 | 0.049 | 0.101 | 1 | 0 | 0 |
| PR4035 | 0.861 | 0.000 | 0.139 | 1 | 0 | 0 |
| PR4046 | 0.633 | 0.122 | 0.245 | 1 | 0 | 0 |
| PR4077 | 0.931 | 0.000 | 0.069 | 1 | 0 | 0 |
| PR4091 | 0.831 | 0.104 | 0.065 | 1 | 0 | 0 |
| PR4092 | 0.002 | 0.998 | 0.000 | 0 | 1 | 0 |
| SD6336 | 0.716 | 0.173 | 0.111 | 1 | 0 | 0 |
| CR4880 | 0.108 | 0.053 | 0.839 | 0 | 0 | 1 |
| CR0095 | 0.705 | 0.172 | 0.123 | 1 | 0 | 0 |
| CR9306 | 0.747 | 0.147 | 0.106 | 1 | 0 | 0 |
| CRNR2472 | 0.908 | 0.068 | 0.024 | 1 | 0 | 0 |
| SD5038 | 0.965 | 0.000 | 0.035 | 1 | 0 | 0 |
| NR6721 | 0.682 | 0.000 | 0.318 | 1 | 0 | 0 |
| LSD0167 | 0.855 | 0.000 | 0.145 | 1 | 0 | 0 |
| NR4949 | 0.678 | 0.068 | 0.254 | 1 | 0 | 0 |
| NR9705 | 0.000 | 0.000 | 1.000 | 0 | 0 | 1 |
| NR9521 | 0.959 | 0.041 | 0.000 | 1 | 0 | 0 |
| NR6842 | 0.000 | 0.000 | 1.000 | 0 | 0 | 1 |
| LSD6819 | 0.975 | 0.000 | 0.025 | 1 | 0 | 0 |
| LSDNR1120 | 0.952 | 0.035 | 0.012 | 1 | 0 | 0 |
| SD5934 | 0.802 | 0.063 | 0.135 | 1 | 0 | 0 |
| CRNR0244 | 0.917 | 0.068 | 0.015 | 1 | 0 | 0 |
| NR8815 | 0.925 | 0.000 | 0.075 | 1 | 0 | 0 |
| CR1509 | 0.802 | 0.124 | 0.074 | 1 | 0 | 0 |
| SD5118 | 0.000 | 0.000 | 1.000 | 0 | 0 | 1 |
| NR9765 | 0.425 | 0.289 | 0.286 | 1 | 0 | 0 |
| CR9699 | 0.997 | 0.000 | 0.003 | 1 | 0 | 0 |
| SD2056 | 0.804 | 0.100 | 0.096 | 1 | 0 | 0 |
| NR8727 | 0.000 | 0.000 | 0.000 | 0 | 0 | 0 |
| NR9449 | 0.992 | 0.000 | 0.008 | 1 | 0 | 0 |
| NR3156 | 0.000 | 0.000 | 1.000 | 0 | 0 | 1 |
| NR6689 | 0.000 | 0.000 | 1.000 | 0 | 0 | 1 |
| SD0346 | 0.767 | 0.096 | 0.136 | 1 | 0 | 0 |
| CR6126 | 0.829 | 0.061 | 0.110 | 1 | 0 | 0 |
| NR1867 | 0.034 | 0.000 | 0.966 | 0 | 0 | 1 |
| LSDNR9298 | 0.822 | 0.000 | 0.178 | 1 | 0 | 0 |
| SD7357 | 0.964 | 0.000 | 0.036 | 1 | 0 | 0 |
| NR4045 | 0.964 | 0.036 | 0.000 | 1 | 0 | 0 |
| CR3665 | 0.476 | 0.428 | 0.096 | 1 | 1 | 0 |
| LSD4744 | 0.809 | 0.020 | 0.171 | 1 | 0 | 0 |
| NR5784 | 0.965 | 0.000 | 0.035 | 1 | 0 | 0 |
| LSDNR1650 | 0.000 | 0.000 | 0.000 | 0 | 0 | 0 |
| NR2137 | 0.850 | 0.095 | 0.055 | 1 | 0 | 0 |
| NR3549 | 0.730 | 0.073 | 0.198 | 1 | 0 | 0 |
| SD6494 | 0.830 | 0.030 | 0.139 | 1 | 0 | 0 |
| SD1494 | 0.864 | 0.036 | 0.100 | 1 | 0 | 0 |
| LSD2057 | 0.867 | 0.041 | 0.091 | 1 | 0 | 0 |
| NR4810 | 0.856 | 0.053 | 0.091 | 1 | 0 | 0 |
| NR4631 | 0.921 | 0.000 | 0.079 | 1 | 0 | 0 |
| CR7623 | 0.895 | 0.105 | 0.000 | 1 | 0 | 0 |
| SD2051 | 0.000 | 0.392 | 0.608 | 0 | 1 | 1 |
| LSD3484 | 0.804 | 0.085 | 0.111 | 1 | 0 | 0 |
| NR9445 | 0.894 | 0.106 | 0.000 | 1 | 0 | 0 |
| Patient40 | 0.112 | 0.888 | 0.000 | 0 | 1 | 0 |
| Patient49 | 0.050 | 0.950 | 0.000 | 0 | 1 | 0 |
| Patient206 | 0.991 | 0.000 | 0.009 | 1 | 0 | 0 |
| Patient41 | 0.985 | 0.000 | 0.015 | 1 | 0 | 0 |
| Patient158 | 0.076 | 0.908 | 0.016 | 0 | 1 | 0 |
| Patient6 | 0.062 | 0.921 | 0.017 | 0 | 1 | 0 |
| Patient191 | 0.972 | 0.005 | 0.023 | 1 | 0 | 0 |
| Patient62 | 0.962 | 0.000 | 0.038 | 1 | 0 | 0 |
| Patient196 | 0.927 | 0.032 | 0.041 | 1 | 0 | 0 |
| Patient61 | 0.946 | 0.012 | 0.042 | 1 | 0 | 0 |
| Patient169 | 0.909 | 0.039 | 0.052 | 1 | 0 | 0 |
| Patient162 | 0.942 | 0.000 | 0.058 | 1 | 0 | 0 |
| Patient193 | 0.929 | 0.012 | 0.059 | 1 | 0 | 0 |
| Patient82 | 0.906 | 0.035 | 0.059 | 1 | 0 | 0 |
| Patient4 | 0.938 | 0.000 | 0.062 | 1 | 0 | 0 |
| Patient96 | 0.931 | 0.003 | 0.067 | 1 | 0 | 0 |
| Patient25 | 0.838 | 0.094 | 0.068 | 1 | 0 | 0 |
| Patient132 | 0.886 | 0.046 | 0.069 | 1 | 0 | 0 |
| Patient46 | 0.873 | 0.058 | 0.069 | 1 | 0 | 0 |
| Patient120 | 0.907 | 0.023 | 0.070 | 1 | 0 | 0 |
| Patient51 | 0.903 | 0.027 | 0.070 | 1 | 0 | 0 |
| Patient47 | 0.913 | 0.012 | 0.075 | 1 | 0 | 0 |
| Patient137 | 0.923 | 0.000 | 0.077 | 1 | 0 | 0 |
| Patient133 | 0.834 | 0.087 | 0.078 | 1 | 0 | 0 |
| Patient205 | 0.897 | 0.025 | 0.078 | 1 | 0 | 0 |
| Patient180 | 0.877 | 0.045 | 0.079 | 1 | 0 | 0 |
| Patient200 | 0.579 | 0.341 | 0.081 | 1 | 1 | 0 |
| Patient32 | 0.879 | 0.040 | 0.082 | 1 | 0 | 0 |
| Patient192 | 0.905 | 0.012 | 0.083 | 1 | 0 | 0 |
| Patient184 | 0.912 | 0.002 | 0.086 | 1 | 0 | 0 |
| Patient144 | 0.910 | 0.003 | 0.086 | 1 | 0 | 0 |
| Patient75 | 0.889 | 0.024 | 0.087 | 1 | 0 | 0 |
| Patient110 | 0.902 | 0.011 | 0.087 | 1 | 0 | 0 |
| Patient146 | 0.847 | 0.065 | 0.089 | 1 | 0 | 0 |
| Patient165 | 0.892 | 0.011 | 0.097 | 1 | 0 | 0 |
| Patient135 | 0.855 | 0.048 | 0.097 | 1 | 0 | 0 |
| Patient157 | 0.824 | 0.079 | 0.098 | 1 | 0 | 0 |
| Patient20 | 0.780 | 0.122 | 0.098 | 1 | 0 | 0 |
| Patient179 | 0.866 | 0.033 | 0.101 | 1 | 0 | 0 |
| Patient195 | 0.893 | 0.006 | 0.101 | 1 | 0 | 0 |
| Patient145 | 0.888 | 0.010 | 0.101 | 1 | 0 | 0 |
| Patient134 | 0.814 | 0.082 | 0.104 | 1 | 0 | 0 |
| Patient140 | 0.885 | 0.011 | 0.105 | 1 | 0 | 0 |
| Patient163 | 0.850 | 0.045 | 0.106 | 1 | 0 | 0 |
| Patient99 | 0.856 | 0.038 | 0.106 | 1 | 0 | 0 |
| Patient98 | 0.878 | 0.015 | 0.107 | 1 | 0 | 0 |
| Patient127 | 0.785 | 0.107 | 0.108 | 1 | 0 | 0 |
| Patient155 | 0.842 | 0.049 | 0.109 | 1 | 0 | 0 |
| Patient13 | 0.864 | 0.026 | 0.110 | 1 | 0 | 0 |
| Patient121 | 0.890 | 0.000 | 0.110 | 1 | 0 | 0 |
| Patient172 | 0.859 | 0.030 | 0.111 | 1 | 0 | 0 |
| Patient147 | 0.888 | 0.000 | 0.111 | 1 | 0 | 0 |
| Patient55 | 0.876 | 0.013 | 0.112 | 1 | 0 | 0 |
| Patient204 | 0.850 | 0.036 | 0.114 | 1 | 0 | 0 |
| Patient60 | 0.885 | 0.000 | 0.115 | 1 | 0 | 0 |
| Patient116 | 0.879 | 0.005 | 0.115 | 1 | 0 | 0 |
| Patient59 | 0.831 | 0.053 | 0.116 | 1 | 0 | 0 |
| Patient126 | 0.867 | 0.014 | 0.119 | 1 | 0 | 0 |
| Patient77 | 0.809 | 0.071 | 0.119 | 1 | 0 | 0 |
| Patient136 | 0.839 | 0.041 | 0.120 | 1 | 0 | 0 |
| Patient106 | 0.802 | 0.077 | 0.121 | 1 | 0 | 0 |
| Patient102 | 0.809 | 0.069 | 0.122 | 1 | 0 | 0 |
| Patient148 | 0.827 | 0.049 | 0.124 | 1 | 0 | 0 |
| Patient149 | 0.868 | 0.006 | 0.126 | 1 | 0 | 0 |
| Patient86 | 0.812 | 0.061 | 0.127 | 1 | 0 | 0 |
| Patient94 | 0.871 | 0.000 | 0.129 | 1 | 0 | 0 |
| Patient112 | 0.823 | 0.048 | 0.129 | 1 | 0 | 0 |
| Patient183 | 0.822 | 0.045 | 0.134 | 1 | 0 | 0 |
| Patient18 | 0.815 | 0.050 | 0.136 | 1 | 0 | 0 |
| Patient185 | 0.807 | 0.056 | 0.136 | 1 | 0 | 0 |
| Patient131 | 0.847 | 0.016 | 0.137 | 1 | 0 | 0 |
| Patient182 | 0.806 | 0.055 | 0.139 | 1 | 0 | 0 |
| Patient156 | 0.802 | 0.058 | 0.140 | 1 | 0 | 0 |
| Patient42 | 0.825 | 0.029 | 0.145 | 1 | 0 | 0 |
| Patient130 | 0.852 | 0.002 | 0.146 | 1 | 0 | 0 |
| Patient83 | 0.788 | 0.064 | 0.148 | 1 | 0 | 0 |
| Patient181 | 0.822 | 0.030 | 0.148 | 1 | 0 | 0 |
| Patient45 | 0.836 | 0.015 | 0.149 | 1 | 0 | 0 |
| Patient27 | 0.746 | 0.105 | 0.149 | 1 | 0 | 0 |
| Patient36 | 0.800 | 0.046 | 0.154 | 1 | 0 | 0 |
| Patient79 | 0.716 | 0.121 | 0.163 | 1 | 0 | 0 |
| Patient50 | 0.821 | 0.015 | 0.164 | 1 | 0 | 0 |
| Patient8 | 0.812 | 0.021 | 0.167 | 1 | 0 | 0 |
| Patient24 | 0.757 | 0.074 | 0.170 | 1 | 0 | 0 |
| Patient187 | 0.767 | 0.062 | 0.172 | 1 | 0 | 0 |
| Patient154 | 0.680 | 0.145 | 0.176 | 1 | 0 | 0 |
| Patient118 | 0.785 | 0.038 | 0.177 | 1 | 0 | 0 |
| Patient9 | 0.777 | 0.044 | 0.178 | 1 | 0 | 0 |
| Patient100 | 0.745 | 0.070 | 0.185 | 1 | 0 | 0 |
| Patient143 | 0.753 | 0.052 | 0.195 | 1 | 0 | 0 |
| Patient17 | 0.758 | 0.042 | 0.200 | 1 | 0 | 0 |
| Patient58 | 0.658 | 0.126 | 0.216 | 1 | 0 | 0 |
| Patient72 | 0.599 | 0.158 | 0.243 | 1 | 0 | 0 |
| Patient30 | 0.746 | 0.010 | 0.244 | 1 | 0 | 0 |
| Patient23 | 0.615 | 0.107 | 0.277 | 1 | 0 | 0 |
| Patient107 | 0.619 | 0.101 | 0.279 | 1 | 0 | 0 |
| Patient15 | 0.612 | 0.085 | 0.304 | 1 | 0 | 0 |
| Patient168 | 0.642 | 0.044 | 0.314 | 1 | 0 | 0 |
| Patient105 | 0.575 | 0.106 | 0.319 | 1 | 0 | 0 |
| Patient141 | 0.615 | 0.052 | 0.334 | 1 | 0 | 1 |
| Patient35 | 0.664 | 0.000 | 0.336 | 1 | 0 | 1 |
| Patient54 | 0.481 | 0.167 | 0.352 | 1 | 0 | 1 |
| Patient11 | 0.517 | 0.075 | 0.409 | 1 | 0 | 1 |
| Patient125 | 0.448 | 0.122 | 0.430 | 1 | 0 | 1 |
| Patient10 | 0.422 | 0.080 | 0.498 | 1 | 0 | 1 |
| Patient197 | 0.355 | 0.132 | 0.513 | 1 | 0 | 1 |
| Patient142 | 0.450 | 0.036 | 0.514 | 1 | 0 | 1 |
| Patient14 | 0.270 | 0.214 | 0.516 | 0 | 0 | 1 |
| Patient31 | 0.333 | 0.133 | 0.534 | 0 | 0 | 1 |
| Patient73 | 0.390 | 0.054 | 0.556 | 1 | 0 | 1 |
| Patient38 | 0.434 | 0.003 | 0.563 | 1 | 0 | 1 |
| Patient119 | 0.370 | 0.037 | 0.593 | 1 | 0 | 1 |
| Patient87 | 0.282 | 0.089 | 0.630 | 0 | 0 | 1 |
| Patient173 | 0.229 | 0.140 | 0.632 | 0 | 0 | 1 |
| Patient159 | 0.076 | 0.258 | 0.667 | 0 | 0 | 1 |
| Patient80 | 0.240 | 0.078 | 0.682 | 0 | 0 | 1 |
| Patient170 | 0.190 | 0.126 | 0.684 | 0 | 0 | 1 |
| Patient108 | 0.204 | 0.091 | 0.706 | 0 | 0 | 1 |
| Patient203 | 0.249 | 0.037 | 0.714 | 0 | 0 | 1 |
| Patient43 | 0.173 | 0.112 | 0.715 | 0 | 0 | 1 |
| Patient22 | 0.225 | 0.054 | 0.721 | 0 | 0 | 1 |
| Patient48 | 0.243 | 0.027 | 0.730 | 0 | 0 | 1 |
| Patient117 | 0.266 | 0.000 | 0.734 | 0 | 0 | 1 |
| Patient78 | 0.154 | 0.092 | 0.754 | 0 | 0 | 1 |
| Patient7 | 0.136 | 0.082 | 0.782 | 0 | 0 | 1 |
| Patient56 | 0.000 | 0.199 | 0.801 | 0 | 0 | 1 |
| Patient88 | 0.149 | 0.042 | 0.809 | 0 | 0 | 1 |
| Patient67 | 0.130 | 0.060 | 0.810 | 0 | 0 | 1 |
| Patient34 | 0.000 | 0.187 | 0.813 | 0 | 0 | 1 |
| Patient104 | 0.146 | 0.034 | 0.820 | 0 | 0 | 1 |
| Patient33 | 0.000 | 0.170 | 0.830 | 0 | 0 | 1 |
| Patient188 | 0.096 | 0.072 | 0.832 | 0 | 0 | 1 |
| Patient189 | 0.150 | 0.018 | 0.832 | 0 | 0 | 1 |
| Patient201 | 0.071 | 0.097 | 0.833 | 0 | 0 | 1 |
| Patient44 | 0.047 | 0.102 | 0.851 | 0 | 0 | 1 |
| Patient39 | 0.147 | 0.000 | 0.853 | 0 | 0 | 1 |
| Patient63 | 0.000 | 0.129 | 0.871 | 0 | 0 | 1 |
| Patient84 | 0.003 | 0.119 | 0.878 | 0 | 0 | 1 |
| Patient150 | 0.106 | 0.000 | 0.894 | 0 | 0 | 1 |
| Patient1 | 0.016 | 0.079 | 0.905 | 0 | 0 | 1 |
| Patient167 | 0.000 | 0.084 | 0.916 | 0 | 0 | 1 |
| Patient21 | 0.000 | 0.077 | 0.923 | 0 | 0 | 1 |
| Patient166 | 0.000 | 0.076 | 0.924 | 0 | 0 | 1 |
| Patient37 | 0.076 | 0.000 | 0.924 | 0 | 0 | 1 |
