# Supplementary material for: Integrated genomic analysis identifies a genetic mutation model predicting response to immune checkpoint inhibitors in melanoma
Source: Cancer Med. 2020 Sep 24;9(22):8498–518. doi: 10.1002/cam4.3481 (PMC7666739; doi:10.1002/cam4.3481)
Supplement: Supplementary file 13 — Table S5 [file CAM4-9-8498-s013.docx]

| **Table S5. The association between frequently mutated genes and response to ICIs therapy** | | | | | | | | |
| --- | --- | --- | --- | --- | --- | --- | --- | --- |
| Gene | Mutation Rate (%) | *P* | Gene | Mutation Rate (%) | *P* | Gene | Mutation Rate (%) | *P* |
| *COL4A5* | 20.91 | **0.00** | *STAB2* | 18.18 | 0.08 | *SPEG* | 10.91 | 0.16 |
| *NOTCH4* | 12.73 | **0.01** | *TNXB* | 18.18 | 0.08 | *ITPR1* | 10.91 | 0.16 |
| *THSD7B* | 21.82 | **0.01** | *ZFHX4* | 18.18 | 0.08 | *EGFLAM* | 10.91 | 0.16 |
| *SCN1A* | 10.91 | **0.01** | *CNTNAP2* | 17.27 | 0.08 | *MAST4* | 10.91 | 0.16 |
| *ABCA4* | 10.91 | **0.01** | *SI* | 17.27 | 0.08 | *DSG3* | 10.91 | 0.16 |
| *SLITRK3* | 10.91 | **0.01** | *LAMB4* | 17.27 | 0.08 | *SNCAIP* | 10.91 | 0.16 |
| *SYNE2* | 10.91 | **0.01** | *DCHS2* | 11.82 | 0.08 | *GON4L* | 10.91 | 0.16 |
| *FAM83B* | 22.73 | **0.01** | *ARID2* | 11.82 | 0.08 | *LYST* | 10.91 | 0.16 |
| *GRM3* | 20.00 | **0.01** | *SZT2* | 11.82 | 0.08 | *SPTB* | 10.91 | 0.16 |
| *EPHA7* | 16.36 | **0.01** | *DPYD* | 11.82 | 0.08 | *KDR* | 10.91 | 0.16 |
| *HYDIN* | 26.36 | **0.01** | *ATRNL1* | 11.82 | 0.08 | *NYAP2* | 10.91 | 0.16 |
| *NLRP10* | 11.82 | **0.02** | *DLEC1* | 11.82 | 0.08 | *FLNB* | 10.91 | 0.16 |
| *ZNF804A* | 20.91 | **0.02** | *PKHD1L1* | 28.18 | 0.10 | *LRRN1* | 10.91 | 0.16 |
| *COL4A4* | 14.55 | **0.02** | *LRP2* | 21.82 | 0.10 | *FRAS1* | 11.82 | 0.18 |
| *PCDH15* | 24.55 | **0.02** | *FAT3* | 22.73 | 0.10 | *HERC2* | 13.64 | 0.19 |
| *FLNC* | 10.00 | **0.03** | *LAMA1* | 12.73 | 0.10 | *KCNT2* | 13.64 | 0.19 |
| *MYO9A* | 10.00 | **0.03** | *COL1A2* | 12.73 | 0.10 | *PLIN4* | 13.64 | 0.19 |
| *COL11A2* | 10.00 | **0.03** | *ANKS1B* | 12.73 | 0.10 | *C6* | 13.64 | 0.19 |
| *PRUNE2* | 19.09 | **0.03** | *CNTN4* | 12.73 | 0.10 | *PTPRD* | 20.91 | 0.20 |
| *ANKRD30A* | 15.45 | **0.03** | *MCTP2* | 12.73 | 0.10 | *RYR1* | 20.91 | 0.20 |
| *NRXN1* | 15.45 | **0.03** | *MUC16* | 60.91 | 0.11 | *RELN* | 20.91 | 0.20 |
| *XIRP2* | 28.18 | **0.03** | *DCC* | 15.45 | 0.12 | *ANK3* | 30.91 | 0.20 |
| *ASPM* | 12.73 | **0.04** | *NOS1* | 15.45 | 0.12 | *SORL1* | 14.55 | 0.22 |
| *SCN2A* | 12.73 | **0.04** | *PLA2R1* | 15.45 | 0.12 | *TENM2* | 14.55 | 0.22 |
| *MYO5B* | 12.73 | **0.04** | *NEB* | 22.73 | 0.13 | *COL9A1* | 14.55 | 0.22 |
| *SCN5A* | 13.64 | 0.05 | *MXRA5* | 22.73 | 0.13 | *COL4A3* | 14.55 | 0.22 |
| *CDH9* | 13.64 | 0.05 | *VPS13A* | 10.00 | 0.13 | *MPP7* | 14.55 | 0.22 |
| *NEBL* | 13.64 | 0.05 | *MYOM2* | 10.00 | 0.13 | *FAT2* | 14.55 | 0.22 |
| *KMT2C* | 14.55 | 0.07 | *MAGEC3* | 10.00 | 0.13 | *CFH* | 14.55 | 0.22 |
| *LAMA3* | 14.55 | 0.07 | *NRG1* | 10.00 | 0.13 | *TNC* | 14.55 | 0.22 |
| *DMBT1* | 14.55 | 0.07 | *SLC8A3* | 10.00 | 0.13 | *PPFIA2* | 14.55 | 0.22 |
| *COL5A1* | 14.55 | 0.07 | *NF1* | 16.36 | 0.14 | *COL5A3* | 14.55 | 0.22 |
| *KALRN* | 14.55 | 0.07 | *AHNAK2* | 16.36 | 0.14 | *PREX2* | 24.55 | 0.22 |
| *RIMS1* | 10.91 | 0.07 | *TEX15* | 16.36 | 0.14 | *CACNA1E* | 24.55 | 0.22 |
| *MYH11* | 10.91 | 0.07 | *TRANK1* | 20.91 | 0.15 | *MYO18B* | 19.09 | 0.23 |
| *FRG1* | 10.91 | 0.07 | *DNAH9* | 20.00 | 0.15 | *COL3A1* | 18.18 | 0.23 |
| *PDZD2* | 10.91 | 0.07 | *MYH1* | 20.00 | 0.15 | *NBEA* | 18.18 | 0.23 |
| *COL6A6* | 10.91 | 0.07 | *LRP1B* | 30.00 | 0.16 | *UNC79* | 18.18 | 0.23 |
| *PDE4DIP* | 10.91 | 0.07 | *NAV3* | 10.91 | 0.16 | *GRIN2A* | 18.18 | 0.23 |
| *DCLK3* | 10.91 | 0.07 | *KMT2A* | 10.91 | 0.16 | *FREM2* | 15.45 | 0.23 |
| *ZNF99* | 17.27 | 0.24 | *PTPRB* | 15.45 | 0.36 | *BNC1* | 10.91 | 0.48 |
| *MECOM* | 17.27 | 0.24 | *CASR* | 15.45 | 0.36 | *SLFN11* | 10.91 | 0.48 |
| *GPR158* | 10.91 | 0.29 | *TACC2* | 16.36 | 0.38 | *MACC1* | 10.91 | 0.48 |
| *ZNF285* | 10.91 | 0.29 | *PAPPA2* | 16.36 | 0.38 | *TC2N* | 10.91 | 0.48 |
| *CACNA1S* | 10.00 | 0.29 | *CMYA5* | 20.00 | 0.38 | *APC* | 10.91 | 0.48 |
| *TIAM2* | 10.00 | 0.29 | *ABCA13* | 20.00 | 0.38 | *RANBP2* | 10.91 | 0.48 |
| *RGPD4* | 10.00 | 0.29 | *SCN10A* | 23.64 | 0.40 | *C3* | 10.91 | 0.48 |
| *HEPH* | 10.00 | 0.29 | *TTN* | 63.64 | 0.40 | *SAMD9L* | 10.91 | 0.48 |
| *CCDC141* | 10.00 | 0.29 | *RYR2* | 17.27 | 0.43 | *MAGI2* | 10.91 | 0.48 |
| *SLC34A2* | 10.00 | 0.29 | *ADAMTS20* | 17.27 | 0.43 | *PCDHA3* | 10.91 | 0.48 |
| *PLCL2* | 10.00 | 0.29 | *USH2A* | 34.55 | 0.44 | *LTBP1* | 10.91 | 0.48 |
| *ACSM1* | 10.00 | 0.29 | *HRNR* | 20.00 | 0.44 | *KSR2* | 10.91 | 0.48 |
| *MAGEC1* | 26.36 | 0.29 | *DNAH5* | 38.18 | 0.44 | *PTPN11* | 10.91 | 0.48 |
| *TENM1* | 19.09 | 0.30 | *NRAS* | 31.82 | 0.45 | *ITGA11* | 10.91 | 0.48 |
| *VPS13B* | 11.82 | 0.30 | *TRRAP* | 10.00 | 0.46 | *DSC3* | 10.91 | 0.48 |
| *UBR4* | 11.82 | 0.30 | *PLXNA2* | 10.00 | 0.46 | *GRID2* | 12.73 | 0.51 |
| *ALPK2* | 11.82 | 0.30 | *MYH14* | 10.00 | 0.46 | *MYOCD* | 12.73 | 0.51 |
| *ABCC9* | 11.82 | 0.30 | *ST18* | 10.00 | 0.46 | *EXPH5* | 12.73 | 0.51 |
| *ATR* | 11.82 | 0.30 | *SPATA31D1* | 10.00 | 0.46 | *PSD4* | 12.73 | 0.51 |
| *CACNA2D3* | 11.82 | 0.30 | *MYH6* | 10.00 | 0.46 | *CDH23* | 12.73 | 0.51 |
| *CNGB1* | 11.82 | 0.30 | *FREM1* | 10.00 | 0.46 | *CHD7* | 11.82 | 0.51 |
| *GABRG1* | 11.82 | 0.30 | *TRERF1* | 10.00 | 0.46 | *NPAP1* | 11.82 | 0.51 |
| *DSG4* | 11.82 | 0.30 | *PCDHB4* | 10.00 | 0.46 | *SPAG17* | 11.82 | 0.51 |
| *CCDC68* | 11.82 | 0.30 | *ITGAX* | 10.00 | 0.46 | *SCN7A* | 11.82 | 0.51 |
| *SYNE1* | 22.73 | 0.32 | *PLB1* | 10.00 | 0.46 | *TMEM132B* | 11.82 | 0.51 |
| *FAT4* | 22.73 | 0.32 | *HNRNPCL1* | 10.00 | 0.46 | *TSHZ2* | 11.82 | 0.51 |
| *MGAM* | 22.73 | 0.32 | *TSC1* | 10.00 | 0.46 | *ZFPM2* | 19.09 | 0.52 |
| *VPS13D* | 12.73 | 0.33 | *RP1L1* | 20.00 | 0.47 | *DMD* | 13.64 | 0.52 |
| *IGSF10* | 12.73 | 0.33 | *ZAN* | 23.64 | 0.47 | *MYO15A* | 13.64 | 0.52 |
| *STK31* | 12.73 | 0.33 | *MUC17* | 24.55 | 0.48 | *BRINP3* | 13.64 | 0.52 |
| *ADCY8* | 12.73 | 0.33 | *KMT2B* | 10.91 | 0.48 | *COL4A6* | 13.64 | 0.52 |
| *FBN2* | 12.73 | 0.33 | *ATM* | 10.91 | 0.48 | *MYCBP2* | 13.64 | 0.52 |
| *SCN4A* | 12.73 | 0.33 | *PXDNL* | 10.91 | 0.48 | *DYNC1H1* | 13.64 | 0.52 |
| *DPP10* | 12.73 | 0.33 | *AFF2* | 10.91 | 0.48 | *PLCB1* | 13.64 | 0.52 |
| *CDH6* | 12.73 | 0.33 | *GPR179* | 10.91 | 0.48 | *NLRP8* | 13.64 | 0.52 |
| *ERC2* | 12.73 | 0.33 | *LHCGR* | 10.91 | 0.48 | *ANO4* | 13.64 | 0.52 |
| *APOB* | 25.45 | 0.34 | *BAZ2B* | 10.91 | 0.48 | *DSG1* | 13.64 | 0.52 |
| *BIRC6* | 14.55 | 0.35 | *RNF17* | 10.91 | 0.48 | *FLG2* | 18.18 | 0.53 |
| *PAPPA* | 14.55 | 0.35 | *CCT8L2* | 10.91 | 0.48 | *CDC27* | 18.18 | 0.53 |
| *TP53* | 13.64 | 0.35 | *SLC8A1* | 10.91 | 0.48 | *RIMS2* | 14.55 | 0.54 |
| *SCN11A* | 13.64 | 0.35 | *MRC2* | 10.91 | 0.48 | *SPEF2* | 14.55 | 0.54 |
| *SRRM2* | 15.45 | 0.36 | *MMRN1* | 10.91 | 0.48 | *RPTN* | 14.55 | 0.54 |
| *CD163* | 15.45 | 0.36 | *CFTR* | 10.91 | 0.48 | *KIAA1109* | 14.55 | 0.54 |
| *COL6A3* | 14.55 | 0.54 | *SLC9A2* | 11.82 | 0.73 | *SCN9A* | 21.82 | 0.95 |
| *ACAN* | 14.55 | 0.54 | *GALNT13* | 11.82 | 0.73 | *FER1L6* | 18.18 | 0.96 |
| *BCLAF1* | 16.36 | 0.55 | *MUC5B* | 12.73 | 0.74 | *SVEP1* | 17.27 | 1.00 |
| *CD163L1* | 16.36 | 0.55 | *PKHD1* | 12.73 | 0.74 | *COL21A1* | 17.27 | 1.00 |
| *NLRP4* | 15.45 | 0.56 | *VWF* | 12.73 | 0.74 | *ASTN1* | 17.27 | 1.00 |
| *MYH2* | 17.27 | 0.56 | *MKI67* | 12.73 | 0.74 | *PIK3C2G* | 17.27 | 1.00 |
| *ASXL3* | 17.27 | 0.56 | *ADCY10* | 12.73 | 0.74 | *COL7A1* | 17.27 | 1.00 |
| *OBSCN* | 18.18 | 0.60 | *ANO2* | 12.73 | 0.74 | *ANK2* | 16.36 | 1.00 |
| *DNAH17* | 18.18 | 0.60 | *ADAMTS6* | 12.73 | 0.74 | *BRINP2* | 16.36 | 1.00 |
| *MROH2B* | 18.18 | 0.60 | *BRCA2* | 12.73 | 0.74 | *FCGBP* | 16.36 | 1.00 |
| *TNR* | 18.18 | 0.60 | *ADAMTS12* | 12.73 | 0.74 | *KCNB2* | 16.36 | 1.00 |
| *SPTA1* | 19.09 | 0.63 | *WDR49* | 12.73 | 0.74 | *ROS1* | 16.36 | 1.00 |
| *ERBB4* | 19.09 | 0.63 | *SLC4A10* | 12.73 | 0.74 | *MUC4* | 15.45 | 1.00 |
| *TPTE* | 19.09 | 0.63 | *TCHHL1* | 12.73 | 0.74 | *NLRP13* | 15.45 | 1.00 |
| *DNAH7* | 22.73 | 0.65 | *ACSM2B* | 12.73 | 0.74 | *TAF1L* | 15.45 | 1.00 |
| *FMN2* | 22.73 | 0.65 | *UGT2B15* | 12.73 | 0.74 | *COL11A1* | 14.55 | 1.00 |
| *DNAH8* | 26.36 | 0.66 | *DOCK3* | 23.64 | 0.75 | *SLIT2* | 14.55 | 1.00 |
| *SPHKAP* | 26.36 | 0.66 | *FLG* | 27.27 | 0.75 | *ZNF831* | 14.55 | 1.00 |
| *CSMD1* | 30.00 | 0.66 | *PTPRT* | 14.55 | 0.76 | *PLCB4* | 14.55 | 1.00 |
| *SIPA1L1* | 10.91 | 0.73 | *DSP* | 14.55 | 0.76 | *ABCA12* | 14.55 | 1.00 |
| *HECW2* | 10.91 | 0.73 | *HMCN1* | 15.45 | 0.76 | *ZDBF2* | 14.55 | 1.00 |
| *RALGAPB* | 10.91 | 0.73 | *ADAMTS18* | 15.45 | 0.76 | *VCAN* | 14.55 | 1.00 |
| *OTOGL* | 10.91 | 0.73 | *CPAMD8* | 15.45 | 0.76 | *NLRP11* | 14.55 | 1.00 |
| *DDX60L* | 10.91 | 0.73 | *LRRIQ1* | 15.45 | 0.76 | *LAMA2* | 13.64 | 1.00 |
| *RFX6* | 10.91 | 0.73 | *LPA* | 15.45 | 0.76 | *COL4A1* | 13.64 | 1.00 |
| *SPOCK3* | 10.91 | 0.73 | *TENM4* | 15.45 | 0.76 | *TNN* | 13.64 | 1.00 |
| *PKD1L2* | 10.91 | 0.73 | *COL22A1* | 15.45 | 0.76 | *STXBP5L* | 13.64 | 1.00 |
| *COL12A1* | 11.82 | 0.73 | *TRPM6* | 15.45 | 0.76 | *DSC1* | 13.64 | 1.00 |
| *RNF213* | 11.82 | 0.73 | *CRB1* | 15.45 | 0.76 | *FAM135B* | 13.64 | 1.00 |
| *MACF1* | 11.82 | 0.73 | *TRIOBP* | 16.36 | 0.77 | *COL14A1* | 13.64 | 1.00 |
| *SYCP1* | 11.82 | 0.73 | *LCT* | 16.36 | 0.77 | *MYH4* | 13.64 | 1.00 |
| *SLITRK6* | 11.82 | 0.73 | *SCN3A* | 16.36 | 0.77 | *CNGB3* | 13.64 | 1.00 |
| *PPP1R3A* | 11.82 | 0.73 | *MYO3A* | 16.36 | 0.77 | *ADAM2* | 13.64 | 1.00 |
| *SLC9C2* | 11.82 | 0.73 | *PCLO* | 35.45 | 0.79 | *SDK1* | 13.64 | 1.00 |
| *PRDM9* | 11.82 | 0.73 | *ZNF729* | 20.00 | 0.83 | *EPHA6* | 13.64 | 1.00 |
| *F5* | 11.82 | 0.73 | *DSCAM* | 23.64 | 0.84 | *MTUS2* | 13.64 | 1.00 |
| *NLRP2* | 11.82 | 0.73 | *TENM3* | 24.55 | 0.85 | *KCNH7* | 13.64 | 1.00 |
| *FCRL5* | 11.82 | 0.73 | *DNAH11* | 27.27 | 0.86 | *MYH15* | 13.64 | 1.00 |
| *NLRP5* | 11.82 | 0.73 | *ZNF208* | 19.09 | 0.93 | *EPPK1* | 12.73 | 1.00 |
| *GK2* | 11.82 | 0.73 | *FBN3* | 19.09 | 0.93 | *GRM8* | 12.73 | 1.00 |
| *KMT2D* | 11.82 | 0.73 | *BRAF* | 36.36 | 0.93 | *TIAM1* | 12.73 | 1.00 |
| *ADAM28* | 11.82 | 0.73 | *RYR3* | 22.73 | 0.94 | *USP29* | 12.73 | 1.00 |
| *PTPRN2* | 11.82 | 0.73 | *DNAH10* | 21.82 | 0.95 | *CTNND2* | 11.82 | 1.00 |
| *CACNA1D* | 11.82 | 1.00 | *PLCH1* | 10.91 | 1.00 | *FAM47C* | 10.00 | 1.00 |
| *SACS* | 11.82 | 1.00 | *ADAM7* | 10.91 | 1.00 | *FILIP1* | 10.00 | 1.00 |
| *LRRK2* | 11.82 | 1.00 | *CFHR4* | 10.91 | 1.00 | *LTBP2* | 10.00 | 1.00 |
| *MYH13* | 11.82 | 1.00 | *NCKAP5* | 10.91 | 1.00 | *TP63* | 10.00 | 1.00 |
| *CDH18* | 11.82 | 1.00 | *ANK1* | 10.91 | 1.00 | *MEGF10* | 10.00 | 1.00 |
| *RTTN* | 11.82 | 1.00 | *CHD3* | 10.91 | 1.00 | *TACR3* | 10.00 | 1.00 |
| *ARMC4* | 11.82 | 1.00 | *CACNA1A* | 10.91 | 1.00 | *ATP13A4* | 10.00 | 1.00 |
| *POTEF* | 11.82 | 1.00 | *EVPL* | 10.91 | 1.00 | *HHLA2* | 10.00 | 1.00 |
| *PLEC* | 10.91 | 1.00 | *PTPRC* | 10.91 | 1.00 | *PGK2* | 10.00 | 1.00 |
| *DNAH1* | 10.91 | 1.00 | *FNDC1* | 10.91 | 1.00 | *CDH8* | 10.00 | 1.00 |
| *DOCK2* | 10.91 | 1.00 | *UGT2B4* | 10.91 | 1.00 | *FRY* | 10.00 | 1.00 |
| *BSN* | 10.91 | 1.00 | *ZNF536* | 10.00 | 1.00 | *PTPN13* | 10.00 | 1.00 |
| *TLR4* | 10.91 | 1.00 | *HECTD4* | 10.00 | 1.00 | *THSD7A* | 10.00 | 1.00 |
| *PCDH18* | 10.91 | 1.00 | *LRRC7* | 10.00 | 1.00 | *AGBL1* | 10.00 | 1.00 |
| *BRINP1* | 10.91 | 1.00 | *CSPG4* | 10.00 | 1.00 | *GFRAL* | 10.00 | 1.00 |
| *TRPC4* | 10.91 | 1.00 |  |  |  |  |  |  |
