# Supplementary material for: Integrated genomic analysis identifies a genetic mutation model predicting response to immune checkpoint inhibitors in melanoma
Source: Cancer Med. 2020 Sep 24;9(22):8498–518. doi: 10.1002/cam4.3481 (PMC7666739; doi:10.1002/cam4.3481)
Supplement: Supplementary file 14 — Table S6 [file CAM4-9-8498-s014.docx]

| **Table S6. The detailed process of constructing the genetic mutation model in the Allen cohort** | | | | | | | |
| --- | --- | --- | --- | --- | --- | --- | --- |
|  | Variables | B | S.E, | Wals | df | Sig. | Exp (B) |
| Step 1 | *COL4A5* | -.252 | 1.156 | .048 | 1 | .827 | .777 |
|  | *NOTCH4* | .522 | 1.025 | .259 | 1 | .611 | 1.685 |
|  | *THSD7B* | .791 | .834 | .898 | 1 | .343 | 2.205 |
|  | *ABCA4* | 1.408 | 1.162 | 1.469 | 1 | .226 | 4.088 |
|  | *SYNE2* | 1.412 | 1.081 | 1.705 | 1 | .192 | 4.103 |
|  | *SCN1A* | 1.165 | 1.259 | .855 | 1 | .355 | 3.205 |
|  | *SLITRK3* | .529 | 1.113 | .226 | 1 | .634 | 1.698 |
|  | *FAM83B* | -.507 | .876 | .334 | 1 | .563 | .603 |
|  | *GRM3* | 1.432 | .814 | 3.092 | 1 | .079 | 4.185 |
|  | *EPHA7* | .726 | .922 | .620 | 1 | .431 | 2.067 |
|  | *HYDIN* | .206 | .852 | .058 | 1 | .809 | 1.229 |
|  | *NLRP10* | -1.672 | 1.369 | 1.492 | 1 | .222 | .188 |
|  | *ZNF804A* | -.080 | 1.255 | .004 | 1 | .949 | .923 |
|  | *COL4A4* | .356 | .985 | .130 | 1 | .718 | 1.428 |
|  | *PCDH15* | .620 | .820 | .571 | 1 | .450 | 1.859 |
|  | *FLNC* | 1.766 | 1.238 | 2.036 | 1 | .154 | 5.849 |
|  | *COL11A2* | -.138 | 1.090 | .016 | 1 | .899 | .871 |
|  | *MYO9A* | .747 | .975 | .587 | 1 | .444 | 2.111 |
|  | *NRXN1* | -.827 | 1.373 | .363 | 1 | .547 | .437 |
|  | *ANKRD30A* | .180 | 1.086 | .028 | 1 | .868 | 1.198 |
|  | *PRUNE2* | -.167 | .939 | .031 | 1 | .859 | .846 |
|  | *XIRP2* | -.115 | .801 | .021 | 1 | .886 | .892 |
|  | *SCN2A* | .532 | .973 | .298 | 1 | .585 | 1.702 |
|  | *ASPM* | .050 | 1.151 | .002 | 1 | .966 | 1.051 |
|  | *MYO5B* | .939 | 1.059 | .785 | 1 | .376 | 2.557 |
|  | constant | -2.731 | .519 | 27.644 | 1 | .000 | .065 |
| Step 2 | *COL4A5* | -.230 | 1.042 | .049 | 1 | .825 | .794 |
|  | *NOTCH4* | .535 | .979 | .299 | 1 | .585 | 1.707 |
|  | *THSD7B* | .789 | .833 | .897 | 1 | .344 | 2.201 |
|  | *ABCA4* | 1.409 | 1.162 | 1.471 | 1 | .225 | 4.092 |
|  | *SYNE2* | 1.405 | 1.068 | 1.728 | 1 | .189 | 4.074 |
|  | *SCN1A* | 1.162 | 1.260 | .851 | 1 | .356 | 3.197 |
|  | *SLITRK3* | .546 | 1.041 | .275 | 1 | .600 | 1.727 |
|  | *FAM83B* | -.507 | .876 | .335 | 1 | .563 | .603 |
|  | *GRM3* | 1.429 | .812 | 3.096 | 1 | .079 | 4.175 |
|  | *EPHA7* | .729 | .919 | .629 | 1 | .428 | 2.073 |
|  | *HYDIN* | .210 | .846 | .062 | 1 | .804 | 1.234 |
|  | *NLRP10* | -1.682 | 1.350 | 1.552 | 1 | .213 | .186 |
|  | *ZNF804A* | -.096 | 1.200 | .006 | 1 | .936 | .909 |
|  | *COL4A4* | .374 | .889 | .178 | 1 | .674 | 1.454 |
|  | *PCDH15* | .618 | .820 | .569 | 1 | .451 | 1.856 |
|  | *FLNC* | 1.754 | 1.205 | 2.121 | 1 | .145 | 5.778 |
|  | *COL11A2* | -.133 | 1.084 | .015 | 1 | .902 | .876 |
|  | *MYO9A* | .746 | .975 | .585 | 1 | .444 | 2.108 |
|  | *NRXN1* | -.800 | 1.225 | .427 | 1 | .514 | .449 |
|  | *ANKRD30A* | .177 | 1.086 | .027 | 1 | .870 | 1.194 |
|  | *PRUNE2* | -.165 | .939 | .031 | 1 | .860 | .848 |
|  | *XIRP2* | -.126 | .758 | .028 | 1 | .868 | .882 |
|  | *SCN2A* | .531 | .974 | .297 | 1 | .586 | 1.701 |
|  | *MYO5B* | .937 | 1.058 | .785 | 1 | .376 | 2.552 |
|  | constant | -2.730 | .519 | 27.681 | 1 | .000 | .065 |
| Step 3 | *COL4A5* | -.266 | .944 | .079 | 1 | .779 | .767 |
|  | *NOTCH4* | .531 | .979 | .294 | 1 | .588 | 1.700 |
|  | *THSD7B* | .792 | .831 | .909 | 1 | .340 | 2.208 |
|  | *ABCA4* | 1.365 | 1.021 | 1.788 | 1 | .181 | 3.915 |
|  | *SYNE2* | 1.421 | 1.049 | 1.833 | 1 | .176 | 4.139 |
|  | *SCN1A* | 1.155 | 1.257 | .844 | 1 | .358 | 3.173 |
|  | *SLITRK3* | .521 | .990 | .277 | 1 | .598 | 1.684 |
|  | *FAM83B* | -.523 | .850 | .378 | 1 | .539 | .593 |
|  | *GRM3* | 1.419 | .802 | 3.128 | 1 | .077 | 4.133 |
|  | *EPHA7* | .711 | .892 | .636 | 1 | .425 | 2.037 |
|  | *HYDIN* | .193 | .818 | .056 | 1 | .814 | 1.213 |
|  | *NLRP10* | -1.681 | 1.350 | 1.550 | 1 | .213 | .186 |
|  | *COL4A4* | .396 | .846 | .219 | 1 | .639 | 1.486 |
|  | *PCDH15* | .644 | .756 | .725 | 1 | .395 | 1.904 |
|  | *FLNC* | 1.776 | 1.173 | 2.294 | 1 | .130 | 5.909 |
|  | *COL11A2* | -.108 | 1.039 | .011 | 1 | .917 | .897 |
|  | *MYO9A* | .731 | .958 | .583 | 1 | .445 | 2.078 |
|  | *NRXN1* | -.815 | 1.214 | .451 | 1 | .502 | .443 |
|  | *ANKRD30A* | .179 | 1.089 | .027 | 1 | .870 | 1.196 |
|  | *PRUNE2* | -.185 | .908 | .041 | 1 | .839 | .831 |
|  | *XIRP2* | -.125 | .757 | .027 | 1 | .869 | .883 |
|  | *SCN2A* | .525 | .970 | .293 | 1 | .588 | 1.690 |
|  | *MYO5B* | .943 | 1.054 | .800 | 1 | .371 | 2.568 |
|  | constant | -2.732 | .518 | 27.764 | 1 | .000 | .065 |
| Step 4 | *COL4A5* | -.251 | .932 | .073 | 1 | .787 | .778 |
|  | *NOTCH4* | .524 | .978 | .287 | 1 | .592 | 1.689 |
|  | *THSD7B* | .780 | .822 | .899 | 1 | .343 | 2.181 |
|  | *ABCA4* | 1.355 | 1.016 | 1.776 | 1 | .183 | 3.875 |
|  | *SYNE2* | 1.385 | .994 | 1.940 | 1 | .164 | 3.995 |
|  | *SCN1A* | 1.183 | 1.223 | .936 | 1 | .333 | 3.265 |
|  | *SLITRK3* | .511 | .987 | .268 | 1 | .604 | 1.667 |
|  | *FAM83B* | -.499 | .819 | .371 | 1 | .542 | .607 |
|  | *GRM3* | 1.413 | .800 | 3.118 | 1 | .077 | 4.108 |
|  | *EPHA7* | .686 | .858 | .639 | 1 | .424 | 1.986 |
|  | *HYDIN* | .200 | .814 | .060 | 1 | .806 | 1.221 |
|  | *NLRP10* | -1.696 | 1.343 | 1.596 | 1 | .206 | .183 |
|  | *COL4A4* | .404 | .840 | .231 | 1 | .631 | 1.497 |
|  | *PCDH15* | .655 | .749 | .763 | 1 | .382 | 1.924 |
|  | *FLNC* | 1.784 | 1.170 | 2.324 | 1 | .127 | 5.952 |
|  | *MYO9A* | .743 | .954 | .606 | 1 | .436 | 2.102 |
|  | *NRXN1* | -.780 | 1.163 | .449 | 1 | .503 | .459 |
|  | *ANKRD30A* | .157 | 1.067 | .022 | 1 | .883 | 1.170 |
|  | *PRUNE2* | -.192 | .907 | .045 | 1 | .832 | .825 |
|  | *XIRP2* | -.144 | .734 | .039 | 1 | .844 | .866 |
|  | *SCN2A* | .510 | .958 | .283 | 1 | .595 | 1.665 |
|  | *MYO5B* | .915 | 1.017 | .810 | 1 | .368 | 2.497 |
|  | constant | -2.732 | .519 | 27.766 | 1 | .000 | .065 |
| Step 5 | *COL4A5* | -.198 | .858 | .053 | 1 | .817 | .820 |
|  | *NOTCH4* | .571 | .923 | .382 | 1 | .537 | 1.769 |
|  | *THSD7B* | .777 | .821 | .897 | 1 | .344 | 2.175 |
|  | *ABCA4* | 1.323 | .994 | 1.772 | 1 | .183 | 3.756 |
|  | *SYNE2* | 1.352 | .970 | 1.945 | 1 | .163 | 3.867 |
|  | *SCN1A* | 1.244 | 1.147 | 1.175 | 1 | .278 | 3.468 |
|  | *SLITRK3* | .546 | .960 | .324 | 1 | .569 | 1.727 |
|  | *FAM83B* | -.472 | .798 | .350 | 1 | .554 | .624 |
|  | *GRM3* | 1.423 | .798 | 3.182 | 1 | .074 | 4.150 |
|  | *EPHA7* | .676 | .854 | .626 | 1 | .429 | 1.966 |
|  | *HYDIN* | .222 | .797 | .078 | 1 | .780 | 1.249 |
|  | *NLRP10* | -1.733 | 1.318 | 1.727 | 1 | .189 | .177 |
|  | *COL4A4* | .400 | .840 | .227 | 1 | .634 | 1.492 |
|  | *PCDH15* | .671 | .740 | .821 | 1 | .365 | 1.956 |
|  | *FLNC* | 1.787 | 1.175 | 2.311 | 1 | .128 | 5.971 |
|  | *MYO9A* | .750 | .952 | .620 | 1 | .431 | 2.116 |
|  | *NRXN1* | -.782 | 1.167 | .448 | 1 | .503 | .458 |
|  | *PRUNE2* | -.184 | .907 | .041 | 1 | .839 | .832 |
|  | *XIRP2* | -.138 | .731 | .036 | 1 | .850 | .871 |
|  | *SCN2A* | .474 | .932 | .258 | 1 | .611 | 1.606 |
|  | *MYO5B* | .867 | .967 | .805 | 1 | .370 | 2.380 |
|  | constant | -2.733 | .519 | 27.766 | 1 | .000 | .065 |
| Step 6 | *COL4A5* | -.230 | .841 | .075 | 1 | .784 | .794 |
|  | *NOTCH4* | .601 | .903 | .443 | 1 | .506 | 1.825 |
|  | *THSD7B* | .768 | .820 | .876 | 1 | .349 | 2.155 |
|  | *ABCA4* | 1.322 | .995 | 1.763 | 1 | .184 | 3.750 |
|  | *SYNE2* | 1.387 | .951 | 2.129 | 1 | .144 | 4.004 |
|  | *SCN1A* | 1.227 | 1.139 | 1.162 | 1 | .281 | 3.411 |
|  | *SLITRK3* | .585 | .935 | .391 | 1 | .532 | 1.795 |
|  | *FAM83B* | -.490 | .792 | .382 | 1 | .537 | .613 |
|  | *GRM3* | 1.396 | .783 | 3.174 | 1 | .075 | 4.037 |
|  | *EPHA7* | .697 | .850 | .672 | 1 | .412 | 2.007 |
|  | *HYDIN* | .183 | .772 | .056 | 1 | .812 | 1.201 |
|  | *NLRP10* | -1.777 | 1.302 | 1.862 | 1 | .172 | .169 |
|  | *COL4A4* | .382 | .835 | .209 | 1 | .647 | 1.465 |
|  | *PCDH15* | .652 | .734 | .789 | 1 | .374 | 1.920 |
|  | *FLNC* | 1.767 | 1.167 | 2.295 | 1 | .130 | 5.856 |
|  | *MYO9A* | .719 | .936 | .591 | 1 | .442 | 2.053 |
|  | *NRXN1* | -.757 | 1.159 | .426 | 1 | .514 | .469 |
|  | *PRUNE2* | -.206 | .898 | .053 | 1 | .819 | .814 |
|  | *SCN2A* | .479 | .930 | .265 | 1 | .606 | 1.615 |
|  | *MYO5B* | .880 | .960 | .841 | 1 | .359 | 2.412 |
|  | constant | -2.742 | .518 | 28.067 | 1 | .000 | .064 |
| Step 7 | *COL4A5* | -.262 | .826 | .101 | 1 | .751 | .769 |
|  | *NOTCH4* | .600 | .904 | .440 | 1 | .507 | 1.822 |
|  | *THSD7B* | .795 | .812 | .958 | 1 | .328 | 2.214 |
|  | *ABCA4* | 1.353 | .986 | 1.884 | 1 | .170 | 3.869 |
|  | *SYNE2* | 1.341 | .928 | 2.085 | 1 | .149 | 3.822 |
|  | *SCN1A* | 1.191 | 1.133 | 1.104 | 1 | .293 | 3.291 |
|  | *SLITRK3* | .532 | .906 | .345 | 1 | .557 | 1.703 |
|  | *FAM83B* | -.496 | .790 | .395 | 1 | .530 | .609 |
|  | *GRM3* | 1.426 | .773 | 3.397 | 1 | .065 | 4.160 |
|  | *EPHA7* | .684 | .850 | .648 | 1 | .421 | 1.982 |
|  | *HYDIN* | .184 | .774 | .056 | 1 | .812 | 1.201 |
|  | *NLRP10* | -1.715 | 1.270 | 1.824 | 1 | .177 | .180 |
|  | *COL4A4* | .340 | .811 | .176 | 1 | .675 | 1.405 |
|  | *PCDH15* | .615 | .716 | .737 | 1 | .391 | 1.850 |
|  | *FLNC* | 1.790 | 1.152 | 2.412 | 1 | .120 | 5.987 |
|  | *MYO9A* | .754 | .924 | .666 | 1 | .414 | 2.125 |
|  | *NRXN1* | -.880 | 1.037 | .721 | 1 | .396 | .415 |
|  | *SCN2A* | .452 | .926 | .238 | 1 | .626 | 1.571 |
|  | *MYO5B* | .889 | .958 | .861 | 1 | .353 | 2.432 |
|  | constant | -2.748 | .517 | 28.207 | 1 | .000 | .064 |
| Step 8 | *COL4A5* | -.313 | .800 | .153 | 1 | .695 | .731 |
|  | *NOTCH4* | .609 | .907 | .452 | 1 | .501 | 1.840 |
|  | *THSD7B* | .831 | .799 | 1.082 | 1 | .298 | 2.295 |
|  | *ABCA4* | 1.362 | .989 | 1.896 | 1 | .169 | 3.903 |
|  | *SYNE2* | 1.364 | .926 | 2.167 | 1 | .141 | 3.910 |
|  | *SCN1A* | 1.205 | 1.130 | 1.136 | 1 | .287 | 3.336 |
|  | *SLITRK3* | .522 | .905 | .333 | 1 | .564 | 1.686 |
|  | *FAM83B* | -.489 | .792 | .381 | 1 | .537 | .613 |
|  | *GRM3* | 1.442 | .770 | 3.511 | 1 | .061 | 4.229 |
|  | *EPHA7* | .765 | .782 | .957 | 1 | .328 | 2.148 |
|  | *NLRP10* | -1.684 | 1.266 | 1.771 | 1 | .183 | .186 |
|  | *COL4A4* | .311 | .803 | .151 | 1 | .698 | 1.365 |
|  | *PCDH15* | .643 | .706 | .830 | 1 | .362 | 1.903 |
|  | *FLNC* | 1.833 | 1.139 | 2.587 | 1 | .108 | 6.250 |
|  | *MYO9A* | .684 | .879 | .605 | 1 | .437 | 1.982 |
|  | *NRXN1* | -.869 | 1.036 | .703 | 1 | .402 | .419 |
|  | *SCN2A* | .465 | .927 | .252 | 1 | .616 | 1.592 |
|  | *MYO5B* | .934 | .935 | .997 | 1 | .318 | 2.544 |
|  | constant | -2.730 | .512 | 28.419 | 1 | .000 | .065 |
| Step 9 | *COL4A5* | -.355 | .797 | .198 | 1 | .656 | .701 |
|  | *NOTCH4* | .582 | .905 | .413 | 1 | .520 | 1.790 |
|  | *THSD7B* | .820 | .798 | 1.056 | 1 | .304 | 2.270 |
|  | *ABCA4* | 1.476 | .944 | 2.445 | 1 | .118 | 4.377 |
|  | *SYNE2* | 1.349 | .933 | 2.092 | 1 | .148 | 3.855 |
|  | *SCN1A* | 1.179 | 1.126 | 1.097 | 1 | .295 | 3.251 |
|  | *SLITRK3* | .455 | .892 | .260 | 1 | .610 | 1.575 |
|  | *FAM83B* | -.377 | .739 | .261 | 1 | .609 | .686 |
|  | *GRM3* | 1.496 | .756 | 3.915 | 1 | .048 | 4.465 |
|  | *EPHA7* | .796 | .785 | 1.027 | 1 | .311 | 2.216 |
|  | *NLRP10* | -1.634 | 1.253 | 1.700 | 1 | .192 | .195 |
|  | *PCDH15* | .617 | .704 | .769 | 1 | .381 | 1.854 |
|  | *FLNC* | 1.867 | 1.137 | 2.694 | 1 | .101 | 6.467 |
|  | *MYO9A* | .735 | .872 | .709 | 1 | .400 | 2.085 |
|  | *NRXN1* | -.955 | 1.018 | .880 | 1 | .348 | .385 |
|  | *SCN2A* | .509 | .916 | .309 | 1 | .578 | 1.664 |
|  | *MYO5B* | .968 | .926 | 1.093 | 1 | .296 | 2.633 |
|  | constant | -2.713 | .508 | 28.468 | 1 | .000 | .066 |
| Step 10 | *NOTCH4* | .488 | .883 | .305 | 1 | .581 | 1.629 |
|  | *THSD7B* | .722 | .774 | .870 | 1 | .351 | 2.058 |
|  | *ABCA4* | 1.451 | .939 | 2.387 | 1 | .122 | 4.268 |
|  | *SYNE2* | 1.198 | .861 | 1.936 | 1 | .164 | 3.312 |
|  | *SCN1A* | 1.114 | 1.131 | .969 | 1 | .325 | 3.045 |
|  | *SLITRK3* | .434 | .886 | .240 | 1 | .624 | 1.543 |
|  | *FAM83B* | -.375 | .739 | .258 | 1 | .612 | .687 |
|  | *GRM3* | 1.476 | .753 | 3.842 | 1 | .050 | 4.376 |
|  | *EPHA7* | .726 | .770 | .891 | 1 | .345 | 2.068 |
|  | *NLRP10* | -1.465 | 1.194 | 1.505 | 1 | .220 | .231 |
|  | *PCDH15* | .572 | .696 | .675 | 1 | .411 | 1.772 |
|  | *FLNC* | 1.729 | 1.079 | 2.570 | 1 | .109 | 5.638 |
|  | *MYO9A* | .821 | .853 | .926 | 1 | .336 | 2.272 |
|  | *NRXN1* | -.951 | 1.014 | .879 | 1 | .348 | .386 |
|  | *SCN2A* | .452 | .913 | .245 | 1 | .621 | 1.572 |
|  | *MYO5B* | .911 | .914 | .994 | 1 | .319 | 2.488 |
|  | constant | -2.699 | .504 | 28.654 | 1 | .000 | .067 |
| Step 11 | *NOTCH4* | .484 | .882 | .301 | 1 | .583 | 1.623 |
|  | *THSD7B* | .683 | .766 | .796 | 1 | .372 | 1.980 |
|  | *ABCA4* | 1.566 | .898 | 3.040 | 1 | .081 | 4.790 |
|  | *SYNE2* | 1.137 | .848 | 1.799 | 1 | .180 | 3.119 |
|  | *SCN1A* | 1.190 | 1.113 | 1.145 | 1 | .285 | 3.288 |
|  | *FAM83B* | -.312 | .722 | .186 | 1 | .666 | .732 |
|  | *GRM3* | 1.449 | .752 | 3.709 | 1 | .054 | 4.259 |
|  | *EPHA7* | .709 | .763 | .864 | 1 | .353 | 2.033 |
|  | *NLRP10* | -1.398 | 1.181 | 1.400 | 1 | .237 | .247 |
|  | *PCDH15* | .594 | .696 | .729 | 1 | .393 | 1.811 |
|  | *FLNC* | 1.808 | 1.058 | 2.921 | 1 | .087 | 6.097 |
|  | *MYO9A* | .914 | .831 | 1.209 | 1 | .272 | 2.494 |
|  | *NRXN1* | -1.001 | 1.005 | .992 | 1 | .319 | .368 |
|  | *SCN2A* | .482 | .920 | .275 | 1 | .600 | 1.620 |
|  | *MYO5B* | .894 | .909 | .967 | 1 | .326 | 2.444 |
|  | constant | -2.688 | .503 | 28.585 | 1 | .000 | .068 |
| Step 12 | *NOTCH4* | .452 | .878 | .265 | 1 | .607 | 1.572 |
|  | *THSD7B* | .606 | .743 | .664 | 1 | .415 | 1.832 |
|  | *ABCA4* | 1.561 | .891 | 3.067 | 1 | .080 | 4.764 |
|  | *SYNE2* | 1.056 | .826 | 1.634 | 1 | .201 | 2.876 |
|  | *SCN1A* | 1.214 | 1.104 | 1.209 | 1 | .272 | 3.366 |
|  | *GRM3* | 1.367 | .728 | 3.521 | 1 | .061 | 3.922 |
|  | *EPHA7* | .631 | .741 | .725 | 1 | .395 | 1.879 |
|  | *NLRP10* | -1.366 | 1.176 | 1.349 | 1 | .245 | .255 |
|  | *PCDH15* | .564 | .691 | .666 | 1 | .414 | 1.757 |
|  | *FLNC* | 1.790 | 1.063 | 2.836 | 1 | .092 | 5.990 |
|  | *MYO9A* | .939 | .827 | 1.287 | 1 | .257 | 2.557 |
|  | *NRXN1* | -.951 | .994 | .916 | 1 | .338 | .386 |
|  | *SCN2A* | .375 | .879 | .182 | 1 | .670 | 1.455 |
|  | *MYO5B* | .856 | .903 | .898 | 1 | .343 | 2.354 |
|  | constant | -2.688 | .504 | 28.490 | 1 | .000 | .068 |
| Step 13 | *NOTCH4* | .413 | .871 | .225 | 1 | .635 | 1.512 |
|  | *THSD7B* | .668 | .726 | .845 | 1 | .358 | 1.950 |
|  | *ABCA4* | 1.609 | .882 | 3.328 | 1 | .068 | 4.999 |
|  | *SYNE2* | 1.050 | .826 | 1.616 | 1 | .204 | 2.859 |
|  | *SCN1A* | 1.369 | 1.032 | 1.760 | 1 | .185 | 3.932 |
|  | *GRM3* | 1.432 | .709 | 4.078 | 1 | .043 | 4.187 |
|  | *EPHA7* | .552 | .714 | .597 | 1 | .440 | 1.737 |
|  | *NLRP10* | -1.411 | 1.165 | 1.467 | 1 | .226 | .244 |
|  | *PCDH15* | .618 | .677 | .833 | 1 | .361 | 1.855 |
|  | *FLNC* | 1.807 | 1.058 | 2.920 | 1 | .088 | 6.095 |
|  | *MYO9A* | .962 | .829 | 1.347 | 1 | .246 | 2.617 |
|  | *NRXN1* | -.856 | .957 | .800 | 1 | .371 | .425 |
|  | *MYO5B* | .835 | .895 | .870 | 1 | .351 | 2.305 |
|  | constant | -2.694 | .505 | 28.403 | 1 | .000 | .068 |
| Step 14 | *THSD7B* | .749 | .702 | 1.138 | 1 | .286 | 2.114 |
|  | *ABCA4* | 1.583 | .876 | 3.264 | 1 | .071 | 4.871 |
|  | *SYNE2* | 1.165 | .789 | 2.176 | 1 | .140 | 3.205 |
|  | *SCN1A* | 1.376 | 1.028 | 1.792 | 1 | .181 | 3.960 |
|  | *GRM3* | 1.476 | .695 | 4.506 | 1 | .034 | 4.376 |
|  | *EPHA7* | .459 | .684 | .450 | 1 | .502 | 1.582 |
|  | *NLRP10* | -1.411 | 1.154 | 1.495 | 1 | .221 | .244 |
|  | *PCDH15* | .672 | .663 | 1.028 | 1 | .311 | 1.959 |
|  | *FLNC* | 1.846 | 1.054 | 3.068 | 1 | .080 | 6.333 |
|  | *MYO9A* | .987 | .821 | 1.445 | 1 | .229 | 2.682 |
|  | *NRXN1* | -.740 | .913 | .658 | 1 | .417 | .477 |
|  | *MYO5B* | .841 | .893 | .887 | 1 | .346 | 2.319 |
|  | constant | -2.705 | .507 | 28.525 | 1 | .000 | .067 |
| Step 15 | *THSD7B* | .758 | .701 | 1.168 | 1 | .280 | 2.134 |
|  | *ABCA4* | 1.598 | .871 | 3.366 | 1 | .067 | 4.942 |
|  | *SYNE2* | 1.261 | .787 | 2.571 | 1 | .109 | 3.530 |
|  | *SCN1A* | 1.379 | 1.015 | 1.846 | 1 | .174 | 3.972 |
|  | *GRM3* | 1.435 | .688 | 4.354 | 1 | .037 | 4.199 |
|  | *NLRP10* | -1.404 | 1.152 | 1.486 | 1 | .223 | .246 |
|  | *PCDH15* | .750 | .650 | 1.333 | 1 | .248 | 2.118 |
|  | *FLNC* | 1.844 | 1.066 | 2.991 | 1 | .084 | 6.319 |
|  | *MYO9A* | 1.023 | .828 | 1.525 | 1 | .217 | 2.781 |
|  | *NRXN1* | -.652 | .897 | .528 | 1 | .467 | .521 |
|  | *MYO5B* | .843 | .899 | .880 | 1 | .348 | 2.323 |
|  | constant | -2.658 | .494 | 28.953 | 1 | .000 | .070 |
| Step 16 | *THSD7B* | .731 | .703 | 1.083 | 1 | .298 | 2.078 |
|  | *ABCA4* | 1.509 | .869 | 3.019 | 1 | .082 | 4.524 |
|  | *SYNE2* | 1.300 | .781 | 2.770 | 1 | .096 | 3.670 |
|  | *SCN1A* | 1.466 | 1.006 | 2.122 | 1 | .145 | 4.331 |
|  | *GRM3* | 1.364 | .682 | 4.001 | 1 | .045 | 3.914 |
|  | *NLRP10* | -1.688 | 1.087 | 2.414 | 1 | .120 | .185 |
|  | *PCDH15* | .640 | .634 | 1.018 | 1 | .313 | 1.896 |
|  | *FLNC* | 1.684 | 1.028 | 2.685 | 1 | .101 | 5.385 |
|  | *MYO9A* | .891 | .799 | 1.245 | 1 | .265 | 2.439 |
|  | *MYO5B* | .827 | .896 | .852 | 1 | .356 | 2.286 |
|  | constant | -2.641 | .490 | 29.056 | 1 | .000 | .071 |
| Step 17 | *THSD7B* | .775 | .697 | 1.238 | 1 | .266 | 2.171 |
|  | *ABCA4* | 1.326 | .844 | 2.470 | 1 | .116 | 3.766 |
|  | *SYNE2* | 1.236 | .793 | 2.431 | 1 | .119 | 3.440 |
|  | *SCN1A* | 1.426 | .994 | 2.059 | 1 | .151 | 4.163 |
|  | *GRM3* | 1.263 | .665 | 3.604 | 1 | .058 | 3.535 |
|  | *NLRP10* | -1.409 | 1.026 | 1.886 | 1 | .170 | .244 |
|  | *PCDH15* | .741 | .620 | 1.431 | 1 | .232 | 2.099 |
|  | *FLNC* | 2.113 | .919 | 5.280 | 1 | .022 | 8.271 |
|  | *MYO9A* | .854 | .802 | 1.134 | 1 | .287 | 2.349 |
|  | constant | -2.571 | .473 | 29.546 | 1 | .000 | .076 |
| Step 18 | *THSD7B* | .729 | .698 | 1.092 | 1 | .296 | 2.073 |
|  | *ABCA4* | 1.353 | .842 | 2.580 | 1 | .108 | 3.869 |
|  | *SYNE2* | 1.366 | .766 | 3.185 | 1 | .074 | 3.921 |
|  | *SCN1A* | 1.391 | .995 | 1.954 | 1 | .162 | 4.020 |
|  | *GRM3* | 1.264 | .655 | 3.727 | 1 | .054 | 3.541 |
|  | *NLRP10* | -1.322 | 1.021 | 1.676 | 1 | .195 | .267 |
|  | *PCDH15* | .838 | .606 | 1.913 | 1 | .167 | 2.311 |
|  | *FLNC* | 2.184 | .897 | 5.929 | 1 | .015 | 8.881 |
|  | constant | -2.519 | .468 | 29.018 | 1 | .000 | .081 |
| Step 19 | *ABCA4* | 1.522 | .818 | 3.462 | 1 | .063 | 4.581 |
|  | *SYNE2* | 1.221 | .744 | 2.695 | 1 | .101 | 3.389 |
|  | *SCN1A* | 1.773 | .913 | 3.772 | 1 | .052 | 5.888 |
|  | *GRM3* | 1.291 | .657 | 3.864 | 1 | .049 | 3.637 |
|  | *NLRP10* | -1.205 | 1.009 | 1.426 | 1 | .232 | .300 |
|  | *PCDH15* | .903 | .601 | 2.257 | 1 | .133 | 2.466 |
|  | *FLNC* | 2.112 | .888 | 5.651 | 1 | .017 | 8.265 |
|  | constant | -2.418 | .450 | 28.925 | 1 | .000 | .089 |
| Step 20 | *ABCA4* | 1.204 | .776 | 2.406 | 1 | .121 | 3.335 |
|  | *SYNE2* | 1.112 | .739 | 2.265 | 1 | .132 | 3.042 |
|  | *SCN1A* | 1.165 | .783 | 2.216 | 1 | .137 | 3.207 |
|  | *GRM3* | 1.034 | .616 | 2.814 | 1 | .093 | 2.812 |
|  | *PCDH15* | .704 | .580 | 1.472 | 1 | .225 | 2.022 |
|  | *FLNC* | 1.664 | .806 | 4.265 | 1 | .039 | 5.282 |
|  | constant | -2.267 | .410 | 30.584 | 1 | .000 | .104 |
| Step 21 | *ABCA4* | 1.419 | .760 | 3.486 | 1 | .062 | 4.134 |
|  | *SYNE2* | 1.170 | .740 | 2.501 | 1 | .114 | 3.221 |
|  | *SCN1A* | 1.180 | .772 | 2.338 | 1 | .126 | 3.254 |
|  | *GRM3* | 1.202 | .594 | 4.090 | 1 | .043 | 3.325 |
|  | *FLNC* | 1.519 | .788 | 3.716 | 1 | .054 | 4.569 |
|  | constant | -2.114 | .376 | 31.538 | 1 | .000 | .121 |
| Step 22 | *ABCA4* | 1.551 | .734 | 4.471 | 1 | .034 | 4.717 |
|  | *SYNE2* | 1.322 | .704 | 3.530 | 1 | .060 | 3.752 |
|  | *GRM3* | 1.291 | .575 | 5.046 | 1 | .025 | 3.635 |
|  | *FLNC* | 1.413 | .776 | 3.315 | 1 | .069 | 4.108 |
|  | constant | -2.024 | .363 | 31.002 | 1 | .000 | .132 |
| Step 23 | *THSD7B* | .995 | .593 | 2.818 | 1 | .093 | 2.704 |
|  | *ABCA4* | 1.204 | .782 | 2.368 | 1 | .124 | 3.333 |
|  | *SYNE2* | 1.390 | .731 | 3.615 | 1 | .057 | 4.016 |
|  | *GRM3* | 1.141 | .595 | 3.677 | 1 | .055 | 3.129 |
|  | *FLNC* | 1.553 | .801 | 3.757 | 1 | .053 | 4.726 |
|  | constant | -2.222 | .398 | 31.243 | 1 | .000 | .108 |
| Step 24 | *THSD7B* | 1.234 | .558 | 4.896 | 1 | .027 | 3.435 |
|  | *SYNE2* | 1.640 | .713 | 5.284 | 1 | .022 | 5.153 |
|  | *GRM3* | .948 | .578 | 2.693 | 1 | .101 | 2.581 |
|  | *FLNC* | 1.636 | .767 | 4.552 | 1 | .033 | 5.137 |
|  | constant | -2.122 | .382 | 30.861 | 1 | .000 | .120 |
