# Supplementary material for: Integrated genomic analysis identifies a genetic mutation model predicting response to immune checkpoint inhibitors in melanoma
Source: Cancer Med. 2020 Sep 24;9(22):8498–518. doi: 10.1002/cam4.3481 (PMC7666739; doi:10.1002/cam4.3481)
Supplement: Supplementary file 15 — Table S7 [file CAM4-9-8498-s015.docx]

**Table S7. Biological functions of the genes in the genetic mutation model**

| **Gene name** | **Gene ID*** | **Gene function Summary^#^** | **Representative studies** |
| --- | --- | --- | --- |
| ***THSD7B***  (Thrombospondin Type 1 Domain Containing 7B) | 80731 | *THSD7B* is a Protein Coding gene. Diseases associated with *THSD7B* include Melanoma, Cutaneous Malignant 1. Among its related pathways are Metabolism of proteins and O-glycosylation of TSR domain-containing proteins. | Several GWAS studies revealed the association of SNPs in *THSD7B* and multiple diseases, including alcohol dependence [1], NSCLC [2], and uterine leiomyoma [3]  Hayashi, et al. identified *THSD7B* as one of the candidate genes for resistance to cisplatin / 5-FU in human esophageal squamous cell carcinoma [4]. |
| ***SYNE2***  (Spectrin repeat containing nuclear envelope protein 2) | 23224 | The protein encoded by this gene is a nuclear outer membrane protein, named as Nesprin-2, binding cytoplasmic F-actin. This binding tethers the nucleus to the cytoskeleton and aids in the maintenance of the structural integrity of the nucleus. | Lüke, et al. reported that Nesprin-2 Giant (NUANCE) maintained nuclear envelope architecture and composition in skin [5].  Warren, et al. reported that Nesprin-2-dependent pathway regulates the DNA damage response in vascular smooth muscle cell ageing [6]. |
| ***GRM3***  (Spectrin repeat containing nuclear envelope protein 2) | 2913 | The protein encoded by this gene is one of the members in the metabotropic glutamate receptors. The metabotropic glutamate receptors are a family of G protein-coupled receptors, that have been divided into 3 groups on the basis of sequence homology, putative signal transduction mechanisms, and pharmacologic properties. Group I includes GRM1 and GRM5 and these receptors have been shown to activate phospholipase C. Group II includes GRM2 and GRM3 while Group III includes GRM4, GRM6, GRM7 and GRM8. Group II and III receptors are linked to the inhibition of the cyclic AMP cascade but differ in their agonist selectivity. | Qian, et al. found that blood-based mutation of *GRM3* were associated with response to immunotherapy in NSCLC [7].  Neto, et al. reported that oncogenic *GRM3* variants dysregulate cAMP signaling, which had been implicated in melanoma progression and drug resistance [8].  Krauthammer, et al identified *GRM3* as one of the genes with a high mutation burden in sun-exposed melanomas [9]. |
| ***FLNC***  (Filamin C) | 2318 | This gene encodes one of three related filamin genes, specifically gamma filamin. These filamin proteins crosslink actin filaments into orthogonal networks in cortical cytoplasm and participate in the anchoring of membrane proteins for the actin cytoskeleton. | Large amounts of studies identified *FLNC* as a key role in myopathies and cardiomyopathies [10-12].  Several studies revealed *FLNC* was involved in multiple cancers, such as prostate cancer [13], gastric cancer [14], and hepatocellular carcinoma [15]. |

*derived from: <https://www.ncbi.nlm.nih.gov/gene>; ^#^ derived from: <http://www.genecards.org>;

Reference

[1] Wang KS, Liu X, Zhang Q, Pan Y, Aragam N, Zeng M. A meta-analysis of two genome-wide association studies identifies 3 new loci for alcohol dependence. J Psychiatr Res. 2011;45(11):1419-1425. doi:10.1016/j.jpsychires.2011.06.005

[2] Lee Y, Yoon KA, Joo J, et al. Prognostic implications of genetic variants in advanced non-small cell lung cancer: a genome-wide association study. Carcinogenesis. 2013;34(2):307-313. doi:10.1093/carcin/bgs356

[3] Aissani B, Zhang K, Wiener H. Evaluation of GWAS candidate susceptibility loci for uterine leiomyoma in the multi-ethnic NIEHS uterine fibroid study. Front Genet. 2015;6:241. Published 2015 Jul 14. doi:10.3389/fgene.2015.00241

[4] Hayashi M, Kawakubo H, Fukuda K, et al. THUMP domain containing 2 protein possibly induces resistance to cisplatin and 5-fluorouracil in in vitro human esophageal squamous cell carcinoma cells as revealed by transposon activation mutagenesis. J Gene Med. 2019;21(12):e3135. doi:10.1002/jgm.3135

[5] Lüke Y, Zaim H, Karakesisoglou I, et al. Nesprin-2 Giant (NUANCE) maintains nuclear envelope architecture and composition in skin. J Cell Sci. 2008;121(11):1887-1898. doi:10.1242/jcs.019075

[6] Warren DT, Tajsic T, Porter LJ, et al. Nesprin-2-dependent ERK1/2 compartmentalisation regulates the DNA damage response in vascular smooth muscle cell ageing. Cell Death Differ. 2015;22(9):1540-1550. doi:10.1038/cdd.2015.12

[7] Qian J, Nie W, Lu J, et al. Racial differences in characteristics and prognoses between Asian and white patients with nonsmall cell lung cancer receiving atezolizumab: An ancillary analysis of the POPLAR and OAK studies. Int J Cancer. 2020;146(11):3124-3133. doi:10.1002/ijc.32717

[8] Neto A, Ceol CJ. Melanoma-associated GRM3 variants dysregulate melanosome trafficking and cAMP signaling. Pigment Cell Melanoma Res. 2018;31(1):115-119. doi:10.1111/pcmr.12610

[9] Krauthammer M, Kong Y, Ha BH, et al. Exome sequencing identifies recurrent somatic RAC1 mutations in melanoma. Nat Genet. 2012;44(9):1006-1014. doi:10.1038/ng.2359

[10] Verdonschot JAJ, Vanhoutte EK, Claes GRF, et al. A mutation update for the FLNC gene in myopathies and cardiomyopathies. Hum Mutat. 2020;41(6):1091-1111. doi:10.1002/humu.24004

[11] Pecorari I, Mestroni L, Sbaizero O. Current Understanding of the Role of Cytoskeletal Cross-Linkers in the Onset and Development of Cardiomyopathies. Int J Mol Sci. 2020;21(16):E5865. doi:10.3390/ijms21165865

[12] Oz S, Yonath H, Visochyk L, et al. Reduction in Filamin C transcript is associated with arrhythmogenic cardiomyopathy in Ashkenazi Jews. Int J Cardiol. 2020;S0167-5273(19)36064-4. doi:10.1016/j.ijcard.2020.04.005

[13] Liu S, Wang W, Zhao Y, Liang K, Huang Y. Identification of Potential Key Genes for Pathogenesis and Prognosis in Prostate Cancer by Integrated Analysis of Gene Expression Profiles and the Cancer Genome Atlas. Front Oncol. 2020;10:809. doi:10.3389/fonc.2020.00809

[14] Wang J, Ding Y, Wu Y, Wang X. Identification of the complex regulatory relationships related to gastric cancer from lncRNA-miRNA-mRNA network. J Cell Biochem. 2020;121(1):876-887. doi:10.1002/jcb.29332

[15] Yang B, Liu Y, Zhao J, et al. Ectopic overexpression of filamin C scaffolds MEK1/2 and ERK1/2 to promote the progression of human hepatocellular carcinoma. Cancer Lett. 2017;388:167-176. doi:10.1016/j.canlet.2016.11.037
