# Supplementary material for: Integrated genomic analysis identifies a genetic mutation model predicting response to immune checkpoint inhibitors in melanoma
Source: Cancer Med. 2020 Sep 24;9(22):8498–518. doi: 10.1002/cam4.3481 (PMC7666739; doi:10.1002/cam4.3481)
Supplement: Supplementary file 16 — Supplementary Material [file CAM4-9-8498-s016.docx]

**Supplementary Figure Legends**

**Supplementary Figure S1. The** **workflow diagram of literature retrieval.**

**Supplementary Figure S2. The association between ITS and NAL in melanoma patients treated with ICIs.** The comparison of NAL in low and high ITS groups was performed by Mann-Whitney test. ICIs, immune-checkpoint inhibitors; ITS, immunotherapy score; NAL, neoantigen load; ****P* < 0.001.

**Supplementary Figure S3. GSEA analysis for hallmark genesets in subgroups with high and low ITS.** (**a**) TNF-α Signaling Via NF-ΚB. (**b**) Interferon-γ Response. (**c**) Allograft Rejection. (**d**) Interferon-αResponse. (**e**) IL-2/STAT5 Signaling. (**f**) IL-6/JAK/STAT3 Signaling. (**g**) G2M Checkpoint. (**h**) E2F Targets. (**i**) Apoptosis. (**j**) UV Response Up. NES, normalized enrichment score; FDR, false discovery rate.

**Supplementary Figure S4. The association between TMB and response to ICIs therapy in melanoma patients grouped by the upper quartile of TMB.** (**a**) Clinical benefit from ICIs therapy grouped by TMB in the Allen cohort, Snyder cohort and Liu cohort, using the upper quartile of TMB as a grouping threshold. (**b**) Forest plot showing the results of univariate logistic regression and meta-analysis for durable clinical benefit in the Allen cohort, Snyder cohort and Liu cohort, using the upper quartile of TMB as a grouping threshold. (**c**) The Kaplan–Meier curves for overall survival stratified by TMB in the Allen cohort, Snyder cohort and Liu cohort, using the upper quartile of TMB as a grouping threshold. (**d**) The Kaplan–Meier curves for progression-free survival stratified by TMB in the Allen cohort and Liu cohort, using the upper quartile of TMB as a grouping threshold. TMB, tumor mutation burden; DCB, durable clinical benefit; NCB, no clinical benefit; ns, no significance; OR, odds ratio; CI, confidence interval; OS, overall survival; PFS, progression-free survival.

**Supplementary Figure S5. The impact of ITS on overall survival of melanoma patients without ICIs therapy.** (**a**) The Kaplan–Meier curve for overall survival stratified by ITS in TCGA-SKCM cohort. (**b**) The Kaplan–Meier curve for overall survival stratified by ITS in ICGC-MELA cohort.

**Supplementary Figure S6. The overall alteration rate of ITS-related genes including *THSD7B*, *SYNE2*, *GRM3* and *FLNC* in TCGA Pan-Caner cohort.** At least one gene mutation in the same patient was considered as alteration. Otherwise, it was considered as no alteration.

**Supplementary Figure S7. The association between ITS and response to ICIs therapy in NSCLC patients of the Hellman cohort and Miao cohort.** (**a**) Clinical benefit from ICIs therapy grouped by ITS in squamous type and non-squamous type of NSCLC. (**b**) The Kaplan–Meier curve for progression-free survival stratified by ITS in non-squamous type of NSCLC patients. (**c**) The Kaplan–Meier curve for progression-free survival stratified by ITS in non-squamous type of NSCLC patients. ITS, immunotherapy score; PFS, progression-free survival; NSCLC, non-small cell lung cancer.

**Supplementary Figure S8. The association between TIDE and outcomes in melanoma patients treated with ICIs.** (**a**) ROC curve analysis for predicting no clinical benefit by TIDE in the Allen cohort, Snyder cohort and Liu cohort, respectively. (**b**) The Kaplan–Meier curves for overall survival stratified by TIDE in the Allen cohort, Snyder cohort and Liu cohort, respectively. (**c**) The Kaplan–Meier curves for progression-free survival stratified by TIDE in the Allen cohort and Liu cohort, respectively. In this study, the cut-off value of TIDE was consistent with that in the previous study (Jiang, P. et al). NCB, no clinical benefit; TIDE, Tumor Immune Dysfunction and Exclusion.
